# Metagenomics and Metagenome-Assembled Genomes: Analysis of *Cupei* from Sichuan Baoning Vinegar, One of the Four Traditional Renowned Vinegars in China

**DOI:** 10.3390/foods14030398

**Published:** 2025-01-26

**Authors:** Jie Wu, Ning Zhao, Qin Li, Kui Zhao, Meiling Tu, Jianlong Li, Kaidi Hu, Shujuan Chen, Shuliang Liu, Aiping Liu

**Affiliations:** College of Food Science, Sichuan Agricultural University, Ya’an 625014, China

**Keywords:** metagenomic sequencing, metagenome binning, MAGs, microbial diversity, Baoning vinegar

## Abstract

The microbial community in vinegar has primarily been investigated by analyzing short reads to determine operational taxonomic units, but it is also crucial to identify metagenome-assembled genomes (MAGs). In this study, the microbial diversity and functionality in Sichuan Baoning vinegar were examined through deep metagenomic sequencing and metagenomic binning. Results revealed that the most prevalent phylum was Firmicutes, followed by Proteobacteria and unclassified Bacteria. The most abundant bacterial species was *Acetilactobacillus jinshanensis*, while *Saccharomyces cerevisiae* was the most prevalent fungal species. The predominant viral species were *Hopescreekvirus LfeInf*, *Myoviridae* sp., and *Siphoviridae* sp. A total of 1395 MAGs were reconstructed, with 660 of them annotated. The majority of MAGs resolved at the species level were attributed to Firmicutes (n = 308), with *Acetilactobacillus jinshanensis* being the most abundant. According to the average nucleotide identity values, 223 out of the 660 MAGs might represent novel species. The recovered MAGs exhibited biomarker genes indicative of the genetic potential to encode several important secondary metabolites. This study helps to uncover the microbial composition and functional potential of microbial genomes in Sichuan Baoning vinegar.

## 1. Introduction

Vinegar is one of the most common acetic acid-diluted solution products consumed globally [1,2]. With a legacy spanning over 3000 years, Chinese cereal vinegar, produced mainly through solid-state fermentation, has become an essential component of Chinese culinary traditions due to its unique flavor [3]. The production process of cereal vinegar is highly distinctive, where the main ingredients—glutinous rice, sorghum, wheat bran, etc.—undergo multiple stages of fermentation, aging, and flavoring [4,5]. There are four traditional renowned vinegars in China: Shanxi aged vinegar, Zhenjiang aroma vinegar, Fujian Monascus vinegar, and Sichuan Baoning vinegar [6]. Sichuan Baoning vinegar is a representative type of Sichuan bran vinegar, known for its use of wheat bran as the primary ingredient. It is produced in various regions across Sichuan, each with distinct characteristics resulting from differences in processing techniques and local environments. The production process of Sichuan Baoning vinegar has previously been outlined in our report [7].

Cereal vinegar fermentation is heavily dependent on several microorganisms, such as molds, yeasts, lactic acid bacteria, and acetic acid bacteria. Both traditional culture methods [8,9] and culture-independent techniques [10,11] have been tried to investigate the microbiota present in vinegar fermentation. It is challenging to evaluate the microbial diversity in vinegar fermentation through isolation in culture media alone, leading to the prevalent application of high-throughput sequencing in recent years. However, identifying microorganisms in fermented foods through sequencing based on short reads (operational taxonomic units) has primarily relied on similarity searches in public databases, potentially resulting in inaccurate taxonomical attribution [12]. Additionally, it is important to note that the reference sequences may demonstrate varying functional traits compared to those found in vinegar, despite belonging to closely related phylogenetic groups [13].

Genome-resolved metagenomics, as a bioinformatics technique, facilitates the de novo assembly of almost complete genomes from metagenomic sequencing data [14]. This approach, focusing on genomes, proves valuable in investigating phylogenetic and metabolic diversity at the species or strain level, without the need for culture-dependent methods. To the best of our knowledge, detailed information about the core abundant microorganisms at the genome level and their functional characteristics in fermented food like vinegar is limited. In the present study, *Cupei* samples from Sichuan Baoning vinegar were employed for microbiota composition analysis using deep metagenomic sequencing. Metagenomic binning was then employed to construct metagenome-assembled genomes (MAGs), enhancing comprehension of the genomic characteristics and functions of key microorganisms in vinegar. We believe this report represents the most thorough investigation of MAGs linked to Sichuan Baoning vinegar.

## 2. Materials and Methods

### 2.1. Sampling

During the months of July and August 2022, samples of *Cupei* (the mixture of all the ingredients used for fermentation) were obtained from Sichuan Baoning Vinegar Co., Ltd. (Langzhong, Sichuan, China). Specifically, 297 samples were obtained from three fermentation ponds (21#, 29#, 30#) at various time points (days 1, 5, 7, 9, 11, 13, 15, 17, 19, 21, 25) as per the method in our previous report [15]. On the same day, samples from each fermentation pond were mixed, and approximately 400 g of *Cupei* was aseptically sealed in sterile Ziplock bags, followed by transporting to our lab and freezing at −80 °C. Each sample was identified by the sampling day and the corresponding fermentation pond number (1 = 21#, 2 = 29#, 3 = 30#); for instance, BN05_1 indicated samples collected on day 5 from fermentation pond 21#.

### 2.2. DNA Extraction, Metagenomic Sequencing, Quality Control, and Taxonomic Analysis

The extraction of genomic DNA from each sample was carried out utilizing the E.Z.N.A.^®^ stool DNA Kit (Omega Bio-tek, Norcross, GA, USA) following the manufacturer’s protocols. Subsequently, the genomic DNA was fragmented to approximately 450 bp using the Covaris S220 Focused-ultrasonicator (Covaris, Woburn, MA, USA), followed by the construction of sequencing libraries with these fragments. Sequencing was performed on the Illumina HiSeq X platform in the paired-end 150 bp mode.

The raw sequence, deposited with the China National Center for Bioinformation (accession number: PRJCA026508), was subjected to quality trimming using Trimmomatic (version: 0.36) [16] (ILLUMINACLIP:adapters.fa:2:30:10 SLIDINGWINDOW:4:15 MINLEN:75) to eliminate adaptor contaminants and low-quality reads. The processed reads were aligned with the human genome (version: hg19) using the BWA mem algorithm with specific parameters (-M -k 32 -t 16, http://bio-bwa.sourceforge.net/bwa.shtml (accessed on 6 May 2024)). The reads that were free from host–genome contamination and low-quality data were identified as clean reads and utilized for further analysis. Contig preparation (>500 bp), ORF (open reading frame) prediction (>60 bp), non-redundant gene catalog construction, and taxonomic annotations proceeded, as reported by Ren et al. [17]. Taxonomic abundances were normalized by calculating the proportion of reads for a particular taxon relative to the total bacterial 16S rRNA reads.

### 2.3. Genome Binning

The metagenomic binning of each sample was performed with reference to our previous report [13]. Briefly, MetaBAT2 (version: 2.11.1) [18] was employed with default parameters to recover MAGs from assembly contigs (>2500 bp) of metagenomic data in the *Cupei*. The quality of MAGs was evaluated using CheckM (version: 1.1.1) [19] to determine their completeness and contamination levels. Subsequently, the taxonomic classification of the obtained MAGs was conducted through GTDB-Tk (version: 1.3.0) with the GTDB-TK reference database (version: 202) and default settings [20]. The average nucleotide identity (ANI) for all the annotated MAGs was calculated using FastANI (version: 1.3.1) [21].

### 2.4. Gene Prediction and Annotation of Microbial Genomes

MAGs with ≥50% completeness and <10% contamination were selected and then de-replicated using dRep (https://github.com/MrOlm/drep (accessed on 6 May 2024)). Gene prediction of the de-replicated MAGs was performed using METAProdigal (version: 2.6.3). Characterization of MAGs was conducted according to the protein-coding genes identified by Glimmer (version: 3.02) [22]. The resulting amino acid sequences were compared to dbCAN HMMdb v8 using Hmmscan (version: 3.1b2) to identify carbohydrate-active enzymes (CAZys) [23]. Furthermore, functional annotations were assigned by comparing the sequences to the KEGG pathway database and the eggNOG database through kofamscan (version: 1.2.0) [24] and eggnog-mapper (version: 5.0) [25], respectively. The putative secondary metabolites were analyzed from recovered genomes using AntiSMASH (antibiotics and secondary metabolite analysis shell) (version: 7.0) [26].

## 3. Results

### 3.1. Metagenomic Sequencing

After undergoing quality control filtering, 1189.57 Gbp of clean data (7,941,143,174 high-quality reads) was obtained, with an average of 36.05 Gbp of data (240,640,702.24 high-quality reads) per sample (Table S1). In total, 9,468,753 ORFs were identified across all samples, with an average length of 632.15 bp, and 4,208,515 contigs were obtained after filtering the contigs with a length less than 500 bp.

### 3.2. Microbiota Composition

**Figure 1:**
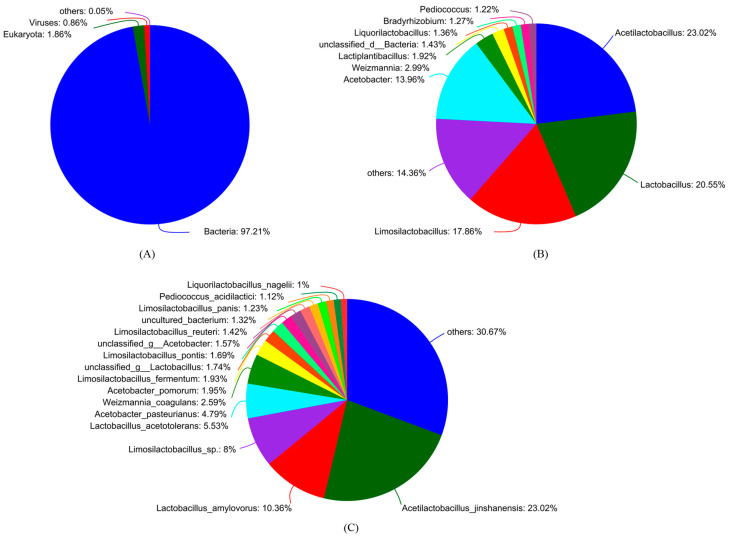
The microbiota composition of *Cupei*. (**A**) Domain level, where domains that have less than 0.5% relative abundance are grouped as ‘others’; (**B**) genus level, where genera that have less than 1% relative abundance are grouped as ‘others’; (**C**) species level, where species that have less than 1% relative abundance are grouped as ‘others’.

In the taxonomical classification of sequences, Bacteria were the predominant domain, succeeded by Eukaryotes and Viruses (Figure 1A). The top six genera were *Acetilactobacillus*, *Lactobacillus*, *Limosilactobacillus*, *Acetobacter*, *Weizmannia*, and *Lactiplantibacillus* (Figure 1B). *Acetilactobacillus jinshanensis* was determined as the most prevalent species, followed by *Lactobacillus amylovorus* and *Limosilactobacillus* sp. (Figure 1C).

**Figure 2:**
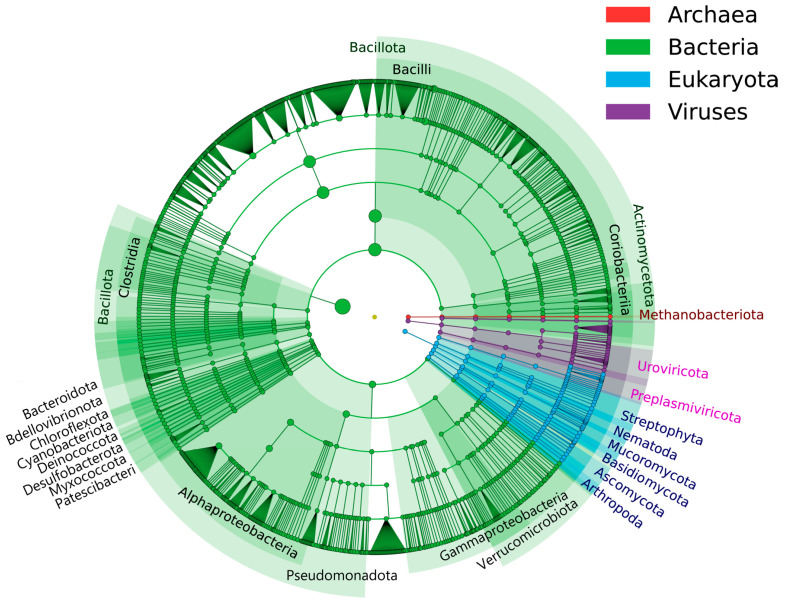
The phylogenetic tree of Archaea, Bacteria, Eukaryotes, and Viruses in *Cupei*.

**Figure 3:**
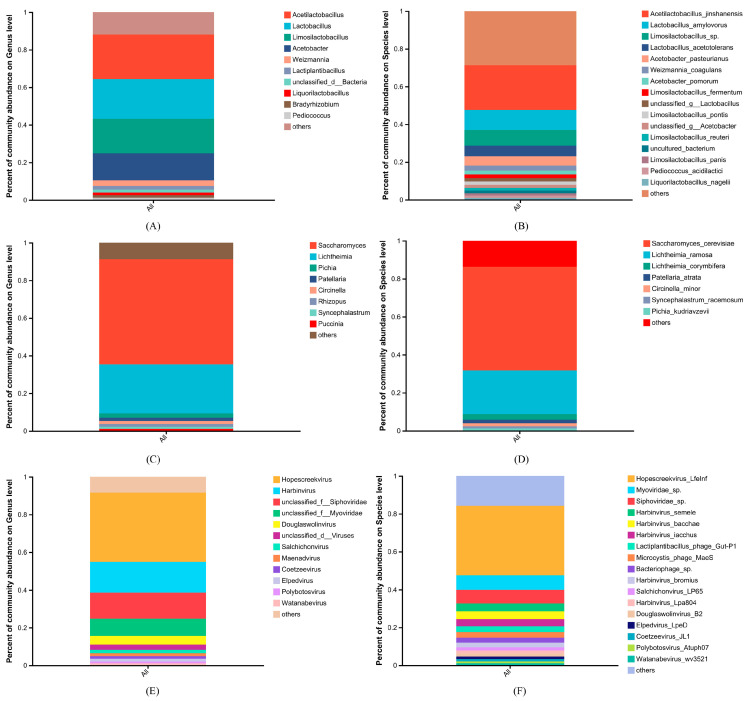
The percentages of bacterial, fungal, and viral community abundance in *Cupei*. (**A**) Bacterial community at the genus level; (**B**) bacterial community at the species level; (**C**) fungal community at the genus level; (**D**) fungal community at the species level; (**E**) viral community at the genus level; (**F**) viral community at the species level. Genus or species with less than 1% relative abundance are grouped as ‘others’.

A total of 120 bacterial phyla, consisting of 3415 genera and 22,817 species, were identified in the analyzed samples (Figure 2 and Figure 3). The predominant bacterial genera observed were *Acetilactobacillus*, *Lactobacillus*, and *Limosilactobacillus* (Figure 3A), with *Acetilactobacillus jinshanensis*, *Lactobacillus amylovorus*, *Limosilactobacillus* sp., *Lactobacillus acetotolerans*, and *Acetobacter pasteurianus* being the most abundant species (Figure 3B). All but *Acetilactobacillus jinshanensis* and *Limosilactobacillus* sp. were obtained using conventional microbial cultivation methods [9]. Similarly, *Acetilactobacillus jinshanensis* and *Lactobacillus acetotolerans* were the top two species in Zhenjiang aromatic vinegar [27].

A total of 10 fungal phyla were identified in the analyzed samples, consisting of 609 genera and 1476 species (Figure 2 and Figure 3). The predominant fungal genera observed included *Saccharomyces*, *Lichtheimia*, and *Pichia* (Figure 3C), with the most abundant species being *Saccharomyces cerevisiae*, *Lichtheimia ramosa*, *Lichtheimia corymbifera*, *Patellaria atrata*, and *Circinella minor* (Figure 3D). In contrast, in the process of alcohol and acetic acid fermentation in Shanxi aged vinegar, the predominant fungal genera were *Saccharomyces* and *Saccharomycopsis* [28]. A study on Zhenjiang aromatic vinegar revealed varying dominant fungal populations at different fermentation stages. Specifically, *Wickerhamomyces* and *Saccharomyces* dominated the starch saccharification and alcohol fermentation stages, respectively, while *Alternaria*, *Fusarium*, and *Saccharomyces* were predominant during acetic acid fermentation [27].

A total of 11 viral phyla, consisting of 302 genera and 869 species, were found in the analyzed samples (Figure 2 and Figure 3). The prevalent viral genera observed included *Hopescreekvirus*, *Harbinvirus*, and unclassified Siphoviridae (Figure 3E), with *Hopescreekvirus LfeInf*, *Myoviridae* sp., and *Siphoviridae* sp. being the predominant viral species (Figure 3F). Generally, the discussion of the viral community in vinegar fermentation has been limited, and our findings on viral composition demonstrated limited similarity to studies by Yu et al. [29].

### 3.3. Assembly of Draft Genomes

In the investigation of the genetic potential of microorganisms present in *Cupei* samples, metagenomic binning was conducted. Following contig assembly, a total of 1395 MAGs were reconstructed, with 336 classified as high-quality MAGs (completeness ≥ 90% and contamination ≤ 5%). The distribution of all the constructed MAGs across the 33 samples is detailed in Table S2. Taxonomic annotation of each MAG was carried out with GTDB-Tk, resulting in the annotation of 660 MAGs (Table S3).

A total of 658 MAGs were annotated to the class level, 658 to the order level, 658 to the family level, 627 to the genus level, and 433 to the species level. All annotated MAGs were affiliated with the domain Bacteria, with Firmicutes (n = 454) and Proteobacteria (n = 130) being the most prevalent phyla. Less abundant phyla included Bacteroidota (n = 41, all identified at the class level as Bacteroidia), Actinobacteriota (n = 25, all identified at the class level as Actinomycetia), Myxococcota (n = 7, all identified at the genus level as *Labilithrix*), and Deinococcota (n = 3, all identified at the genus level as *Meiothermus*). The Firmicutes group comprised the classes Bacilli (n = 451) and Negativicutes (n = 3), while Proteobacteria were predominantly represented by the class Alphaproteobacteria (n = 113), followed by Gammaproteobacteria (n = 17). According to the ANI values, 223 of these MAGs might be linked to novel species, with 31, 80, 30, and 26 novel species belonging to the family Amphibacillaceae, the genus *Limosilactobacillus*, the genus *Bradyrhizobium*, and the genus *Lactobacillus*, respectively.

The Firmicutes phylum accounted for the highest number of species-level resolved MAGs at 308, followed by Proteobacteria (n = 71), Actinobacteriota (n = 25), and Bacteroidota (n = 29). Within the phylum Firmicutes, specific MAGs were identified, including *Acetilactobacillus jinshanensis* (n = 33), *Lactobacillus amylovorus* (n = 32), *Weizmannia coagulans* (n = 31), and *Lactobacillus acetotolerans* (n = 30). In the phylum Proteobacteria, *Acetobacter pomorum* (n = 23) was the most abundant, followed by *Acetobacter peroxydans* (n = 13).

### 3.4. Functionality of Microbial Genomes

Upon de-replication of the 660 initially annotated MAGs, a final set of 90 MAGs was obtained. The relative abundance of MAGs in each sample was calculated using the quant_bins module in MetaWRAP, and a heatmap was created using Cloud Platform (http://www.cloud.biomicroclass.com/CloudPlatform (accessed on 6 May 2024)) (Figure S1). Additionally, a total of 251,224 genes were predicted through the METAProdigal tool.

**Figure 4:**
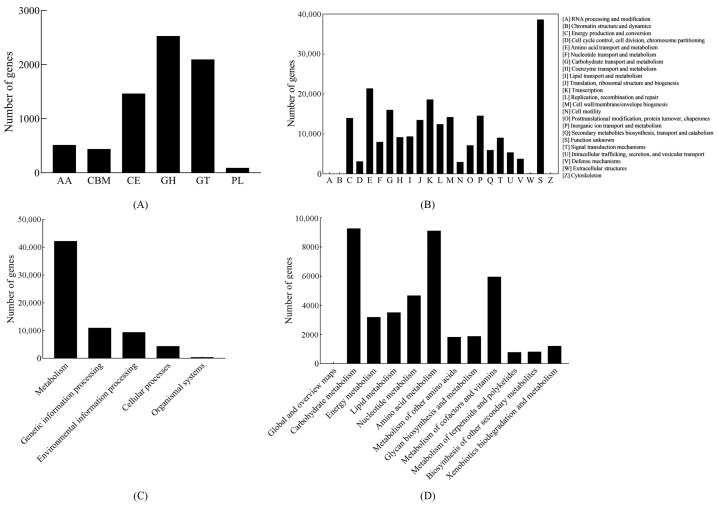
The functionality of de-replicated MAGs in *Cupei*. (**A**) Statistics for carbohydrate-active enzyme families in microbial genomes. (**B**) Statistics for COG classifications in microbial genomes. (**C**) Statistics for KEGG pathways in microbial genomes. (**D**) Statistics for KEGG pathway category ‘metabolism’ in microbial genomes.

The genetic potential of the 90 microbial genomes was analyzed. A total of 7134 genes were identified as participants in carbohydrate metabolism through CAZy database annotation, encompassing various CAZys such as carbohydrate esterases (CEs), glycosyltransferases (GTs), polysaccharide lyases (PLs), glycoside hydrolases (GHs), auxiliary activities (AAs), and carbohydrate-binding modules (CBMs). GHs were the most prevalent enzymes, constituting 35.42% of all carbohydrate-metabolizing genes and comprising 105 distinct families. Following GHs, GTs were the second most abundant enzymes with 43 families, covering 29.38% of the total carbohydrate-metabolizing genes, followed by CEs with 15 families (20.54%) (Figure 4A). GHs, GTs, and CEs were found in various phyla, with GHs predominantly present in MAGs of *Paenibacillus* sp. and *Chitinophaga* sp., GTs primarily in MAGs of *Burkholderia vietnamiensis*, and CEs mainly in MAGs of *Mycobacterium aubagnense*.

As shown in Figure 4B, a total of 227,676 genes were annotated using the eggNOG database. The cluster of orthologous groups (COG) annotation (including 23 COG categories) highlighted that the majority of proteins identified in MAGs were linked to metabolism, with cellular processes and signaling, as well as information storage and processing ranking subsequently.

In total, 211,665 predicted genes underwent KEGG database annotation, with 67,415 unigenes associated with KEGG pathways. Among these, 42,267 unigenes (62.70%) were categorized under metabolism, representing the highest proportion. In addition, 10,957 unigenes (16.25%) were related to genetic information processing, 9411 unigenes (13.96%) to environmental information processing, 4385 unigenes (6.50%) to cellular processes, and 395 unigenes (0.59%) to organismal systems (Figure 4C). In the level 2 classification of the metabolism category, carbohydrate metabolism represented the highest percentage at 21.94%, with amino acid metabolism, metabolism of cofactors and vitamins, and nucleotide metabolism ranking next at 21.56%, 14.09%, and 11.07%, respectively (Figure 4D).

Among the 90 MAGs, 69 were found to contain 358 genes that have genetic potential to encode 40 different types of secondary metabolites (Table S4). The predominant types of secondary metabolites were terpene (68 genes), other unspecified ribosomally synthesized and post-translationally modified peptide product (RiPP-like) (45 genes), non-ribosomal peptide synthetase (NRPS) (39 genes), type III polyketides (T3PKs) (38 genes), redox-cofactor (22 genes), and arylpolyene (22 genes). Terpene is known as one of the key indicators of aroma quality in vinegar, and its encoding gene was present in 32 MAGs. MAGs of *Chitinophaga* sp. exhibited the highest number of terpene synthesis genes, followed by *Streptomyces cacaoi*. Additionally, MAGs of all eleven different *Limosilactobacillus* sp. as well as *Limosilactobacillus pontis*, *Limosilactobacillus secaliphilus*, and *Limosilactobacillus panis* had genetic potential to produce T3PKs, indicating the possible antimicrobial ability of these lactic acid bacteria against pathogenic bacteria [30].

## 4. Discussion

In our preliminary investigation, high-throughput amplicon sequencing and cultivation techniques were employed to assess the microbial diversity of Sichuan Baoning vinegar. However, our understanding of the microbial community at the species level remains quite limited. As is known, metagenomic sequencing enhances taxonomic resolution to the species or strain level and offers insights into potential functional information [31]. By employing in-depth metagenomic sequencing technology, this study examined the species-level microbial diversity present in Sichuan Baoning vinegar, with an average sample size of 36.05 Gbp. The analysis revealed a total of 26,241 species, with bacteria being the predominant group. *Acetilactobacillus*, *Lactobacillus*, and *Limosilactobacillus* were the most abundant genera, with the most prevalent species being *Acetilactobacillus jinshanensis*, *Lactobacillus amylovorus*, and *Limosilactobacillus* sp. (Figure 3). Previously, *Lactobacillus* was identified as the predominant genus [32]. However, recent taxonomic updates on lactic acid bacteria have led to the recognition of *Acetilactobacillus* [33]. Interestingly, the results reported by Han et al. [34] and Tong et al. [35] differed, potentially due to the use of varying types of Sichuan bran vinegar. In addition to the well-known *Saccharomyces cerevisiae*, *Lichtheimia ramose* and *Lichtheimia corymbifera* were also predominant fungal species. Examination of microbial diversity at the species level will serve as a foundation for elucidating the key microorganisms engaged in the fermentation of Sichuan Baoning vinegar and for controlling fermentation procedures through the application of synergistic functional microbial consortia [36]. Notably, the fungal genus *Aspergillus* was not identified among the dominant microorganisms, a discrepancy from our previous outcomes of amplicon-based high-throughput sequencing [32]. This inconsistency may be attributed to variations in sample batches, DNA extraction, and sequencing methodologies.

Metagenomic binning techniques enable the reconstruction of high-quality MAGs from complex microbial communities, thereby enabling further exploration and utilization of microbial resources. In this study, 660 annotated MAGs were obtained by employing metagenomic binning, with 33 genomes identified as *Acetilactobacillus jinshanensis*. This microorganism plays a significant role in the production of vinegar and Baijiu [37]. However, research on the genomes and functionalities of *Acetilactobacillus jinshanensis* strains from different sources is limited and requires further exploration. The recovered MAGs demonstrated a variety of genetic potential, featuring biomarker genes indicative of the ability to encode various secondary metabolites, particularly terpene. This type of compound can play a crucial role in enhancing the flavor of vinegar [38].

This study uncovered several potential MAGs of new species, including the genera *Lactobacillus* and *Limosilactobacillus*, when utilizing metagenomic binning and ANI analysis. The newly identified species may possess unique genetic and metabolic traits that could significantly impact vinegar fermentation and product quality. By analyzing the nutritional requirements of strains through MAG data, these strains may be isolated via cultureomics. Further research is needed to gain a comprehensive comprehension of their capabilities.

Viruses are ubiquitous across all ecosystems, infecting organisms ranging from prokaryotes to eukaryotes [39]. Bacteriophages, viruses that target bacteria, have been reported in various fermented products such as milks, vinegars, and vegetables [40]. Studies suggested that certain bacteriophages played a role in shaping bacterial community succession during fermentation, ultimately enhancing the quality and sensory properties of the final food product [41]. The predominant bacteriophage families detected in fermented foods included Siphoviridae, Podoviridae, and Myoviridae [42]. Similarly, *Myoviridae* sp. and *Siphoviridae* sp. were noted as prevalent viral species in Sichuan Baoning vinegar. However, it is essential to recognize that the analysis of viral composition in these environments relied heavily on metagenomic sequencing, which might exhibit bias towards the extraction of bacterial and fungal DNA. Notably, the metagenomes only allowed the identification of DNA viruses, and RNA viruses may be also present in *Cupei*. In addition, future detection of CRISPRs in the MAGs and exploration through phagenomics may offer a more thorough insight into the viral composition and function.

## 5. Conclusions

The present study examined the microbial community and its functional potential in Sichuan Baoning vinegar using metagenomic sequencing. Firmicutes was the most predominant phylum, with the top six genera identified as *Acetilactobacillus*, *Lactobacillus*, *Limosilactobacillus*, *Acetobacter*, *Weizmannia*, and *Lactiplantibacillus*. A total of 1395 MAGs were assembled through genomic binning, with 660 MAGs annotated using GTDB-Tk. The majority of species-level resolved MAGs were annotated as *Acetilactobacillus jinshanensis* (n = 33), *Lactobacillus amylovorus* (n = 32), *Weizmannia coagulans* (n = 31), and *Lactobacillus acetotolerans* (n = 30). The recovered MAGs showed biomarker genes indicative of the genetic potential to encode several secondary metabolites. Additionally, MAGs of new species were obtained, highlighting the need for further application of culturomics techniques to recover these strains from *Cupei*. This study contributes to the understanding of the microbiota in *Cupei* and offers insights into the fermentation of Sichuan Baoning vinegar.

## Data Availability

The original contributions presented in the study are included in the article/Supplementary Materials, further inquiries can be directed to the corresponding author.

## Supplementary Materials

The following supporting information can be downloaded at https://www.mdpi.com/article/10.3390/foods14030398/s1, Figure S1: The abundance of de-replicated MAGs in *Cupei*. Each row represents a MAG and each column represents a sample. The color in the figure indicates the abundance of each MAG in that sample (log10 FPKM), ranging from blue (lower abundance) to red (higher abundance). Table S1: Metagenomic data obtained from *Cupei*; Table S2: The distribution of 1395 MAGs in *Cupei*; Table S3: Taxonomic annotation of MAGs using GTDB-Tk; Table S4: Prediction of secondary metabolite biosynthetic gene clusters from the MAGs using antiSMASH.
