# Supplementary material for: Metagenomics and Metagenome-Assembled Genomes: Analysis of Cupei from Sichuan Baoning Vinegar, One of the Four Traditional Renowned Vinegars in China
Source: Foods. 2025 Jan 26;14(3):398. doi: 10.3390/foods14030398 (PMC11816609; doi:10.3390/foods14030398)
Supplement: Supplementary file 1 [file foods-14-00398-s001.zip › Table S1.pdf]

Table S1 Metagenomic data obtained from *Cupei*.

Sample	Total reads	Total bases	ORFs	Contig number
BN01_1	212321340	31811016426	443871	173588
BN01_2	264353986	39608411546	642790	421821
BN01_3	253886292	38035102671	532870	245417
BN05_1	248267384	37202392385	308815	157970
BN05_2	251213192	37644689127	258998	131717
BN05_3	305048440	45721147101	375453	179324
BN07_1	178058164	26637027519	235291	100468
BN07_2	232014372	34711665085	238929	105373
BN07_3	211916352	31718737184	218613	104960
BN09_1	251280592	37657017802	255118	101994
BN09_2	306399884	45917970572	249800	104444
BN09_3	191945024	28764362390	244800	105615
BN11_1	180065638	26981449840	237938	104533
BN11_2	284797386	42662114111	231264	97270
BN11_3	292677218	43854994556	343014	155092
BN13_1	207746968	31129369521	199775	82502
BN13_2	209637294	31412260256	229805	97436
BN13_3	200231558	29953957900	225012	93345
BN15_1	231572752	34653236200	185251	82949
BN15_2	234944380	35206562491	262891	113478
BN15_3	281418278	42167120300	246578	108000
BN17_1	185035528	27724476377	193759	82317
BN17_2	320620018	48031722395	305761	125438
BN17_3	265112062	39726568656	223867	97134
BN19_1	342343788	51284781547	310908	132710
BN19_2	323091846	48411177764	389605	164338
BN19_3	128980346	19300858759	243792	100483
BN21_1	202835388	30390873212	222899	92702
BN21_2	228318346	34195700071	236514	94039
BN21_3	261585372	39175821887	275916	115426
BN25_1	227776374	34114541899	306702	115143
BN25_2	217823608	32628868958	298723	111697
BN25_3	207824004	31132968639	293431	109792
