# Supplementary material for: Metagenomics and Metagenome-Assembled Genomes: Analysis of Cupei from Sichuan Baoning Vinegar, One of the Four Traditional Renowned Vinegars in China
Source: Foods. 2025 Jan 26;14(3):398. doi: 10.3390/foods14030398 (PMC11816609; doi:10.3390/foods14030398)
Supplement: Supplementary file 1 [file foods-14-00398-s001.zip › Table S2.pdf]

Table S2 The distribution of 1,395 metagenome-assembled genomes in *Cupei*.

**Sample**	**high-quality (completeness>90% & contamination <5%)**	**medium-quality (completeness>50% & contamination <10%)**	**low-quality (completeness<50% & contamination <10%)**
BN01_1	13	12	42
BN01_2	7	13	40
BN01_3	9	17	41
BN05_1	9	13	26
BN05_2	8	9	32
BN05_3	11	10	41
BN07_1	4	13	35
BN07_2	5	11	30
BN07_3	6	11	24
BN09_1	7	12	35
BN09_2	8	10	32
BN09_3	10	9	36
BN11_1	10	9	19
BN11_2	8	10	21
BN11_3	11	11	25
BN13_1	7	8	15
BN13_2	7	11	26
BN13_3	11	13	20
BN15_1	9	6	15
BN15_2	11	7	22
BN15_3	11	9	19
BN17_1	9	7	11
BN17_2	11	12	18
BN17_3	11	8	9
BN19_1	13	11	18
BN19_2	17	8	18
BN19_3	10	6	10
BN21_1	9	9	8
BN21_2	12	5	14
BN21_3	14	6	7
BN25_1	17	6	10
BN25_2	15	9	12
BN25_3	16	7	10
