# Supplementary material for: Metagenomics and Metagenome-Assembled Genomes: Analysis of Cupei from Sichuan Baoning Vinegar, One of the Four Traditional Renowned Vinegars in China
Source: Foods. 2025 Jan 26;14(3):398. doi: 10.3390/foods14030398 (PMC11816609; doi:10.3390/foods14030398)
Supplement: Supplementary file 1 [file foods-14-00398-s001.zip › Table S3.pdf]

Table S3 Taxonomic annotation of metagenome-assembled genomes using GTDB-Tk.

Genome	Classification	Fastani_ref	Fastani_ani
MAGs.BN01_1.Bin10	d__Bacteria;p__Proteobacteria;c__Gammaproteobacteria;o__Enterobacterales;f__Enterobacteriaceae;g__Serratia_B;s__Serratia_B rubidaea	GCF_901472405.1	98.23
MAGs.BN01_1.Bin11	d__Bacteria;p__Firmicutes;c__Bacilli;o__Lactobacillales;f__Lactobacillaceae;g__Acetilactobacillus;s__Acetilactobacillus jinshanensis	GCF_004359375.1	99.97
MAGs.BN01_1.Bin14	d__Bacteria;p__Firmicutes;c__Bacilli;o__Bacillales_D;f__Amphibacillaceae;g__;s__	N/A	N/A
MAGs.BN01_1.Bin16	d__Bacteria;p__Actinobacteriota;c__Actinomycetia;o__Streptomycetales;f__Streptomycetaceae;g__Streptomyces;s__Streptomyces cacaoi	GCF_006539165.1	99.08
MAGs.BN01_1.Bin19	d__Bacteria;p__Firmicutes;c__Bacilli;o__Lactobacillales;f__Lactobacillaceae;g__Lactobacillus;s__Lactobacillus acetotolerans	GCF_001436775.1	99.15
MAGs.BN01_1.Bin2	d__Bacteria;p__Proteobacteria;c__Gammaproteobacteria;o__Enterobacterales;f__Enterobacteriaceae;g__Pantoea;s__Pantoea agglomerans	GCF_001598475.1	98.53
MAGs.BN01_1.Bin21	d__Bacteria;p__Firmicutes;c__Bacilli;o__Lactobacillales;f__Lactobacillaceae;g__Ligilactobacillus;s__Ligilactobacillus acidipiscis	GCF_001435755.1	98.08
MAGs.BN01_1.Bin26	d__Bacteria;p__Firmicutes;c__Bacilli;o__Lactobacillales;f__Lactobacillaceae;g__Lactobacillus;s__Lactobacillus amylovorus	GCF_002706375.1	98.99
MAGs.BN01_1.Bin30	d__Bacteria;p__Bacteroidota;c__Bacteroidia;o__Chitinophagales;f__Chitinophagaceae;g__Sediminibacterium;s__Sediminibacterium magnilacihabitans	GCA_002973575.1	98.38
MAGs.BN01_1.Bin31	d__Bacteria;p__Proteobacteria;c__Alphaproteobacteria;o__Acetobacterales;f__Acetobacteraceae;g__Acetobacter;s__Acetobacter lovaniensis	GCF_014207635.1	99.6
MAGs.BN01_1.Bin33	d__Bacteria;p__Firmicutes;c__Bacilli;o__Lactobacillales;f__Lactobacillaceae;g__Pediococcus;s__Pediococcus pentosaceus	GCF_001437285.1	99.39
MAGs.BN01_1.Bin34	d__Bacteria;p__Firmicutes;c__Bacilli;o__Lactobacillales;f__Lactobacillaceae;g__Paucilactobacillus;s__Paucilactobacillus suebicus	GCF_001434475.1	98.33

MAGs.BN01_1.Bin35	d__Bacteria;p__Firmicutes;c__Bacilli;o__Bacillales_B;f__Bacillaceae_C;g__Weizmannia;s__Weizmannia coagulans	GCF_000290615.1	98.67
MAGs.BN01_1.Bin38	d__Bacteria;p__Firmicutes;c__Bacilli;o__Lactobacillales;f__Lactobacillaceae;g__Limosilactobacillus;s__	N/A	N/A
MAGs.BN01_1.Bin41	d__Bacteria;p__Proteobacteria;c__Alphaproteobacteria;o__Acetobacterales;f__Acetobacteraceae;g__Acetobacter;s__Acetobacter syzygii	GCF_000964225.1	98.54
MAGs.BN01_1.Bin43	d__Bacteria;p__Firmicutes;c__Bacilli;o__Staphylococcales;f__Staphylococcaceae;g__Staphylococcus;s__Staphylococcus gallinarum	GCF_000875895.1	99.08
MAGs.BN01_1.Bin44	d__Bacteria;p__Firmicutes;c__Bacilli;o__Lactobacillales;f__Lactobacillaceae;g__Companilactobacillus;s__Companilactobacillus farciminis_A	GCF_014058425.1	99.63
MAGs.BN01_1.Bin50	d__Bacteria;p__Proteobacteria;c__Alphaproteobacteria;o__Acetobacterales;f__Acetobacteraceae;g__Acetobacter;s__Acetobacter orientalis	GCF_000963965.1	98.06
MAGs.BN01_1.Bin52	d__Bacteria;p__Firmicutes;c__Bacilli;o__Lactobacillales;f__Lactobacillaceae;g__Liquorilactobacillus;s__Liquorilactobacillus vini	GCF_000255495.1	98.92
MAGs.BN01_1.Bin54	d__Bacteria;p__Firmicutes;c__Bacilli;o__Lactobacillales;f__Lactobacillaceae;g__Limosilactobacillus;s__Limosilactobacillus fermentum	GCF_000159215.1	98.12
MAGs.BN01_1.Bin56	d__Bacteria;p__Firmicutes;c__Bacilli;o__Lactobacillales;f__Lactobacillaceae;g__Furfurilactobacillus;s__Furfurilactobacillus rossiae	GCF_000428925.1	98.47
MAGs.BN01_1.Bin58	d__Bacteria;p__Proteobacteria;c__Alphaproteobacteria;o__Acetobacterales;f__Acetobacteraceae;g__Acetobacter;s__Acetobacter peroxydans	GCF_006539345.1	97.81
MAGs.BN01_1.Bin59	d__Bacteria;p__Firmicutes;c__Bacilli;o__Lactobacillales;f__Lactobacillaceae;g__Weissella;s__Weissella paramesenteroides	GCF_000160575.1	98.82
MAGs.BN01_1.Bin60	d__Bacteria;p__Firmicutes;c__Bacilli;o__Lactobacillales;f__Lactobacillaceae;g__Companilactobacillus;s__	N/A	N/A
MAGs.BN01_1.Bin63	d__Bacteria;p__Actinobacteriota;c__Actinomycetia;o__Mycobacteriales;f__Pseudonocardiaceae;g__Saccharopolyspora;s__Saccharopolyspora rectivirgula	GCF_000497205.1	99.72

MAGs.BN01_1.Bin7	d__Bacteria;p__Firmicutes;c__Bacilli;o__Bacillales_B;f__Caldibacillaceae;g__Bacillus_J;s__Bacillus_J thermoamylovorans	GCF_000751775.1	98.5
MAGs.BN01_2.Bin10	d__Bacteria;p__Proteobacteria;c__Alphaproteobacteria;o__Acetobacterales;f__Acetobacteraceae;g__Acetobacter;s__Acetobacter peroxydans	GCF_006539345.1	97.8
MAGs.BN01_2.Bin16	d__Bacteria;p__Firmicutes;c__Bacilli;o__Lactobacillales;f__Lactobacillaceae;g__Limosilactobacillus;s__	N/A	N/A
MAGs.BN01_2.Bin18	d__Bacteria;p__Firmicutes;c__Bacilli;o__Lactobacillales;f__Lactobacillaceae;g__Weissella;s__Weissella paramesenteroides	GCF_000160575.1	98.84
MAGs.BN01_2.Bin21	d__Bacteria;p__Firmicutes;c__Bacilli;o__Lactobacillales;f__Lactobacillaceae;g__Limosilactobacillus;s__	N/A	N/A
MAGs.BN01_2.Bin22	d__Bacteria;p__Proteobacteria;c__Alphaproteobacteria;o__Acetobacterales;f__Acetobacteraceae;g__Acetobacter;s__Acetobacter syzygii	GCF_000964225.1	98.71
MAGs.BN01_2.Bin26	d__Bacteria;p__Firmicutes;c__Bacilli;o__Lactobacillales;f__Lactobacillaceae;g__Liquorilactobacillus;s__Liquorilactobacillus vini	GCF_000255495.1	98.73
MAGs.BN01_2.Bin27	d__Bacteria;p__Firmicutes;c__Bacilli;o__Lactobacillales;f__Lactobacillaceae;g__Liquorilactobacillus;s__Liquorilactobacillus nagelii	GCF_001434225.1	98.9
MAGs.BN01_2.Bin29	d__Bacteria;p__Proteobacteria;c__Alphaproteobacteria;o__Acetobacterales;f__Acetobacteraceae;g__Acetobacter;s__Acetobacter orientalis	GCF_000963965.1	98.03
MAGs.BN01_2.Bin31	d__Bacteria;p__Actinobacteriota;c__Actinomycetia;o__Streptomycetales;f__Streptomycetaceae;g__Streptomyces;s__Streptomyces cacaoi	GCF_006539165.1	98.95
MAGs.BN01_2.Bin32	d__Bacteria;p__Actinobacteriota;c__Actinomycetia;o__Mycobacteriales;f__Pseudonocardiaceae;g__Saccharopolyspora;s__Saccharopolyspora rectivirgula	GCF_000497205.1	99.52
MAGs.BN01_2.Bin33	d__Bacteria;p__Firmicutes;c__Bacilli;o__Lactobacillales;f__Lactobacillaceae;g__Limosilactobacillus;s__Limosilactobacillus fermentum	GCF_000159215.1	98.28
MAGs.BN01_2.Bin34	d__Bacteria;p__Firmicutes;c__Bacilli;o__Bacillales_B;f__Caldibacillaceae;g__Bacillus_J;s__Bacillus_J thermoamylovorans	GCF_000751775.1	98.54

MAGs.BN01_2.Bin39	d__Bacteria;p__Firmicutes;c__Bacilli;o__Bacillales_D;f__Amphibacillaceae;g__;s__	N/A	N/A
MAGs.BN01_2.Bin4	d__Bacteria;p__Firmicutes;c__Bacilli;o__Lactobacillales;f__Lactobacillaceae;g__Paucilactobacillus;s__Paucilactobacillus suebicus	GCF_001434475.1	98.64
MAGs.BN01_2.Bin40	d__Bacteria;p__Firmicutes;c__Bacilli;o__Lactobacillales;f__Lactobacillaceae;g__Lentilactobacillus;s__Lentilactobacillus raoultii	GCF_900411375.1	96.57
MAGs.BN01_2.Bin41	d__Bacteria;p__Firmicutes;c__Bacilli;o__Lactobacillales;f__Lactobacillaceae;g__Acetilactobacillus;s__Acetilactobacillus jinshanensis	GCF_004359375.1	99.97
MAGs.BN01_2.Bin43	d__Bacteria;p__Proteobacteria;c__Gammaproteobacteria;o__Enterobacterales;f__Enterobacteriaceae;g__Serratia_B;s__Serratia_B rubidaea	GCF_901472405.1	97.9
MAGs.BN01_2.Bin51	d__Bacteria;p__Firmicutes;c__Bacilli;o__Lactobacillales;f__Lactobacillaceae;g__Lactobacillus;s__Lactobacillus acetotolerans	GCF_001436775.1	99.15
MAGs.BN01_2.Bin6	d__Bacteria;p__Firmicutes;c__Bacilli;o__Lactobacillales;f__Lactobacillaceae;g__Lactobacillus;s__Lactobacillus amylovorus	GCF_002706375.1	98.97
MAGs.BN01_2.Bin8	d__Bacteria;p__Firmicutes;c__Bacilli;o__Bacillales_B;f__Bacillaceae_C;g__Weizmannia;s__Weizmannia coagulans	GCF_000290615.1	98.63
MAGs.BN01_3.Bin1	d__Bacteria;p__Firmicutes;c__Bacilli;o__Lactobacillales;f__Lactobacillaceae;g__Acetilactobacillus;s__Acetilactobacillus jinshanensis	GCF_004359375.1	99.98
MAGs.BN01_3.Bin10	d__Bacteria;p__Firmicutes;c__Bacilli;o__Lactobacillales;f__Lactobacillaceae;g__Paucilactobacillus;s__Paucilactobacillus suebicus	GCF_001434475.1	98.35
MAGs.BN01_3.Bin11	d__Bacteria;p__Proteobacteria;c__Gammaproteobacteria;o__Enterobacterales;f__Enterobacteriaceae;g__Serratia_B;s__Serratia_B rubidaea	GCF_901472405.1	98.13
MAGs.BN01_3.Bin12	d__Bacteria;p__Firmicutes;c__Bacilli;o__Lactobacillales;f__Lactobacillaceae;g__Lacticaseibacillus;s__Lacticaseibacillus paracasei	GCF_000829035.1	98.05
MAGs.BN01_3.Bin13	d__Bacteria;p__Actinobacteriota;c__Actinomycetia;o__Streptomycetales;f__Streptomycetaceae;g__Streptomyces;s__Streptomyces cacaoi	GCF_006539165.1	99.14
MAGs.BN01_3.Bin15	d__Bacteria;p__Firmicutes;c__Bacilli;o__Lactobacillales;f__Lactobacillaceae;g__Companila	N/A	N/A

	ctobacillus;s__		
MAGs.BN01_3.Bin18	d__Bacteria;p__Firmicutes;c__Bacilli;o__Lactobacillales;f__Lactobacillaceae;g__Pediococcus;s__Pediococcus pentosaceus	GCF_001437285.1	98.82
MAGs.BN01_3.Bin19	d__Bacteria;p__Firmicutes;c__Bacilli;o__Lactobacillales;f__Lactobacillaceae;g__Limosilactobacillus;s__Limosilactobacillus fermentum	GCF_000159215.1	98.23
MAGs.BN01_3.Bin20	d__Bacteria;p__Firmicutes;c__Bacilli;o__Lactobacillales;f__Lactobacillaceae;g__Liquorilactobacillus;s__Liquorilactobacillus nagelii	GCF_001434225.1	99.2
MAGs.BN01_3.Bin33	d__Bacteria;p__Proteobacteria;c__Gammaproteobacteria;o__Burkholderiales;f__Burkholderiaceae;g__Burkholderia;s__Burkholderia vietnamiensis	GCF_000959445.1	98.99
MAGs.BN01_3.Bin34	d__Bacteria;p__Proteobacteria;c__Alphaproteobacteria;o__Acetobacterales;f__Acetobacteraceae;g__Acetobacter;s__Acetobacter orientalis	GCF_000963965.1	98.12
MAGs.BN01_3.Bin36	d__Bacteria;p__Proteobacteria;c__Alphaproteobacteria;o__Acetobacterales;f__Acetobacteraceae;g__Acetobacter;s__Acetobacter pomorum	GCF_002738225.1	95.92
MAGs.BN01_3.Bin37	d__Bacteria;p__Proteobacteria;c__Alphaproteobacteria;o__Acetobacterales;f__Acetobacteraceae;g__Acetobacter;s__Acetobacter lovaniensis	GCF_014207635.1	99.53
MAGs.BN01_3.Bin4	d__Bacteria;p__Proteobacteria;c__Alphaproteobacteria;o__Acetobacterales;f__Acetobacteraceae;g__Acetobacter;s__Acetobacter indonesiensis	GCF_000963945.1	96.74
MAGs.BN01_3.Bin40	d__Bacteria;p__Proteobacteria;c__Alphaproteobacteria;o__Acetobacterales;f__Acetobacteraceae;g__Acetobacter;s__	N/A	N/A
MAGs.BN01_3.Bin41	d__Bacteria;p__Proteobacteria;c__Alphaproteobacteria;o__Rhizobiales;f__Xanthobacteraceae;g__Bradyrhizobium;s__	N/A	N/A
MAGs.BN01_3.Bin42	d__Bacteria;p__Firmicutes;c__Bacilli;o__Bacillales_B;f__Bacillaceae_C;g__Weizmannia;s__Weizmannia coagulans	GCF_000290615.1	98.47
MAGs.BN01_3.Bin45	d__Bacteria;p__Proteobacteria;c__Alphaproteobacteria;o__Acetobacterales;f__Acetobacteraceae;g__Acetobacter;s__Acetobacter syzygii	GCF_000964225.1	98.66
MAGs.BN01_3.Bin50	d__Bacteria;p__Firmicutes;c__Bacilli;o__Bacillales_D;f__Amphibacillaceae;g__;s__	N/A	N/A

MAGs.BN01_3.Bin52	d__Bacteria;p__Firmicutes;c__Bacilli;o__Lactobacillales;f__Lactobacillaceae;g__Lactobacillus;s__Lactobacillus amylovorus	GCF_002706375.1	98.81
MAGs.BN01_3.Bin55	d__Bacteria;p__Actinobacteriota;c__Actinomycetia;o__Mycobacteriales;f__Pseudonocardiaceae;g__Saccharopolyspora;s__Saccharopolyspora rectivirgula	GCF_000497205.1	99.75
MAGs.BN01_3.Bin6	d__Bacteria;p__Bacteroidota;c__Bacteroidia;o__Chitinophagales;f__Chitinophagaceae;g__Sediminibacterium;s__Sediminibacterium magnilacihabitans	GCA_002973575.1	98.12
MAGs.BN01_3.Bin64	d__Bacteria;p__Firmicutes;c__Bacilli;o__Lactobacillales;f__Lactobacillaceae;g__Lactiplantibacillus;s__Lactiplantibacillus plantarum	GCF_014131735.1	96.25
MAGs.BN01_3.Bin65	d__Bacteria;p__Firmicutes;c__Bacilli;o__Lactobacillales;f__Lactobacillaceae;g__Weissella;s__Weissella paramesenteroides	GCF_000160575.1	98.73
MAGs.BN01_3.Bin66	d__Bacteria;p__Firmicutes;c__Bacilli;o__Lactobacillales;f__Lactobacillaceae;g__Liquorilactobacillus;s__Liquorilactobacillus vini	GCF_000255495.1	98.86
MAGs.BN01_3.Bin8	d__Bacteria;p__Proteobacteria;c__Alphaproteobacteria;o__Acetobacterales;f__Acetobacteraceae;g__Acetobacter;s__Acetobacter peroxydans	GCF_006539345.1	97.49
MAGs.BN05_1.Bin14	d__Bacteria;p__Proteobacteria;c__Gammaproteobacteria;o__Pseudomonadales;f__Pseudomonadaceae;g__Pseudomonas_D;s__Pseudomonas_D jilinensis	GCF_003586265.1	96.35
MAGs.BN05_1.Bin16	d__Bacteria;p__Firmicutes;c__Bacilli;o__Lactobacillales;f__Lactobacillaceae;g__Limosilactobacillus;s__	N/A	N/A
MAGs.BN05_1.Bin18	d__Bacteria;p__Firmicutes;c__Bacilli;o__Bacillales_B;f__Bacillaceae_C;g__Weizmannia;s__Weizmannia coagulans	GCF_000290615.1	98.58
MAGs.BN05_1.Bin19	d__Bacteria;p__Firmicutes;c__Bacilli;o__Lactobacillales;f__Lactobacillaceae;g__Limosilactobacillus;s__	N/A	N/A
MAGs.BN05_1.Bin20	d__Bacteria;p__Firmicutes;c__Bacilli;o__Lactobacillales;f__Lactobacillaceae;g__Lactobacillus;s__Lactobacillus amylovorus	GCF_002706375.1	98.69
MAGs.BN05_1.Bin22	d__Bacteria;p__Firmicutes;c__Bacilli;o__Lactobacillales;f__Lactobacillaceae;g__Liquorilactobacillus;s__Liquorilactobacillus nagelii	GCF_001434225.1	98.66

MAGs.BN05_1.Bin24	d__Bacteria;p__Firmicutes;c__Bacilli;o__Lactobacillales;f__Lactobacillaceae;g__Limosilactobacillus;s__Limosilactobacillus pontis_A	GCF_002940945.1	97.17
MAGs.BN05_1.Bin26	d__Bacteria;p__Firmicutes;c__Bacilli;o__Lactobacillales;f__Lactobacillaceae;g__Limosilactobacillus;s__Limosilactobacillus pontis	GCF_001435345.1	98.86
MAGs.BN05_1.Bin27	d__Bacteria;p__Firmicutes;c__Bacilli;o__Lactobacillales;f__Lactobacillaceae;g__Limosilactobacillus;s__Limosilactobacillus mucosae	GCF_001436025.1	97.11
MAGs.BN05_1.Bin29	d__Bacteria;p__Firmicutes;c__Bacilli;o__Lactobacillales;f__Lactobacillaceae;g__Limosilactobacillus;s__Limosilactobacillus fermentum	GCF_000159215.1	98.27
MAGs.BN05_1.Bin31	d__Bacteria;p__Proteobacteria;c__Alphaproteobacteria;o__Acetobacterales;f__Acetobacteraceae;g__Acetobacter;s__Acetobacter peroxydans	GCF_006539345.1	97.77
MAGs.BN05_1.Bin32	d__Bacteria;p__Firmicutes;c__Bacilli;o__Lactobacillales;f__Lactobacillaceae;g__Limosilactobacillus;s__	N/A	N/A
MAGs.BN05_1.Bin33	d__Bacteria;p__Firmicutes;c__Bacilli;o__Bacillales_D;f__Amphibacillaceae;g__;s__	N/A	N/A
MAGs.BN05_1.Bin35	d__Bacteria;p__Proteobacteria;c__Alphaproteobacteria;o__Acetobacterales;f__Acetobacteraceae;g__Acetobacter;s__Acetobacter pomorum	GCF_002738225.1	95.68
MAGs.BN05_1.Bin36	d__Bacteria;p__Firmicutes;c__Bacilli;o__Lactobacillales;f__Lactobacillaceae;g__Lactobacillus;s__Lactobacillus acetotolerans	GCF_001436775.1	99.11
MAGs.BN05_1.Bin39	d__Bacteria;p__Firmicutes;c__Bacilli;o__Lactobacillales;f__Lactobacillaceae;g__Limosilactobacillus;s__	N/A	N/A
MAGs.BN05_1.Bin4	d__Bacteria;p__Firmicutes;c__Bacilli;o__Lactobacillales;f__Lactobacillaceae;g__Lactobacillus;s__	N/A	N/A
MAGs.BN05_1.Bin41	d__Bacteria;p__Firmicutes;c__Bacilli;o__Lactobacillales;f__Lactobacillaceae;g__Limosilactobacillus;s__	N/A	N/A
MAGs.BN05_1.Bin44	d__Bacteria;p__Firmicutes;c__Bacilli;o__Lactobacillales;f__Lactobacillaceae;g__Lactobacillus;s__	N/A	N/A
MAGs.BN05_1.Bin45	d__Bacteria;p__Firmicutes;c__Bacilli;o__Bacillales_B;f__Caldibacillaceae;g__Bacillus_J;s_	GCF_000751775.1	98.6

	_Bacillus_J thermoamylovorans		
MAGs.BN05_1.Bin46	d__Bacteria;p__Firmicutes;c__Bacilli;o__Lactobacillales;f__Lactobacillaceae;g__Acetilactobacillus;s__Acetilactobacillus jinshanensis	GCF_004359375.1	99.97
MAGs.BN05_1.Bin8	d__Bacteria;p__Firmicutes;c__Bacilli;o__Lactobacillales;f__Lactobacillaceae;g__Liquorilactobacillus;s__Liquorilactobacillus vini	GCF_000255495.1	98.84
MAGs.BN05_2.Bin11	d__Bacteria;p__Firmicutes;c__Bacilli;o__Bacillales_B;f__Bacillaceae_C;g__Weizmannia;s__Weizmannia coagulans	GCF_000290615.1	98.5
MAGs.BN05_2.Bin12	d__Bacteria;p__Firmicutes;c__Bacilli;o__Lactobacillales;f__Lactobacillaceae;g__Limosilactobacillus;s__	N/A	N/A
MAGs.BN05_2.Bin18	d__Bacteria;p__Firmicutes;c__Bacilli;o__Lactobacillales;f__Lactobacillaceae;g__Lactobacillus;s__Lactobacillus amylovorus	GCF_002706375.1	98.95
MAGs.BN05_2.Bin22	d__Bacteria;p__Firmicutes;c__Bacilli;o__Lactobacillales;f__Lactobacillaceae;g__Liquorilactobacillus;s__Liquorilactobacillus nagelii	GCF_001434225.1	98.87
MAGs.BN05_2.Bin27	d__Bacteria;p__Proteobacteria;c__Alphaproteobacteria;o__Acetobacterales;f__Acetobacteraceae;g__Acetobacter;s__Acetobacter pomorum	GCF_002738225.1	95.67
MAGs.BN05_2.Bin3	d__Bacteria;p__Firmicutes;c__Bacilli;o__Lactobacillales;f__Lactobacillaceae;g__Lactobacillus;s__	N/A	N/A
MAGs.BN05_2.Bin30	d__Bacteria;p__Firmicutes;c__Bacilli;o__Lactobacillales;f__Lactobacillaceae;g__Acetilactobacillus;s__Acetilactobacillus jinshanensis	GCF_004359375.1	99.97
MAGs.BN05_2.Bin31	d__Bacteria;p__Firmicutes;c__Bacilli;o__Lactobacillales;f__Lactobacillaceae;g__Liquorilactobacillus;s__Liquorilactobacillus vini	GCF_000255495.1	98.85
MAGs.BN05_2.Bin32	d__Bacteria;p__Firmicutes;c__Bacilli;o__Lactobacillales;f__Lactobacillaceae;g__Furfurilactobacillus;s__Furfurilactobacillus rossiae	GCF_000428925.1	97.99
MAGs.BN05_2.Bin37	d__Bacteria;p__Firmicutes;c__Bacilli;o__Lactobacillales;f__Lactobacillaceae;g__Limosilactobacillus;s__	N/A	N/A
MAGs.BN05_2.Bin40	d__Bacteria;p__Proteobacteria;c__Alphaproteobacteria;o__Acetobacterales;f__Acetobacterac	GCF_006539345.1	97.67

	eae;g__Acetobacter;s__Acetobacter peroxydans		
MAGs.BN05_2.Bin41	d__Bacteria;p__Firmicutes;c__Bacilli;o__Lactobacillales;f__Lactobacillaceae;g__Limosilactobacillus;s__	N/A	N/A
MAGs.BN05_2.Bin44	d__Bacteria;p__Firmicutes;c__Bacilli;o__Lactobacillales;f__Lactobacillaceae;g__Paucilactobacillus;s__Paucilactobacillus suebicus	GCF_001434475.1	98.41
MAGs.BN05_2.Bin46	d__Bacteria;p__Firmicutes;c__Bacilli;o__Lactobacillales;f__Lactobacillaceae;g__Lactobacillus;s__Lactobacillus acetotolerans	GCF_001436775.1	99.19
MAGs.BN05_2.Bin7	d__Bacteria;p__Firmicutes;c__Bacilli;o__Lactobacillales;f__Lactobacillaceae;g__Limosilactobacillus;s__Limosilactobacillus sp002299975	GCA_002299975.1	96.05
MAGs.BN05_2.Bin8	d__Bacteria;p__Firmicutes;c__Bacilli;o__Lactobacillales;f__Lactobacillaceae;g__Limosilactobacillus;s__Limosilactobacillus fermentum	GCF_000159215.1	98.27
MAGs.BN05_2.Bin9	d__Bacteria;p__Firmicutes;c__Bacilli;o__Lactobacillales;f__Lactobacillaceae;g__Limosilactobacillus;s__Limosilactobacillus mucosae	GCF_001436025.1	96.94
MAGs.BN05_3.Bin10	d__Bacteria;p__Firmicutes;c__Bacilli;o__Lactobacillales;f__Lactobacillaceae;g__Lactobacillus;s__Lactobacillus amylovorus	GCF_002706375.1	98.72
MAGs.BN05_3.Bin12	d__Bacteria;p__Firmicutes;c__Bacilli;o__Lactobacillales;f__Lactobacillaceae;g__Limosilactobacillus;s__	N/A	N/A
MAGs.BN05_3.Bin19	d__Bacteria;p__Proteobacteria;c__Alphaproteobacteria;o__Acetobacterales;f__Acetobacteraceae;g__Acetobacter;s__Acetobacter peroxydans	GCF_006539345.1	97.67
MAGs.BN05_3.Bin23	d__Bacteria;p__Firmicutes;c__Bacilli;o__Bacillales_D;f__Amphibacillaceae;g__;s__	N/A	N/A
MAGs.BN05_3.Bin24	d__Bacteria;p__Proteobacteria;c__Alphaproteobacteria;o__Acetobacterales;f__Acetobacteraceae;g__Acetobacter;s__Acetobacter pomorum	GCF_002738225.1	95.76
MAGs.BN05_3.Bin26	d__Bacteria;p__Proteobacteria;c__Gammaproteobacteria;o__Enterobacterales;f__Enterobacteriaceae;g__Serratia_B;s__Serratia_B rubidaea	GCF_901472405.1	97.98
MAGs.BN05_3.Bin30	d__Bacteria;p__Firmicutes;c__Bacilli;o__Lactobacillales;f__Lactobacillaceae;g__Paucilactobacillus;s__Paucilactobacillus suebicus	GCF_001434475.1	98.37

MAGs.BN05_3.Bin31	d__Bacteria;p__Firmicutes;c__Bacilli;o__Lactobacillales;f__Lactobacillaceae;g__Liquorilactobacillus;s__Liquorilactobacillus nagelii	GCF_001434225.1	98.67
MAGs.BN05_3.Bin34	d__Bacteria;p__Firmicutes;c__Bacilli;o__Bacillales_B;f__Caldibacillaceae;g__Bacillus_J;s__Bacillus_J thermoamylovorans	GCF_000751775.1	98.56
MAGs.BN05_3.Bin35	d__Bacteria;p__Firmicutes;c__Bacilli;o__Lactobacillales;f__Lactobacillaceae;g__Limosilactobacillus;s__	N/A	N/A
MAGs.BN05_3.Bin37	d__Bacteria;p__Firmicutes;c__Bacilli;o__Lactobacillales;f__Lactobacillaceae;g__Limosilactobacillus;s__Limosilactobacillus fermentum	GCF_000159215.1	98.11
MAGs.BN05_3.Bin40	d__Bacteria;p__Firmicutes;c__Bacilli;o__Lactobacillales;f__Lactobacillaceae;g__Limosilactobacillus;s__	N/A	N/A
MAGs.BN05_3.Bin47	d__Bacteria;p__Bacteroidota;c__Bacteroidia;o__Chitinophagales;f__Chitinophagaceae;g__Sediminibacterium;s__Sediminibacterium magnilacihabitans	GCA_002973575.1	98.25
MAGs.BN05_3.Bin49	d__Bacteria;p__Firmicutes;c__Bacilli;o__Lactobacillales;f__Lactobacillaceae;g__Liquorilactobacillus;s__Liquorilactobacillus vini	GCF_000255495.1	98.85
MAGs.BN05_3.Bin52	d__Bacteria;p__Firmicutes;c__Bacilli;o__Lactobacillales;f__Lactobacillaceae;g__Limosilactobacillus;s__Limosilactobacillus sp900557215	GCA_900557215.1	96.54
MAGs.BN05_3.Bin59	d__Bacteria;p__Firmicutes;c__Bacilli;o__Lactobacillales;f__Lactobacillaceae;g__Lactobacillus;s__Lactobacillus acetotolerans	GCF_001436775.1	99.27
MAGs.BN05_3.Bin6	d__Bacteria;p__Firmicutes;c__Bacilli;o__Lactobacillales;f__Lactobacillaceae;g__Acetilactobacillus;s__Acetilactobacillus jinshanensis	GCF_004359375.1	99.97
MAGs.BN05_3.Bin60	d__Bacteria;p__Proteobacteria;c__Alphaproteobacteria;o__Rhizobiales;f__Xanthobacteraceae;g__Bradyrhizobium;s__	N/A	N/A
MAGs.BN05_3.Bin61	d__Bacteria;p__Firmicutes;c__Bacilli;o__Bacillales_B;f__Bacillaceae_C;g__Weizmannia;s__Weizmannia coagulans	GCF_000290615.1	98.52
MAGs.BN05_3.Bin7	d__Bacteria;p__Actinobacteriota;c__Actinomycetia;o__Streptomycetales;f__Streptomycetaceae;g__Streptomyces;s__Streptomyces cacaoi	GCF_006539165.1	98.81

MAGs.BN05_3.Bin9	d__Bacteria;p__Firmicutes;c__Bacilli;o__Lactobacillales;f__Lactobacillaceae;g__Limosilactobacillus;s__Limosilactobacillus sp002299975	GCA_002299975.1	96.6
MAGs.BN07_1.Bin10	d__Bacteria;p__Proteobacteria;c__Alphaproteobacteria;o__Rhizobiales;f__Xanthobacteraceae;g__Bradyrhizobium;s__	N/A	N/A
MAGs.BN07_1.Bin13	d__Bacteria;p__Firmicutes;c__Bacilli;o__Lactobacillales;f__Lactobacillaceae;g__Acetilactobacillus;s__Acetilactobacillus jinshanensis	GCF_004359375.1	99.97
MAGs.BN07_1.Bin19	d__Bacteria;p__Firmicutes;c__Bacilli;o__Lactobacillales;f__Lactobacillaceae;g__Limosilactobacillus;s__Limosilactobacillus fermentum	GCF_000159215.1	98.23
MAGs.BN07_1.Bin2	d__Bacteria;p__Firmicutes;c__Bacilli;o__Lactobacillales;f__Lactobacillaceae;g__Limosilactobacillus;s__Limosilactobacillus mucosae	GCF_001436025.1	96.98
MAGs.BN07_1.Bin23	d__Bacteria;p__Firmicutes;c__Bacilli;o__Lactobacillales;f__Lactobacillaceae;g__Limosilactobacillus;s__Limosilactobacillus pontis	GCF_001435345.1	98.7
MAGs.BN07_1.Bin30	d__Bacteria;p__Firmicutes;c__Bacilli;o__Lactobacillales;f__Lactobacillaceae;g__Lactobacillus;s__Lactobacillus acetotolerans	GCF_001436775.1	99.3
MAGs.BN07_1.Bin31	d__Bacteria;p__Proteobacteria;c__Alphaproteobacteria;o__Acetobacterales;f__Acetobacteraceae;g__Acetobacter;s__Acetobacter peroxydans	GCF_006539345.1	97.74
MAGs.BN07_1.Bin32	d__Bacteria;p__Firmicutes;c__Bacilli;o__Lactobacillales;f__Lactobacillaceae;g__Limosilactobacillus;s__	N/A	N/A
MAGs.BN07_1.Bin33	d__Bacteria;p__Firmicutes;c__Bacilli;o__Bacillales_B;f__Caldibacillaceae;g__Bacillus_J;s__Bacillus_J thermoamylovorans	GCF_000751775.1	98.57
MAGs.BN07_1.Bin34	d__Bacteria;p__Firmicutes;c__Bacilli;o__Bacillales_D;f__Amphibacillaceae;g__;s__	N/A	N/A
MAGs.BN07_1.Bin38	d__Bacteria;p__Firmicutes;c__Bacilli;o__Lactobacillales;f__Lactobacillaceae;g__Limosilactobacillus;s__	N/A	N/A
MAGs.BN07_1.Bin39	d__Bacteria;p__Proteobacteria;c__Alphaproteobacteria;o__Acetobacterales;f__Acetobacteraceae;g__Acetobacter;s__Acetobacter pomorum	GCF_002738225.1	95.62
MAGs.BN07_1.Bin44	d__Bacteria;p__Bacteroidota;c__Bacteroidia;o__Chitinophagales;f__Chitinophagaceae;g__Se	GCA_002973575.1	98.09

	diminibacterium;s__Sediminibacterium magnilacihabitans		
MAGs.BN07_1.Bin48	d__Bacteria;p__Firmicutes;c__Bacilli;o__Lactobacillales;f__Lactobacillaceae;g__Liquorilactobacillus;s__Liquorilactobacillus nagelii	GCF_001434225.1	98.88
MAGs.BN07_1.Bin49	d__Bacteria;p__Firmicutes;c__Bacilli;o__Lactobacillales;f__Lactobacillaceae;g__Liquorilactobacillus;s__Liquorilactobacillus vini	GCF_000255495.1	98.67
MAGs.BN07_1.Bin5	d__Bacteria;p__Firmicutes;c__Bacilli;o__Bacillales_B;f__Bacillaceae_C;g__Weizmannia;s__Weizmannia coagulans	GCF_000290615.1	98.56
MAGs.BN07_1.Bin7	d__Bacteria;p__Firmicutes;c__Bacilli;o__Lactobacillales;f__Lactobacillaceae;g__Limosilactobacillus;s__	N/A	N/A
MAGs.BN07_2.Bin14	d__Bacteria;p__Firmicutes;c__Bacilli;o__Lactobacillales;f__Lactobacillaceae;g__Lactiplantibacillus;s__Lactiplantibacillus plantarum	GCF_014131735.1	97.51
MAGs.BN07_2.Bin24	d__Bacteria;p__Firmicutes;c__Bacilli;o__Lactobacillales;f__Lactobacillaceae;g__Limosilactobacillus;s__Limosilactobacillus sp002299975	GCA_002299975.1	95.98
MAGs.BN07_2.Bin27	d__Bacteria;p__Firmicutes;c__Bacilli;o__Bacillales_B;f__Bacillaceae_C;g__Weizmannia;s__Weizmannia coagulans	GCF_000290615.1	98.58
MAGs.BN07_2.Bin29	d__Bacteria;p__Firmicutes;c__Bacilli;o__Lactobacillales;f__Lactobacillaceae;g__Liquorilactobacillus;s__Liquorilactobacillus nagelii	GCF_001434225.1	98.83
MAGs.BN07_2.Bin3	d__Bacteria;p__Bacteroidota;c__Bacteroidia;o__Chitinophagales;f__Chitinophagaceae;g__Sediminibacterium;s__Sediminibacterium magnilacihabitans	GCA_002973575.1	98.34
MAGs.BN07_2.Bin30	d__Bacteria;p__Firmicutes;c__Bacilli;o__Bacillales_D;f__Amphibacillaceae;g__;s__	N/A	N/A
MAGs.BN07_2.Bin31	d__Bacteria;p__Firmicutes;c__Bacilli;o__Lactobacillales;f__Lactobacillaceae;g__Limosilactobacillus;s__	N/A	N/A
MAGs.BN07_2.Bin32	d__Bacteria;p__Firmicutes;c__Bacilli;o__Lactobacillales;f__Lactobacillaceae;g__Limosilactobacillus;s__Limosilactobacillus pontis_A	GCF_002940945.1	98.68
MAGs.BN07_2.Bin33	d__Bacteria;p__Firmicutes;c__Bacilli;o__Lactobacillales;f__Lactobacillaceae;g__Lactobacillus;s__Lactobacillus amylovorus	GCF_002706375.1	98.95

MAGs.BN07_2.Bin37	d__Bacteria;p__Firmicutes;c__Bacilli;o__Bacillales_B;f__Caldibacillaceae;g__Bacillus_J;s__Bacillus_J thermoamylovorans	GCF_000751775.1	98.75
MAGs.BN07_2.Bin40	d__Bacteria;p__Proteobacteria;c__Alphaproteobacteria;o__Acetobacterales;f__Acetobacteraceae;g__Acetobacter;s__Acetobacter peroxydans	GCF_006539345.1	97.74
MAGs.BN07_2.Bin42	d__Bacteria;p__Firmicutes;c__Bacilli;o__Lactobacillales;f__Lactobacillaceae;g__Limosilactobacillus;s__	N/A	N/A
MAGs.BN07_2.Bin43	d__Bacteria;p__Firmicutes;c__Bacilli;o__Lactobacillales;f__Lactobacillaceae;g__Lactobacillus;s__	N/A	N/A
MAGs.BN07_2.Bin5	d__Bacteria;p__Firmicutes;c__Bacilli;o__Lactobacillales;f__Lactobacillaceae;g__Acetilactobacillus;s__Acetilactobacillus jinshanensis	GCF_004359375.1	99.97
MAGs.BN07_2.Bin7	d__Bacteria;p__Proteobacteria;c__Alphaproteobacteria;o__Acetobacterales;f__Acetobacteraceae;g__Acetobacter;s__Acetobacter pomorum	GCF_002738225.1	95.64
MAGs.BN07_2.Bin8	d__Bacteria;p__Proteobacteria;c__Alphaproteobacteria;o__Acetobacterales;f__Acetobacteraceae;g__Komagataeibacter;s__Komagataeibacter melaceti	GCF_003403295.1	98.12
MAGs.BN07_3.Bin10	d__Bacteria;p__Firmicutes;c__Bacilli;o__Lactobacillales;f__Lactobacillaceae;g__Acetilactobacillus;s__Acetilactobacillus jinshanensis	GCF_004359375.1	99.97
MAGs.BN07_3.Bin12	d__Bacteria;p__Firmicutes;c__Bacilli;o__Lactobacillales;f__Lactobacillaceae;g__Limosilactobacillus;s__	N/A	N/A
MAGs.BN07_3.Bin15	d__Bacteria;p__Firmicutes;c__Bacilli;o__Lactobacillales;f__Lactobacillaceae;g__Liquorilactobacillus;s__Liquorilactobacillus vini	GCF_000255495.1	98.88
MAGs.BN07_3.Bin16	d__Bacteria;p__Firmicutes;c__Bacilli;o__Lactobacillales;f__Lactobacillaceae;g__Limosilactobacillus;s__Limosilactobacillus mucosae	GCF_001436025.1	97
MAGs.BN07_3.Bin18	d__Bacteria;p__Firmicutes;c__Bacilli;o__Lactobacillales;f__Lactobacillaceae;g__Lactobacillus;s__Lactobacillus amylovorus	GCF_002706375.1	98.95
MAGs.BN07_3.Bin22	d__Bacteria;p__Firmicutes;c__Bacilli;o__Lactobacillales;f__Lactobacillaceae;g__Liquorilactobacillus;s__Liquorilactobacillus nagelii	GCF_001434225.1	98.87

MAGs.BN07_3.Bin23	d__Bacteria;p__Proteobacteria;c__Alphaproteobacteria;o__Acetobacterales;f__Acetobacteraceae;g__Acetobacter;s__Acetobacter pomorum	GCF_002738225.1	95.79
MAGs.BN07_3.Bin25	d__Bacteria;p__Proteobacteria;c__Gammaproteobacteria;o__Enterobacterales;f__Enterobacteriaceae;g__Serratia_B;s__Serratia_B rubidaea	GCF_901472405.1	98.14
MAGs.BN07_3.Bin26	d__Bacteria;p__Firmicutes;c__Bacilli;o__Lactobacillales;f__Lactobacillaceae;g__Limosilactobacillus;s__Limosilactobacillus fermentum	GCF_000159215.1	98.23
MAGs.BN07_3.Bin32	d__Bacteria;p__Firmicutes;c__Bacilli;o__Lactobacillales;f__Lactobacillaceae;g__Lactobacillus;s__Lactobacillus acetotolerans	GCF_001436775.1	99.27
MAGs.BN07_3.Bin33	d__Bacteria;p__Firmicutes;c__Bacilli;o__Lactobacillales;f__Lactobacillaceae;g__Limosilactobacillus;s__Limosilactobacillus pontis_A	GCF_002940945.1	98.51
MAGs.BN07_3.Bin35	d__Bacteria;p__Firmicutes;c__Bacilli;o__Bacillales_D;f__Amphibacillaceae;g__;s__	N/A	N/A
MAGs.BN07_3.Bin36	d__Bacteria;p__Proteobacteria;c__Alphaproteobacteria;o__Acetobacterales;f__Acetobacteraceae;g__Acetobacter;s__Acetobacter peroxydans	GCF_006539345.1	97.73
MAGs.BN07_3.Bin38	d__Bacteria;p__Firmicutes;c__Bacilli;o__Bacillales_B;f__Bacillaceae_C;g__Weizmannia;s__Weizmannia coagulans	GCF_000290615.1	98.44
MAGs.BN07_3.Bin4	d__Bacteria;p__Firmicutes;c__Bacilli;o__Lactobacillales;f__Lactobacillaceae;g__Lactobacillus;s__	N/A	N/A
MAGs.BN07_3.Bin40	d__Bacteria;p__Firmicutes;c__Bacilli;o__Lactobacillales;f__Lactobacillaceae;g__Paucilactobacillus;s__Paucilactobacillus suebicus	GCF_001434475.1	98.33
MAGs.BN07_3.Bin8	d__Bacteria;p__Firmicutes;c__Bacilli;o__Lactobacillales;f__Lactobacillaceae;g__Furfurilactobacillus;s__Furfurilactobacillus rossiae	GCF_000428925.1	98.09
MAGs.BN09_1.Bin1	d__Bacteria;p__Firmicutes;c__Bacilli;o__Lactobacillales;f__Lactobacillaceae;g__Ligilactobacillus;s__Ligilactobacillus acidipiscis	GCF_001435755.1	97.44
MAGs.BN09_1.Bin10	d__Bacteria;p__Firmicutes;c__Bacilli;o__Lactobacillales;f__Lactobacillaceae;g__Acetilactobacillus;s__Acetilactobacillus jinshanensis	GCF_004359375.1	99.97
MAGs.BN09_1.Bin11	d__Bacteria;p__Firmicutes;c__Bacilli;o__Lactobacillales;f__Lactobacillaceae;g__Limosilact	N/A	N/A

	obacillus;s__		
MAGs.BN09_1.Bin12	d__Bacteria;p__Bacteroidota;c__Bacteroidia;o__Chitinophagales;f__Chitinophagaceae;g__Sediminibacterium;s__Sediminibacterium magnilacihabitans	GCA_002973575.1	98.12
MAGs.BN09_1.Bin13	d__Bacteria;p__Firmicutes;c__Bacilli;o__Lactobacillales;f__Lactobacillaceae;g__Limosilactobacillus;s__	N/A	N/A
MAGs.BN09_1.Bin21	d__Bacteria;p__Firmicutes;c__Bacilli;o__Lactobacillales;f__Lactobacillaceae;g__Lactiplantibacillus;s__Lactiplantibacillus plantarum	GCF_014131735.1	96.61
MAGs.BN09_1.Bin23	d__Bacteria;p__Firmicutes;c__Bacilli;o__Lactobacillales;f__Lactobacillaceae;g__Lactobacillus;s__	N/A	N/A
MAGs.BN09_1.Bin27	d__Bacteria;p__Firmicutes;c__Bacilli;o__Bacillales_B;f__Caldibacillaceae;g__Bacillus_J;s__Bacillus_J thermoamylovorans	GCF_000751775.1	98.61
MAGs.BN09_1.Bin28	d__Bacteria;p__Firmicutes;c__Bacilli;o__Lactobacillales;f__Lactobacillaceae;g__Lactobacillus;s__Lactobacillus acetotolerans	GCF_001436775.1	99.09
MAGs.BN09_1.Bin30	d__Bacteria;p__Firmicutes;c__Bacilli;o__Lactobacillales;f__Lactobacillaceae;g__Companilactobacillus;s__Companilactobacillus farciminis_A	GCF_014058425.1	99.25
MAGs.BN09_1.Bin31	d__Bacteria;p__Firmicutes;c__Bacilli;o__Lactobacillales;f__Lactobacillaceae;g__Limosilactobacillus;s__Limosilactobacillus fermentum	GCF_000159215.1	98.22
MAGs.BN09_1.Bin32	d__Bacteria;p__Bacteroidota;c__Bacteroidia;o__Bacteroidales;f__Bacteroidaceae;g__Prevotella;s__	N/A	N/A
MAGs.BN09_1.Bin35	d__Bacteria;p__Proteobacteria;c__Alphaproteobacteria;o__Rhizobiales;f__Xanthobacteraceae;g__Bradyrhizobium;s__	N/A	N/A
MAGs.BN09_1.Bin39	d__Bacteria;p__Firmicutes;c__Bacilli;o__Bacillales_D;f__Amphibacillaceae;g__;s__	N/A	N/A
MAGs.BN09_1.Bin48	d__Bacteria;p__Firmicutes;c__Bacilli;o__Bacillales_B;f__Bacillaceae_C;g__Weizmannia;s__Weizmannia coagulans	GCF_000290615.1	98.53
MAGs.BN09_1.Bin52	d__Bacteria;p__Firmicutes;c__Bacilli;o__Lactobacillales;f__Lactobacillaceae;g__Lactobacillus;s__Lactobacillus amylovorus	GCF_002706375.1	98.88

MAGs.BN09_1.Bin53	d__Bacteria;p__Firmicutes;c__Bacilli;o__Lactobacillales;f__Lactobacillaceae;g__Companilactobacillus;s__	N/A	N/A
MAGs.BN09_1.Bin6	d__Bacteria;p__Firmicutes;c__Bacilli;o__Lactobacillales;f__Lactobacillaceae;g__Lactobacillus;s__	N/A	N/A
MAGs.BN09_1.Bin7	d__Bacteria;p__Firmicutes;c__Bacilli;o__Lactobacillales;f__Lactobacillaceae;g__Limosilactobacillus;s__Limosilactobacillus sp900557215	GCA_900557215.1	96.78
MAGs.BN09_1.Bin8	d__Bacteria;p__Firmicutes;c__Bacilli;o__Lactobacillales;f__Lactobacillaceae;g__Limosilactobacillus;s__	N/A	N/A
MAGs.BN09_2.Bin11	d__Bacteria;p__Firmicutes;c__Bacilli;o__Lactobacillales;f__Lactobacillaceae;g__Companilactobacillus;s__Companilactobacillus farciminis_A	GCF_014058425.1	99.14
MAGs.BN09_2.Bin12	d__Bacteria;p__Firmicutes;c__Bacilli;o__Lactobacillales;f__Lactobacillaceae;g__Paucilactobacillus;s__Paucilactobacillus suebicus	GCF_001434475.1	98.37
MAGs.BN09_2.Bin16	d__Bacteria;p__Proteobacteria;c__Alphaproteobacteria;o__Acetobacterales;f__Acetobacteraceae;g__Acetobacter;s__Acetobacter ghanensis	GCF_001499675.1	98.42
MAGs.BN09_2.Bin2	d__Bacteria;p__Proteobacteria;c__Alphaproteobacteria;o__Rhizobiales;f__Xanthobacteraceae;g__Bradyrhizobium;s__	N/A	N/A
MAGs.BN09_2.Bin20	d__Bacteria;p__Firmicutes;c__Bacilli;o__Lactobacillales;f__Lactobacillaceae;g__Limosilactobacillus;s__	N/A	N/A
MAGs.BN09_2.Bin21	d__Bacteria;p__Actinobacteriota;c__Actinomycetia;o__Actinomycetales;f__Bifidobacteriaceae;g__Bifidobacterium;s__Bifidobacterium thermophilum	GCF_000771265.1	95.44
MAGs.BN09_2.Bin23	d__Bacteria;p__Firmicutes;c__Bacilli;o__Lactobacillales;f__Lactobacillaceae;g__Limosilactobacillus;s__	N/A	N/A
MAGs.BN09_2.Bin24	d__Bacteria;p__Firmicutes;c__Bacilli;o__Lactobacillales;f__Lactobacillaceae;g__Lactiplantibacillus;s__Lactiplantibacillus plantarum	GCF_014131735.1	96.38
MAGs.BN09_2.Bin3	d__Bacteria;p__Firmicutes;c__Bacilli;o__Lactobacillales;f__Lactobacillaceae;g__Lactobacillus;s__Lactobacillus amylovorus	GCF_002706375.1	99.01

MAGs.BN09_2.Bin30	d__Bacteria;p__Bacteroidota;c__Bacteroidia;o__Chitinophagales;f__Chitinophagaceae;g__Sediminibacterium;s__Sediminibacterium magnilacihabitans	GCA_002973575.1	98.12
MAGs.BN09_2.Bin31	d__Bacteria;p__Firmicutes;c__Bacilli;o__Lactobacillales;f__Lactobacillaceae;g__Ligilactobacillus;s__Ligilactobacillus acidipiscis	GCF_001435755.1	97.44
MAGs.BN09_2.Bin33	d__Bacteria;p__Firmicutes;c__Bacilli;o__Lactobacillales;f__Lactobacillaceae;g__Companilactobacillus;s__	N/A	N/A
MAGs.BN09_2.Bin34	d__Bacteria;p__Firmicutes;c__Bacilli;o__Lactobacillales;f__Lactobacillaceae;g__Limosilactobacillus;s__	N/A	N/A
MAGs.BN09_2.Bin39	d__Bacteria;p__Firmicutes;c__Bacilli;o__Lactobacillales;f__Lactobacillaceae;g__Limosilactobacillus;s__Limosilactobacillus fermentum	GCF_000159215.1	98.16
MAGs.BN09_2.Bin43	d__Bacteria;p__Firmicutes;c__Bacilli;o__Lactobacillales;f__Lactobacillaceae;g__Lactobacillus;s__	N/A	N/A
MAGs.BN09_2.Bin46	d__Bacteria;p__Firmicutes;c__Bacilli;o__Lactobacillales;f__Lactobacillaceae;g__Acetilactobacillus;s__Acetilactobacillus jinshanensis	GCF_004359375.1	99.98
MAGs.BN09_2.Bin48	d__Bacteria;p__Firmicutes;c__Bacilli;o__Lactobacillales;f__Lactobacillaceae;g__Lactobacillus;s__	N/A	N/A
MAGs.BN09_2.Bin7	d__Bacteria;p__Firmicutes;c__Bacilli;o__Lactobacillales;f__Lactobacillaceae;g__Lactobacillus;s__Lactobacillus acetotolerans	GCF_001436775.1	99.02
MAGs.BN09_3.Bin1	d__Bacteria;p__Firmicutes;c__Bacilli;o__Lactobacillales;f__Lactobacillaceae;g__Limosilactobacillus;s__Limosilactobacillus fermentum	GCF_000159215.1	98.25
MAGs.BN09_3.Bin10	d__Bacteria;p__Firmicutes;c__Bacilli;o__Lactobacillales;f__Lactobacillaceae;g__Liquorilactobacillus;s__Liquorilactobacillus vini	GCF_000255495.1	98.88
MAGs.BN09_3.Bin12	d__Bacteria;p__Firmicutes;c__Bacilli;o__Lactobacillales;f__Lactobacillaceae;g__Limosilactobacillus;s__	N/A	N/A
MAGs.BN09_3.Bin15	d__Bacteria;p__Firmicutes;c__Bacilli;o__Lactobacillales;f__Lactobacillaceae;g__Lactobacillus;s__Lactobacillus acetotolerans	GCF_001436775.1	99.23

MAGs.BN09_3.Bin16	d__Bacteria;p__Firmicutes;c__Bacilli;o__Lactobacillales;f__Lactobacillaceae;g__Limosilactobacillus;s__	N/A	N/A
MAGs.BN09_3.Bin17	d__Bacteria;p__Proteobacteria;c__Alphaproteobacteria;o__Rhizobiales;f__Xanthobacteraceae;g__Bradyrhizobium;s__	N/A	N/A
MAGs.BN09_3.Bin19	d__Bacteria;p__Firmicutes;c__Bacilli;o__Lactobacillales;f__Lactobacillaceae;g__Ligilactobacillus;s__Ligilactobacillus acidipiscis	GCF_001435755.1	97.61
MAGs.BN09_3.Bin27	d__Bacteria;p__Firmicutes;c__Bacilli;o__Lactobacillales;f__Lactobacillaceae;g__Lactobacillus;s__	N/A	N/A
MAGs.BN09_3.Bin28	d__Bacteria;p__Firmicutes;c__Bacilli;o__Lactobacillales;f__Lactobacillaceae;g__Liquorilactobacillus;s__Liquorilactobacillus nagelii	GCF_001434225.1	98.78
MAGs.BN09_3.Bin32	d__Bacteria;p__Firmicutes;c__Bacilli;o__Lactobacillales;f__Lactobacillaceae;g__Limosilactobacillus;s__	N/A	N/A
MAGs.BN09_3.Bin33	d__Bacteria;p__Firmicutes;c__Bacilli;o__Lactobacillales;f__Lactobacillaceae;g__Lactobacillus;s__Lactobacillus amylovorus	GCF_002706375.1	98.92
MAGs.BN09_3.Bin43	d__Bacteria;p__Firmicutes;c__Bacilli;o__Lactobacillales;f__Lactobacillaceae;g__Limosilactobacillus;s__	N/A	N/A
MAGs.BN09_3.Bin44	d__Bacteria;p__Firmicutes;c__Bacilli;o__Lactobacillales;f__Lactobacillaceae;g__Limosilactobacillus;s__	N/A	N/A
MAGs.BN09_3.Bin45	d__Bacteria;p__Firmicutes;c__Bacilli;o__Lactobacillales;f__Lactobacillaceae;g__Lacticaseibacillus;s__Lacticaseibacillus paracasei	GCF_000829035.1	98.04
MAGs.BN09_3.Bin46	d__Bacteria;p__Firmicutes;c__Bacilli;o__Lactobacillales;f__Lactobacillaceae;g__Lactobacillus;s__	N/A	N/A
MAGs.BN09_3.Bin48	d__Bacteria;p__Bacteroidota;c__Bacteroidia;o__Chitinophagales;f__Chitinophagaceae;g__Sediminibacterium;s__Sediminibacterium magnilacihabitans	GCA_002973575.1	98.12
MAGs.BN09_3.Bin5	d__Bacteria;p__Firmicutes;c__Bacilli;o__Lactobacillales;f__Lactobacillaceae;g__Limosilactobacillus;s__	N/A	N/A

MAGs.BN09_3.Bin6	d__Bacteria;p__Firmicutes;c__Bacilli;o__Lactobacillales;f__Lactobacillaceae;g__Acetilactobacillus;s__Acetilactobacillus jinshanensis	GCF_004359375.1	99.98
MAGs.BN09_3.Bin7	d__Bacteria;p__Proteobacteria;c__Alphaproteobacteria;o__Acetobacterales;f__Acetobacteraceae;g__Acetobacter;s__Acetobacter ghanensis	GCF_001499675.1	98.42
MAGs.BN09_3.Bin8	d__Bacteria;p__Firmicutes;c__Bacilli;o__Lactobacillales;f__Lactobacillaceae;g__Companilactobacillus;s__	N/A	N/A
MAGs.BN09_3.Bin9	d__Bacteria;p__Firmicutes;c__Bacilli;o__Lactobacillales;f__Lactobacillaceae;g__Companilactobacillus;s__	N/A	N/A
MAGs.BN11_1.Bin1	d__Bacteria;p__Firmicutes;c__Bacilli;o__Lactobacillales;f__Lactobacillaceae;g__Lactobacillus;s__	N/A	N/A
MAGs.BN11_1.Bin11	d__Bacteria;p__Proteobacteria;c__Alphaproteobacteria;o__Rhizobiales;f__Xanthobacteraceae;g__Bradyrhizobium;s__	N/A	N/A
MAGs.BN11_1.Bin14	d__Bacteria;p__Firmicutes;c__Bacilli;o__Lactobacillales;f__Lactobacillaceae;g__Furfurilactobacillus;s__Furfurilactobacillus rossiae	GCF_000428925.1	98.17
MAGs.BN11_1.Bin15	d__Bacteria;p__Firmicutes;c__Bacilli;o__Lactobacillales;f__Lactobacillaceae;g__Limosilactobacillus;s__	N/A	N/A
MAGs.BN11_1.Bin16	d__Bacteria;p__Firmicutes;c__Bacilli;o__Lactobacillales;f__Lactobacillaceae;g__Limosilactobacillus;s__Limosilactobacillus pontis	GCF_001435345.1	98.66
MAGs.BN11_1.Bin17	d__Bacteria;p__Firmicutes;c__Bacilli;o__Lactobacillales;f__Lactobacillaceae;g__Lactobacillus;s__Lactobacillus amylovorus	GCF_002706375.1	98.96
MAGs.BN11_1.Bin18	d__Bacteria;p__Firmicutes;c__Bacilli;o__Lactobacillales;f__Lactobacillaceae;g__Limosilactobacillus;s__Limosilactobacillus fermentum	GCF_000159215.1	98.28
MAGs.BN11_1.Bin19	d__Bacteria;p__Proteobacteria;c__Alphaproteobacteria;o__Acetobacterales;f__Acetobacteraceae;g__Acetobacter;s__Acetobacter pomorum	GCF_002738225.1	95.73
MAGs.BN11_1.Bin20	d__Bacteria;p__Proteobacteria;c__Alphaproteobacteria;o__Acetobacterales;f__Acetobacteraceae;g__Acetobacter;s__Acetobacter peroxydans	GCF_006539345.1	97.44

MAGs.BN11_1.Bin24	d__Bacteria;p__Firmicutes;c__Bacilli;o__Lactobacillales;f__Lactobacillaceae;g__Liquorilactobacillus;s__Liquorilactobacillus nagelii	GCF_001434225.1	98.59
MAGs.BN11_1.Bin27	d__Bacteria;p__Firmicutes;c__Bacilli;o__Lactobacillales;f__Lactobacillaceae;g__Lactobacillus;s__Lactobacillus acetotolerans	GCF_001436775.1	99
MAGs.BN11_1.Bin28	d__Bacteria;p__Firmicutes;c__Bacilli;o__Lactobacillales;f__Lactobacillaceae;g__Limosilactobacillus;s__	N/A	N/A
MAGs.BN11_1.Bin32	d__Bacteria;p__Firmicutes;c__Bacilli;o__Bacillales_D;f__Amphibacillaceae;g__;s__	N/A	N/A
MAGs.BN11_1.Bin33	d__Bacteria;p__Firmicutes;c__Bacilli;o__Lactobacillales;f__Lactobacillaceae;g__Pediococcus;s__Pediococcus acidilactici	GCF_000146325.1	98.8
MAGs.BN11_1.Bin36	d__Bacteria;p__Firmicutes;c__Bacilli;o__Bacillales_B;f__Bacillaceae_C;g__Weizmannia;s__Weizmannia coagulans	GCF_000290615.1	98.58
MAGs.BN11_1.Bin37	d__Bacteria;p__Bacteroidota;c__Bacteroidia;o__Chitinophagales;f__Chitinophagaceae;g__Sediminibacterium;s__Sediminibacterium magnilacihabitans	GCA_002973575.1	98.4
MAGs.BN11_1.Bin5	d__Bacteria;p__Firmicutes;c__Bacilli;o__Lactobacillales;f__Lactobacillaceae;g__Liquorilactobacillus;s__Liquorilactobacillus vini	GCF_000255495.1	98.84
MAGs.BN11_1.Bin6	d__Bacteria;p__Firmicutes;c__Bacilli;o__Lactobacillales;f__Lactobacillaceae;g__Limosilactobacillus;s__Limosilactobacillus pontis_A	GCF_002940945.1	98.42
MAGs.BN11_1.Bin9	d__Bacteria;p__Firmicutes;c__Bacilli;o__Lactobacillales;f__Lactobacillaceae;g__Acetilactobacillus;s__Acetilactobacillus jinshanensis	GCF_004359375.1	99.97
MAGs.BN11_2.Bin1	d__Bacteria;p__Firmicutes;c__Bacilli;o__Lactobacillales;f__Lactobacillaceae;g__Lactiplantibacillus;s__Lactiplantibacillus plantarum	GCF_014131735.1	97.84
MAGs.BN11_2.Bin15	d__Bacteria;p__Firmicutes;c__Bacilli;o__Lactobacillales;f__Lactobacillaceae;g__Pediococcus;s__Pediococcus acidilactici	GCF_000146325.1	99.08
MAGs.BN11_2.Bin2	d__Bacteria;p__Bacteroidota;c__Bacteroidia;o__Chitinophagales;f__Chitinophagaceae;g__Sediminibacterium;s__Sediminibacterium magnilacihabitans	GCA_002973575.1	98.11
MAGs.BN11_2.Bin20	d__Bacteria;p__Firmicutes;c__Bacilli;o__Bacillales_D;f__Amphibacillaceae;g__;s__	N/A	N/A

MAGs.BN11_2.Bin21	d__Bacteria;p__Proteobacteria;c__Alphaproteobacteria;o__Acetobacterales;f__Acetobacteraceae;g__Acetobacter;s__Acetobacter pomorum	GCF_002738225.1	95.6
MAGs.BN11_2.Bin22	d__Bacteria;p__Firmicutes;c__Bacilli;o__Lactobacillales;f__Lactobacillaceae;g__Limosilactobacillus;s__	N/A	N/A
MAGs.BN11_2.Bin23	d__Bacteria;p__Firmicutes;c__Bacilli;o__Bacillales_B;f__Bacillaceae_C;g__Weizmannia;s__Weizmannia coagulans	GCF_000290615.1	98.51
MAGs.BN11_2.Bin25	d__Bacteria;p__Firmicutes;c__Bacilli;o__Lactobacillales;f__Lactobacillaceae;g__Lactobacillus;s__Lactobacillus acetotolerans	GCF_001436775.1	99.32
MAGs.BN11_2.Bin26	d__Bacteria;p__Firmicutes;c__Bacilli;o__Bacillales_B;f__Caldibacillaceae;g__Bacillus_J;s__Bacillus_J thermoamylovorans	GCF_000751775.1	98.63
MAGs.BN11_2.Bin27	d__Bacteria;p__Proteobacteria;c__Alphaproteobacteria;o__Acetobacterales;f__Acetobacteraceae;g__Acetobacter;s__Acetobacter peroxydans	GCF_006539345.1	97.69
MAGs.BN11_2.Bin28	d__Bacteria;p__Firmicutes;c__Bacilli;o__Lactobacillales;f__Lactobacillaceae;g__Limosilactobacillus;s__	N/A	N/A
MAGs.BN11_2.Bin29	d__Bacteria;p__Proteobacteria;c__Alphaproteobacteria;o__Rhizobiales;f__Xanthobacteraceae;g__Bradyrhizobium;s__	N/A	N/A
MAGs.BN11_2.Bin3	d__Bacteria;p__Firmicutes;c__Bacilli;o__Lactobacillales;f__Lactobacillaceae;g__Limosilactobacillus;s__Limosilactobacillus fermentum	GCF_000159215.1	98.14
MAGs.BN11_2.Bin32	d__Bacteria;p__Proteobacteria;c__Alphaproteobacteria;o__Acetobacterales;f__Acetobacteraceae;g__Komagataeibacter;s__Komagataeibacter melaceti	GCF_003403295.1	97.93
MAGs.BN11_2.Bin33	d__Bacteria;p__Firmicutes;c__Bacilli;o__Lactobacillales;f__Lactobacillaceae;g__Limosilactobacillus;s__Limosilactobacillus pontis_A	GCF_002940945.1	98.61
MAGs.BN11_2.Bin39	d__Bacteria;p__Firmicutes;c__Bacilli;o__Lactobacillales;f__Lactobacillaceae;g__Limosilactobacillus;s__	N/A	N/A
MAGs.BN11_2.Bin7	d__Bacteria;p__Firmicutes;c__Bacilli;o__Lactobacillales;f__Lactobacillaceae;g__Acetilactobacillus;s__Acetilactobacillus jinshanensis	GCF_004359375.1	99.97

MAGs.BN11_2.Bin9	d__Bacteria;p__Firmicutes;c__Bacilli;o__Lactobacillales;f__Lactobacillaceae;g__Lactobacillus;s__Lactobacillus amylovorus	GCF_002706375.1	98.87
MAGs.BN11_3.Bin1	d__Bacteria;p__Firmicutes;c__Bacilli;o__Lactobacillales;f__Lactobacillaceae;g__Limosilactobacillus;s__	N/A	N/A
MAGs.BN11_3.Bin10	d__Bacteria;p__Firmicutes;c__Bacilli;o__Bacillales_A;f__Planococcaceae;g__Rummeliibacillus;s__Rummeliibacillus sp003576525	GCF_003576525.1	99.3
MAGs.BN11_3.Bin13	d__Bacteria;p__Proteobacteria;c__Alphaproteobacteria;o__Acetobacterales;f__Acetobacteraceae;g__Acetobacter;s__Acetobacter peroxydans	GCF_006539345.1	97.81
MAGs.BN11_3.Bin14	d__Bacteria;p__Firmicutes;c__Bacilli;o__Lactobacillales;f__Lactobacillaceae;g__Limosilactobacillus;s__	N/A	N/A
MAGs.BN11_3.Bin19	d__Bacteria;p__Firmicutes;c__Bacilli;o__Lactobacillales;f__Lactobacillaceae;g__Limosilactobacillus;s__Limosilactobacillus pontis_A	GCF_002940945.1	98.86
MAGs.BN11_3.Bin2	d__Bacteria;p__Firmicutes;c__Bacilli;o__Lactobacillales;f__Lactobacillaceae;g__Furfurilactobacillus;s__Furfurilactobacillus rossiae	GCF_000428925.1	98.01
MAGs.BN11_3.Bin26	d__Bacteria;p__Firmicutes;c__Bacilli;o__Bacillales_B;f__Bacillaceae_C;g__Weizmannia;s__Weizmannia coagulans	GCF_000290615.1	98.41
MAGs.BN11_3.Bin27	d__Bacteria;p__Firmicutes;c__Bacilli;o__Paenibacillales;f__Paenibacillaceae;g__Paenibacillus;s__Paenibacillus sp000787385	GCF_000787385.1	98.89
MAGs.BN11_3.Bin3	d__Bacteria;p__Firmicutes;c__Bacilli;o__Lactobacillales;f__Lactobacillaceae;g__Lactobacillus;s__	N/A	N/A
MAGs.BN11_3.Bin31	d__Bacteria;p__Firmicutes;c__Bacilli;o__Bacillales_D;f__Amphibacillaceae;g__;s__	N/A	N/A
MAGs.BN11_3.Bin32	d__Bacteria;p__Proteobacteria;c__Alphaproteobacteria;o__Acetobacterales;f__Acetobacteraceae;g__Acetobacter;s__Acetobacter pomorum	GCF_002738225.1	95.65
MAGs.BN11_3.Bin35	d__Bacteria;p__Firmicutes;c__Bacilli;o__Lactobacillales;f__Lactobacillaceae;g__Limosilactobacillus;s__Limosilactobacillus fermentum	GCF_000159215.1	98.16
MAGs.BN11_3.Bin36	d__Bacteria;p__Firmicutes;c__Bacilli;o__Lactobacillales;f__Lactobacillaceae;g__Acetilactob	GCF_004359375.1	99.97

	acillus;s__Acetilactobacillus jinshanensis		
MAGs.BN11_3.Bin37	d__Bacteria;p__Proteobacteria;c__Alphaproteobacteria;o__Rhizobiales;f__Xanthobacteraceae;g__Bradyrhizobium;s__	N/A	N/A
MAGs.BN11_3.Bin39	d__Bacteria;p__Firmicutes;c__Bacilli;o__Lactobacillales;f__Lactobacillaceae;g__Limosilactobacillus;s__Limosilactobacillus panis	GCF_001435935.1	99.1
MAGs.BN11_3.Bin40	d__Bacteria;p__Firmicutes;c__Bacilli;o__Lactobacillales;f__Lactobacillaceae;g__Limosilactobacillus;s__Limosilactobacillus mucosae	GCF_001436025.1	97.25
MAGs.BN11_3.Bin42	d__Bacteria;p__Bacteroidota;c__Bacteroidia;o__Chitinophagales;f__Chitinophagaceae;g__Sediminibacterium;s__Sediminibacterium magnilacihabitans	GCA_002973575.1	98.13
MAGs.BN11_3.Bin43	d__Bacteria;p__Firmicutes;c__Bacilli;o__Lactobacillales;f__Lactobacillaceae;g__Lactobacillus;s__Lactobacillus amylovorus	GCF_002706375.1	98.88
MAGs.BN11_3.Bin45	d__Bacteria;p__Firmicutes;c__Bacilli;o__Lactobacillales;f__Lactobacillaceae;g__Limosilactobacillus;s__	N/A	N/A
MAGs.BN11_3.Bin47	d__Bacteria;p__Firmicutes;c__Bacilli;o__Lactobacillales;f__Lactobacillaceae;g__Limosilactobacillus;s__	N/A	N/A
MAGs.BN11_3.Bin6	d__Bacteria;p__Firmicutes;c__Bacilli;o__Bacillales_B;f__Caldibacillaceae;g__Bacillus_J;s__Bacillus_J thermoamylovorans	GCF_000751775.1	98.51
MAGs.BN11_3.Bin9	d__Bacteria;p__Firmicutes;c__Bacilli;o__Lactobacillales;f__Lactobacillaceae;g__Lactobacillus;s__	N/A	N/A
MAGs.BN13_1.Bin10	d__Bacteria;p__Firmicutes;c__Bacilli;o__Bacillales_B;f__Bacillaceae_C;g__Weizmannia;s__Weizmannia coagulans	GCF_000290615.1	98.49
MAGs.BN13_1.Bin11	d__Bacteria;p__Firmicutes;c__Bacilli;o__Lactobacillales;f__Lactobacillaceae;g__Limosilactobacillus;s__	N/A	N/A
MAGs.BN13_1.Bin12	d__Bacteria;p__Firmicutes;c__Bacilli;o__Lactobacillales;f__Lactobacillaceae;g__Limosilactobacillus;s__	N/A	N/A
MAGs.BN13_1.Bin15	d__Bacteria;p__Firmicutes;c__Bacilli;o__Lactobacillales;f__Lactobacillaceae;g__Liquorilact	GCF_001434225.1	98.58

	obacillus;s__Liquorilactobacillus nagelii		
MAGs.BN13_1.Bin16	d__Bacteria;p__Firmicutes;c__Bacilli;o__Lactobacillales;f__Lactobacillaceae;g__Lactobacillus;s__Lactobacillus acetotolerans	GCF_001436775.1	99.12
MAGs.BN13_1.Bin2	d__Bacteria;p__Proteobacteria;c__Alphaproteobacteria;o__Rhizobiales;f__Xanthobacteraceae;g__Bradyrhizobium;s__	N/A	N/A
MAGs.BN13_1.Bin20	d__Bacteria;p__Firmicutes;c__Bacilli;o__Lactobacillales;f__Lactobacillaceae;g__Lactobacillus;s__Lactobacillus amylovorus	GCF_002706375.1	99.09
MAGs.BN13_1.Bin21	d__Bacteria;p__Firmicutes;c__Bacilli;o__Bacillales_D;f__Amphibacillaceae;g__;s__	N/A	N/A
MAGs.BN13_1.Bin23	d__Bacteria;p__Firmicutes;c__Bacilli;o__Lactobacillales;f__Lactobacillaceae;g__Liquorilactobacillus;s__Liquorilactobacillus vini	GCF_000255495.1	98.87
MAGs.BN13_1.Bin25	d__Bacteria;p__Bacteroidota;c__Bacteroidia;o__Chitinophagales;f__Chitinophagaceae;g__Sediminibacterium;s__Sediminibacterium magnilacihabitans	GCA_002973575.1	98.22
MAGs.BN13_1.Bin28	d__Bacteria;p__Proteobacteria;c__Gammaproteobacteria;o__Xanthomonadales;f__Xanthomonadaceae;g__Xanthomonas_B;s__Xanthomonas_B massiliensis	GCF_900018785.1	98.79
MAGs.BN13_1.Bin29	d__Bacteria;p__Firmicutes;c__Bacilli;o__Lactobacillales;f__Lactobacillaceae;g__Limosilactobacillus;s__Limosilactobacillus fermentum	GCF_000159215.1	98.09
MAGs.BN13_1.Bin30	d__Bacteria;p__Proteobacteria;c__Alphaproteobacteria;o__Acetobacterales;f__Acetobacteraceae;g__Acetobacter;s__Acetobacter pomorum	GCF_002738225.1	95.75
MAGs.BN13_1.Bin7	d__Bacteria;p__Firmicutes;c__Bacilli;o__Lactobacillales;f__Lactobacillaceae;g__Acetilactobacillus;s__Acetilactobacillus jinshanensis	GCF_004359375.1	99.98
MAGs.BN13_1.Bin9	d__Bacteria;p__Firmicutes;c__Bacilli;o__Bacillales_B;f__Caldibacillaceae;g__Bacillus_J;s__Bacillus_J thermoamylovorans	GCF_000751775.1	98.55
MAGs.BN13_2.Bin10	d__Bacteria;p__Firmicutes;c__Bacilli;o__Lactobacillales;f__Lactobacillaceae;g__Lactobacillus;s__	N/A	N/A
MAGs.BN13_2.Bin13	d__Bacteria;p__Firmicutes;c__Bacilli;o__Bacillales_B;f__Caldibacillaceae;g__Bacillus_J;s__Bacillus_J thermoamylovorans	GCF_000751775.1	98.43

MAGs.BN13_2.Bin14	d__Bacteria;p__Proteobacteria;c__Alphaproteobacteria;o__Rhizobiales;f__Xanthobacteraceae;g__Bradyrhizobium;s__	N/A	N/A
MAGs.BN13_2.Bin15	d__Bacteria;p__Proteobacteria;c__Gammaproteobacteria;o__Xanthomonadales;f__Xanthomonadaceae;g__Xanthomonas_B;s__Xanthomonas_B massiliensis	GCF_900018785.1	98.74
MAGs.BN13_2.Bin16	d__Bacteria;p__Firmicutes;c__Bacilli;o__Lactobacillales;f__Lactobacillaceae;g__Lactobacillus;s__Lactobacillus amylovorus	GCF_002706375.1	98.79
MAGs.BN13_2.Bin17	d__Bacteria;p__Firmicutes;c__Bacilli;o__Lactobacillales;f__Lactobacillaceae;g__Pediococcus;s__Pediococcus acidilactici	GCF_000146325.1	99.11
MAGs.BN13_2.Bin19	d__Bacteria;p__Firmicutes;c__Bacilli;o__Lactobacillales;f__Lactobacillaceae;g__Limosilactobacillus;s__Limosilactobacillus panis	GCF_001435935.1	99.01
MAGs.BN13_2.Bin21	d__Bacteria;p__Firmicutes;c__Bacilli;o__Lactobacillales;f__Lactobacillaceae;g__Limosilactobacillus;s__	N/A	N/A
MAGs.BN13_2.Bin22	d__Bacteria;p__Firmicutes;c__Bacilli;o__Lactobacillales;f__Lactobacillaceae;g__Limosilactobacillus;s__	N/A	N/A
MAGs.BN13_2.Bin23	d__Bacteria;p__Firmicutes;c__Bacilli;o__Lactobacillales;f__Lactobacillaceae;g__Limosilactobacillus;s__	N/A	N/A
MAGs.BN13_2.Bin26	d__Bacteria;p__Proteobacteria;c__Alphaproteobacteria;o__Acetobacterales;f__Acetobacteraceae;g__Acetobacter;s__Acetobacter pomorum	GCF_002738225.1	95.56
MAGs.BN13_2.Bin31	d__Bacteria;p__Firmicutes;c__Bacilli;o__Bacillales_B;f__Bacillaceae_C;g__Weizmannia;s__Weizmannia coagulans	GCF_000290615.1	98.58
MAGs.BN13_2.Bin35	d__Bacteria;p__Firmicutes;c__Bacilli;o__Lactobacillales;f__Lactobacillaceae;g__Limosilactobacillus;s__Limosilactobacillus pontis_A	GCF_002940945.1	98.35
MAGs.BN13_2.Bin38	d__Bacteria;p__Firmicutes;c__Bacilli;o__Lactobacillales;f__Lactobacillaceae;g__Lactobacillus;s__Lactobacillus acetotolerans	GCF_001436775.1	99.22
MAGs.BN13_2.Bin39	d__Bacteria;p__Firmicutes;c__Bacilli;o__Lactobacillales;f__Lactobacillaceae;g__Limosilactobacillus;s__	N/A	N/A

MAGs.BN13_2.Bin44	d__Bacteria;p__Bacteroidota;c__Bacteroidia;o__Chitinophagales;f__Chitinophagaceae;g__Sediminibacterium;s__Sediminibacterium magnilacihabitans	GCA_002973575.1	98.45
MAGs.BN13_2.Bin6	d__Bacteria;p__Firmicutes;c__Bacilli;o__Lactobacillales;f__Lactobacillaceae;g__Acetilactobacillus;s__Acetilactobacillus jinshanensis	GCF_004359375.1	99.96
MAGs.BN13_2.Bin7	d__Bacteria;p__Firmicutes;c__Bacilli;o__Bacillales_D;f__Amphibacillaceae;g__;s__	N/A	N/A
MAGs.BN13_2.Bin8	d__Bacteria;p__Firmicutes;c__Bacilli;o__Lactobacillales;f__Lactobacillaceae;g__Limosilactobacillus;s__Limosilactobacillus fermentum	GCF_000159215.1	98.15
MAGs.BN13_3.Bin12	d__Bacteria;p__Firmicutes;c__Bacilli;o__Lactobacillales;f__Lactobacillaceae;g__Liquorilactobacillus;s__Liquorilactobacillus nagelii	GCF_001434225.1	98.68
MAGs.BN13_3.Bin13	d__Bacteria;p__Firmicutes;c__Bacilli;o__Lactobacillales;f__Lactobacillaceae;g__Limosilactobacillus;s__Limosilactobacillus pontis_A	GCF_002940945.1	98.09
MAGs.BN13_3.Bin14	d__Bacteria;p__Firmicutes;c__Bacilli;o__Lactobacillales;f__Lactobacillaceae;g__Limosilactobacillus;s__	N/A	N/A
MAGs.BN13_3.Bin16	d__Bacteria;p__Firmicutes;c__Bacilli;o__Lactobacillales;f__Lactobacillaceae;g__Limosilactobacillus;s__	N/A	N/A
MAGs.BN13_3.Bin17	d__Bacteria;p__Firmicutes;c__Bacilli;o__Bacillales_B;f__Caldibacillaceae;g__Bacillus_J;s__Bacillus_J thermoamylovorans	GCF_000751775.1	98.53
MAGs.BN13_3.Bin18	d__Bacteria;p__Proteobacteria;c__Alphaproteobacteria;o__Rhizobiales;f__Xanthobacteraceae;g__Bradyrhizobium;s__	N/A	N/A
MAGs.BN13_3.Bin19	d__Bacteria;p__Firmicutes;c__Bacilli;o__Lactobacillales;f__Lactobacillaceae;g__Pediococcus;s__Pediococcus acidilactici	GCF_000146325.1	98.76
MAGs.BN13_3.Bin20	d__Bacteria;p__Proteobacteria;c__Alphaproteobacteria;o__Acetobacterales;f__Acetobacteraceae;g__Acetobacter;s__Acetobacter pomorum	GCF_002738225.1	95.56
MAGs.BN13_3.Bin21	d__Bacteria;p__Proteobacteria;c__Alphaproteobacteria;o__Acetobacterales;f__Acetobacteraceae;g__Acetobacter;s__Acetobacter peroxydans	GCF_006539345.1	97.55
MAGs.BN13_3.Bin23	d__Bacteria;p__Firmicutes;c__Bacilli;o__Lactobacillales;f__Lactobacillaceae;g__Lactobacill	GCF_002706375.1	98.78

	us;s__Lactobacillus amylovorus		
MAGs.BN13_3.Bin26	d__Bacteria;p__Firmicutes;c__Bacilli;o__Lactobacillales;f__Lactobacillaceae;g__Lactobacillus;s__	N/A	N/A
MAGs.BN13_3.Bin27	d__Bacteria;p__Firmicutes;c__Bacilli;o__Lactobacillales;f__Lactobacillaceae;g__Limosilactobacillus;s__Limosilactobacillus fermentum	GCF_000159215.1	98.02
MAGs.BN13_3.Bin30	d__Bacteria;p__Firmicutes;c__Bacilli;o__Lactobacillales;f__Lactobacillaceae;g__Acetilactobacillus;s__Acetilactobacillus jinshanensis	GCF_004359375.1	99.97
MAGs.BN13_3.Bin31	d__Bacteria;p__Bacteroidota;c__Bacteroidia;o__Chitinophagales;f__Chitinophagaceae;g__Sediminibacterium;s__Sediminibacterium magnilacihabitans	GCA_002973575.1	98.11
MAGs.BN13_3.Bin36	d__Bacteria;p__Firmicutes;c__Bacilli;o__Lactobacillales;f__Lactobacillaceae;g__Lactobacillus;s__	N/A	N/A
MAGs.BN13_3.Bin37	d__Bacteria;p__Firmicutes;c__Bacilli;o__Lactobacillales;f__Lactobacillaceae;g__Limosilactobacillus;s__	N/A	N/A
MAGs.BN13_3.Bin38	d__Bacteria;p__Firmicutes;c__Bacilli;o__Lactobacillales;f__Lactobacillaceae;g__Limosilactobacillus;s__	N/A	N/A
MAGs.BN13_3.Bin39	d__Bacteria;p__Firmicutes;c__Bacilli;o__Bacillales_D;f__Amphibacillaceae;g__;s__	N/A	N/A
MAGs.BN13_3.Bin4	d__Bacteria;p__Firmicutes;c__Bacilli;o__Lactobacillales;f__Lactobacillaceae;g__Limosilactobacillus;s__	N/A	N/A
MAGs.BN13_3.Bin42	d__Bacteria;p__Firmicutes;c__Bacilli;o__Bacillales_B;f__Bacillaceae_C;g__Weizmannia;s__Weizmannia coagulans	GCF_000290615.1	98.65
MAGs.BN13_3.Bin43	d__Bacteria;p__Firmicutes;c__Bacilli;o__Lactobacillales;f__Lactobacillaceae;g__Limosilactobacillus;s__Limosilactobacillus panis	GCF_001435935.1	99.34
MAGs.BN13_3.Bin44	d__Bacteria;p__Firmicutes;c__Bacilli;o__Lactobacillales;f__Lactobacillaceae;g__Liquorilactobacillus;s__Liquorilactobacillus vini	GCF_000255495.1	98.85
MAGs.BN13_3.Bin7	d__Bacteria;p__Firmicutes;c__Bacilli;o__Lactobacillales;f__Lactobacillaceae;g__Limosilactobacillus;s__Limosilactobacillus secaliphilus	GCF_001437055.1	99.58

MAGs.BN13_3.Bin9	d__Bacteria;p__Firmicutes;c__Bacilli;o__Lactobacillales;f__Lactobacillaceae;g__Lactobacillus;s__Lactobacillus acetotolerans	GCF_001436775.1	98.99
MAGs.BN15_1.Bin10	d__Bacteria;p__Firmicutes;c__Bacilli;o__Lactobacillales;f__Lactobacillaceae;g__Limosilactobacillus;s__Limosilactobacillus panis	GCF_001435935.1	99.24
MAGs.BN15_1.Bin11	d__Bacteria;p__Firmicutes;c__Bacilli;o__Lactobacillales;f__Lactobacillaceae;g__Lactobacillus;s__Lactobacillus amylovorus	GCF_002706375.1	99.21
MAGs.BN15_1.Bin13	d__Bacteria;p__Firmicutes;c__Bacilli;o__Lactobacillales;f__Lactobacillaceae;g__Liquorilactobacillus;s__Liquorilactobacillus vini	GCF_000255495.1	98.8
MAGs.BN15_1.Bin15	d__Bacteria;p__Proteobacteria;c__Alphaproteobacteria;o__Acetobacterales;f__Acetobacteraceae;g__Acetobacter;s__Acetobacter pomorum	GCF_002738225.1	95.7
MAGs.BN15_1.Bin16	d__Bacteria;p__Firmicutes;c__Bacilli;o__Lactobacillales;f__Lactobacillaceae;g__Lactobacillus;s__Lactobacillus acetotolerans	GCF_001436775.1	99.2
MAGs.BN15_1.Bin18	d__Bacteria;p__Firmicutes;c__Bacilli;o__Bacillales_D;f__Amphibacillaceae;g__;s__	N/A	N/A
MAGs.BN15_1.Bin2	d__Bacteria;p__Firmicutes;c__Bacilli;o__Lactobacillales;f__Lactobacillaceae;g__Acetilactobacillus;s__Acetilactobacillus jinshanensis	GCF_004359375.1	99.96
MAGs.BN15_1.Bin22	d__Bacteria;p__Firmicutes;c__Bacilli;o__Bacillales_B;f__Bacillaceae_C;g__Weizmannia;s__Weizmannia coagulans	GCF_000290615.1	98.49
MAGs.BN15_1.Bin25	d__Bacteria;p__Bacteroidota;c__Bacteroidia;o__Chitinophagales;f__Chitinophagaceae;g__Sediminibacterium;s__Sediminibacterium magnilacihabitans	GCA_002973575.1	98.5
MAGs.BN15_1.Bin26	d__Bacteria;p__Firmicutes;c__Bacilli;o__Lactobacillales;f__Lactobacillaceae;g__Limosilactobacillus;s__	N/A	N/A
MAGs.BN15_1.Bin27	d__Bacteria;p__Firmicutes;c__Bacilli;o__Lactobacillales;f__Lactobacillaceae;g__Limosilactobacillus;s__Limosilactobacillus fermentum	GCF_000159215.1	98.07
MAGs.BN15_1.Bin4	d__Bacteria;p__Firmicutes;c__Bacilli;o__Lactobacillales;f__Lactobacillaceae;g__Liquorilactobacillus;s__Liquorilactobacillus nagelii	GCF_001434225.1	98.69
MAGs.BN15_1.Bin5	d__Bacteria;p__Proteobacteria;c__Alphaproteobacteria;o__Rhizobiales;f__Xanthobacteracea	N/A	N/A

	e;g__Bradyrhizobium;s__		
MAGs.BN15_1.Bin8	d__Bacteria;p__Firmicutes;c__Bacilli;o__Lactobacillales;f__Lactobacillaceae;g__Limosilactobacillus;s__	N/A	N/A
MAGs.BN15_1.Bin9	d__Bacteria;p__Firmicutes;c__Bacilli;o__Bacillales_B;f__Caldibacillaceae;g__Bacillus_J;s__Bacillus_J thermoamylovorans	GCF_000751775.1	98.59
MAGs.BN15_2.Bin1	d__Bacteria;p__Firmicutes;c__Bacilli;o__Lactobacillales;f__Lactobacillaceae;g__Limosilactobacillus;s__Limosilactobacillus fermentum	GCF_000159215.1	98.2
MAGs.BN15_2.Bin13	d__Bacteria;p__Firmicutes;c__Bacilli;o__Bacillales_B;f__Bacillaceae_C;g__Weizmannia;s__Weizmannia coagulans	GCF_000290615.1	98.68
MAGs.BN15_2.Bin14	d__Bacteria;p__Firmicutes;c__Bacilli;o__Lactobacillales;f__Lactobacillaceae;g__Lactobacillus;s__Lactobacillus acetotolerans	GCF_001436775.1	99.23
MAGs.BN15_2.Bin17	d__Bacteria;p__Bacteroidota;c__Bacteroidia;o__Chitinophagales;f__Chitinophagaceae;g__Sediminibacterium;s__Sediminibacterium magnilacihabitans	GCA_002973575.1	98.11
MAGs.BN15_2.Bin19	d__Bacteria;p__Actinobacteriota;c__Actinomycetia;o__Mycobacteriales;f__Mycobacteriaceae;g__Mycobacterium;s__Mycobacterium aubagnense	GCF_010730955.1	95.33
MAGs.BN15_2.Bin20	d__Bacteria;p__Firmicutes;c__Bacilli;o__Lactobacillales;f__Lactobacillaceae;g__Limosilactobacillus;s__	N/A	N/A
MAGs.BN15_2.Bin22	d__Bacteria;p__Proteobacteria;c__Alphaproteobacteria;o__Rhizobiales;f__Xanthobacteraceae;g__Afipia;s__	N/A	N/A
MAGs.BN15_2.Bin25	d__Bacteria;p__Firmicutes;c__Bacilli;o__Lactobacillales;f__Lactobacillaceae;g__Acetilactobacillus;s__Acetilactobacillus jinshanensis	GCF_004359375.1	99.97
MAGs.BN15_2.Bin3	d__Bacteria;p__Firmicutes;c__Bacilli;o__Bacillales_B;f__Caldibacillaceae;g__Bacillus_J;s__Bacillus_J thermoamylovorans	GCF_000751775.1	98.34
MAGs.BN15_2.Bin30	d__Bacteria;p__Firmicutes;c__Bacilli;o__Lactobacillales;f__Lactobacillaceae;g__Limosilactobacillus;s__Limosilactobacillus panis	GCF_001435935.1	99.25
MAGs.BN15_2.Bin32	d__Bacteria;p__Firmicutes;c__Bacilli;o__Lactobacillales;f__Lactobacillaceae;g__Lactobacill	N/A	N/A

	us;s__		
MAGs.BN15_2.Bin33	d__Bacteria;p__Proteobacteria;c__Alphaproteobacteria;o__Rhizobiales;f__Xanthobacteraceae;g__Bradyrhizobium;s__	N/A	N/A
MAGs.BN15_2.Bin36	d__Bacteria;p__Firmicutes;c__Bacilli;o__Lactobacillales;f__Lactobacillaceae;g__Pediococcus;s__Pediococcus acidilactici	GCF_000146325.1	99.11
MAGs.BN15_2.Bin37	d__Bacteria;p__Firmicutes;c__Bacilli;o__Lactobacillales;f__Lactobacillaceae;g__Lactobacillus;s__Lactobacillus amylovorus	GCF_002706375.1	98.89
MAGs.BN15_2.Bin38	d__Bacteria;p__Proteobacteria;c__Alphaproteobacteria;o__Rhizobiales;f__Beijerinckiaceae;g__Methylobacterium;s__	N/A	N/A
MAGs.BN15_2.Bin40	d__Bacteria;p__Firmicutes;c__Bacilli;o__Bacillales_D;f__Amphibacillaceae;g__;s__	N/A	N/A
MAGs.BN15_2.Bin5	d__Bacteria;p__Firmicutes;c__Bacilli;o__Lactobacillales;f__Lactobacillaceae;g__Limosilactobacillus;s__	N/A	N/A
MAGs.BN15_2.Bin9	d__Bacteria;p__Proteobacteria;c__Gammaproteobacteria;o__Xanthomonadales;f__Xanthomonadaceae;g__Xanthomonas_B;s__Xanthomonas_B massiliensis	GCF_900018785.1	98.8
MAGs.BN15_3.Bin1	d__Bacteria;p__Firmicutes;c__Bacilli;o__Lactobacillales;f__Lactobacillaceae;g__Limosilactobacillus;s__	N/A	N/A
MAGs.BN15_3.Bin12	d__Bacteria;p__Firmicutes;c__Bacilli;o__Lactobacillales;f__Lactobacillaceae;g__Liquorilactobacillus;s__Liquorilactobacillus vini	GCF_000255495.1	98.97
MAGs.BN15_3.Bin13	d__Bacteria;p__Firmicutes;c__Bacilli;o__Bacillales_D;f__Amphibacillaceae;g__;s__	N/A	N/A
MAGs.BN15_3.Bin14	d__Bacteria;p__Firmicutes;c__Bacilli;o__Lactobacillales;f__Lactobacillaceae;g__Lactobacillus;s__Lactobacillus acetotolerans	GCF_001436775.1	99.28
MAGs.BN15_3.Bin15	d__Bacteria;p__Firmicutes;c__Bacilli;o__Lactobacillales;f__Lactobacillaceae;g__Limosilactobacillus;s__Limosilactobacillus fermentum	GCF_000159215.1	98.24
MAGs.BN15_3.Bin20	d__Bacteria;p__Actinobacteriota;c__Actinomycetia;o__Mycobacteriales;f__Mycobacteriaceae;g__Mycobacterium;s__Mycobacterium aubagnense	GCF_010730955.1	95.61
MAGs.BN15_3.Bin22	d__Bacteria;p__Firmicutes;c__Bacilli;o__Lactobacillales;f__Lactobacillaceae;g__Limosilact	N/A	N/A

	obacillus;s__		
MAGs.BN15_3.Bin23	d__Bacteria;p__Firmicutes;c__Bacilli;o__Lactobacillales;f__Lactobacillaceae;g__Liquorilactobacillus;s__Liquorilactobacillus nagelii	GCF_001434225.1	98.56
MAGs.BN15_3.Bin26	d__Bacteria;p__Firmicutes;c__Bacilli;o__Lactobacillales;f__Lactobacillaceae;g__Lactobacillus;s__Lactobacillus amylovorus	GCF_002706375.1	99.07
MAGs.BN15_3.Bin27	d__Bacteria;p__Firmicutes;c__Bacilli;o__Lactobacillales;f__Lactobacillaceae;g__Limosilactobacillus;s__	N/A	N/A
MAGs.BN15_3.Bin29	d__Bacteria;p__Proteobacteria;c__Alphaproteobacteria;o__Acetobacterales;f__Acetobacteraceae;g__Acetobacter;s__Acetobacter pomorum	GCF_002738225.1	95.74
MAGs.BN15_3.Bin30	d__Bacteria;p__Firmicutes;c__Bacilli;o__Lactobacillales;f__Lactobacillaceae;g__Lactobacillus;s__	N/A	N/A
MAGs.BN15_3.Bin31	d__Bacteria;p__Firmicutes;c__Bacilli;o__Lactobacillales;f__Lactobacillaceae;g__Pediococcus;s__Pediococcus acidilactici	GCF_000146325.1	98.95
MAGs.BN15_3.Bin32	d__Bacteria;p__Firmicutes;c__Bacilli;o__Lactobacillales;f__Lactobacillaceae;g__Acetilactobacillus;s__Acetilactobacillus jinshanensis	GCF_004359375.1	99.97
MAGs.BN15_3.Bin37	d__Bacteria;p__Firmicutes;c__Bacilli;o__Bacillales_B;f__Bacillaceae_C;g__Weizmannia;s__Weizmannia coagulans	GCF_000290615.1	98.59
MAGs.BN15_3.Bin4	d__Bacteria;p__Proteobacteria;c__Alphaproteobacteria;o__Rhizobiales;f__Xanthobacteraceae;g__Bradyrhizobium;s__	N/A	N/A
MAGs.BN15_3.Bin5	d__Bacteria;p__Firmicutes;c__Bacilli;o__Bacillales_B;f__Bacillaceae_C;g__Heyndrickxia;s__Heyndrickxia sporothermodurans	GCF_003055085.1	98.09
MAGs.BN15_3.Bin6	d__Bacteria;p__Firmicutes;c__Bacilli;o__Lactobacillales;f__Lactobacillaceae;g__Limosilactobacillus;s__	N/A	N/A
MAGs.BN15_3.Bin7	d__Bacteria;p__Bacteroidota;c__Bacteroidia;o__Chitinophagales;f__Chitinophagaceae;g__Sediminibacterium;s__Sediminibacterium magnilacihabitans	GCA_002973575.1	98.12
MAGs.BN15_3.Bin8	d__Bacteria;p__Firmicutes;c__Bacilli;o__Lactobacillales;f__Lactobacillaceae;g__Lactobacill	N/A	N/A

	us;s__		
MAGs.BN15_3.Bin9	d__Bacteria;p__Firmicutes;c__Bacilli;o__Bacillales_B;f__Caldibacillaceae;g__Bacillus_J;s__Bacillus_J thermoamylovorans	GCF_000751775.1	98.55
MAGs.BN17_1.Bin1	d__Bacteria;p__Firmicutes;c__Bacilli;o__Lactobacillales;f__Lactobacillaceae;g__Lactobacillus;s__Lactobacillus amylovorus	GCF_002706375.1	98.37
MAGs.BN17_1.Bin12	d__Bacteria;p__Firmicutes;c__Bacilli;o__Lactobacillales;f__Lactobacillaceae;g__Liquorilactobacillus;s__Liquorilactobacillus vini	GCF_000255495.1	98.9
MAGs.BN17_1.Bin15	d__Bacteria;p__Firmicutes;c__Bacilli;o__Bacillales_D;f__Amphibacillaceae;g__;s__	N/A	N/A
MAGs.BN17_1.Bin17	d__Bacteria;p__Firmicutes;c__Bacilli;o__Bacillales_B;f__Bacillaceae_C;g__Weizmannia;s__Weizmannia coagulans	GCF_000290615.1	98.63
MAGs.BN17_1.Bin18	d__Bacteria;p__Firmicutes;c__Bacilli;o__Lactobacillales;f__Lactobacillaceae;g__Liquorilactobacillus;s__Liquorilactobacillus nagelii	GCF_001434225.1	98.71
MAGs.BN17_1.Bin2	d__Bacteria;p__Firmicutes;c__Bacilli;o__Bacillales_B;f__Caldibacillaceae;g__Bacillus_J;s__Bacillus_J thermoamylovorans	GCF_000751775.1	98.29
MAGs.BN17_1.Bin20	d__Bacteria;p__Firmicutes;c__Bacilli;o__Lactobacillales;f__Lactobacillaceae;g__Limosilactobacillus;s__	N/A	N/A
MAGs.BN17_1.Bin21	d__Bacteria;p__Firmicutes;c__Bacilli;o__Lactobacillales;f__Lactobacillaceae;g__Limosilactobacillus;s__Limosilactobacillus fermentum	GCF_000159215.1	98.15
MAGs.BN17_1.Bin22	d__Bacteria;p__Firmicutes;c__Bacilli;o__Paenibacillales;f__Paenibacillaceae;g__Paenibacillus;s__Paenibacillus sp000787385	GCF_000787385.1	98.91
MAGs.BN17_1.Bin23	d__Bacteria;p__Bacteroidota;c__Bacteroidia;o__Chitinophagales;f__Chitinophagaceae;g__Sediminibacterium;s__Sediminibacterium magnilacihabitans	GCA_002973575.1	98.1
MAGs.BN17_1.Bin24	d__Bacteria;p__Proteobacteria;c__Alphaproteobacteria;o__Rhizobiales;f__Xanthobacteraceae;g__Bradyrhizobium;s__	N/A	N/A
MAGs.BN17_1.Bin25	d__Bacteria;p__Firmicutes;c__Bacilli;o__Lactobacillales;f__Lactobacillaceae;g__Limosilactobacillus;s__Limosilactobacillus panis	GCF_001435935.1	99.2

MAGs.BN17_1.Bin3	d__Bacteria;p__Firmicutes;c__Bacilli;o__Lactobacillales;f__Lactobacillaceae;g__Acetilactobacillus;s__Acetilactobacillus jinshanensis	GCF_004359375.1	99.97
MAGs.BN17_1.Bin5	d__Bacteria;p__Firmicutes;c__Bacilli;o__Bacillales_A;f__Planococcaceae;g__Rummeliibacillus;s__Rummeliibacillus sp003576525	GCF_003576525.1	99.08
MAGs.BN17_1.Bin6	d__Bacteria;p__Firmicutes;c__Bacilli;o__Lactobacillales;f__Lactobacillaceae;g__Lactobacillus;s__Lactobacillus acetotolerans	GCF_001436775.1	99.16
MAGs.BN17_1.Bin8	d__Bacteria;p__Proteobacteria;c__Gammaproteobacteria;o__Xanthomonadales;f__Xanthomonadaceae;g__Xanthomonas_B;s__Xanthomonas_B massiliensis	GCF_900018785.1	98.73
MAGs.BN17_2.Bin1	d__Bacteria;p__Firmicutes;c__Bacilli;o__Lactobacillales;f__Lactobacillaceae;g__Lactobacillus;s__	N/A	N/A
MAGs.BN17_2.Bin10	d__Bacteria;p__Proteobacteria;c__Alphaproteobacteria;o__Rhizobiales;f__Xanthobacteraceae;g__Afipia;s__	N/A	N/A
MAGs.BN17_2.Bin11	d__Bacteria;p__Firmicutes;c__Bacilli;o__Bacillales_B;f__Caldibacillaceae;g__Bacillus_J;s__Bacillus_J thermoamylovorans	GCF_000751775.1	98.58
MAGs.BN17_2.Bin13	d__Bacteria;p__Proteobacteria;c__Alphaproteobacteria;o__Rhizobiales;f__Rhizobiaceae;g__Mesorhizobium;s__Mesorhizobium terrae	GCF_008727715.1	99.36
MAGs.BN17_2.Bin14	d__Bacteria;p__Firmicutes;c__Bacilli;o__Bacillales_D;f__Amphibacillaceae;g__;s__	N/A	N/A
MAGs.BN17_2.Bin15	d__Bacteria;p__Firmicutes;c__Bacilli;o__Lactobacillales;f__Lactobacillaceae;g__Acetilactobacillus;s__Acetilactobacillus jinshanensis	GCF_004359375.1	99.96
MAGs.BN17_2.Bin16	d__Bacteria;p__Firmicutes;c__Bacilli;o__Lactobacillales;f__Lactobacillaceae;g__Lactobacillus;s__Lactobacillus amylovorus	GCF_002706375.1	98.81
MAGs.BN17_2.Bin2	d__Bacteria;p__Firmicutes;c__Bacilli;o__Lactobacillales;f__Lactobacillaceae;g__Pediococcus;s__Pediococcus acidilactici	GCF_000146325.1	99.07
MAGs.BN17_2.Bin20	d__Bacteria;p__Firmicutes;c__Bacilli;o__Lactobacillales;f__Lactobacillaceae;g__Lactobacillus;s__Lactobacillus acetotolerans	GCF_001436775.1	99.18
MAGs.BN17_2.Bin21	d__Bacteria;p__Firmicutes;c__Bacilli;o__Paenibacillales;f__Paenibacillaceae;g__Paenibacill	GCF_000787385.1	98.84

	us;s__Paenibacillus sp000787385		
MAGs.BN17_2.Bin22	d__Bacteria;p__Firmicutes;c__Bacilli;o__Lactobacillales;f__Lactobacillaceae;g__Limosilactobacillus;s__	N/A	N/A
MAGs.BN17_2.Bin23	d__Bacteria;p__Firmicutes;c__Bacilli;o__Lactobacillales;f__Lactobacillaceae;g__Limosilactobacillus;s__	N/A	N/A
MAGs.BN17_2.Bin25	d__Bacteria;p__Firmicutes;c__Bacilli;o__Bacillales_B;f__Bacillaceae_C;g__Weizmannia;s__Weizmannia coagulans	GCF_000290615.1	98.72
MAGs.BN17_2.Bin26	d__Bacteria;p__Proteobacteria;c__Alphaproteobacteria;o__Rhizobiales;f__Beijerinckiaceae;g__Methylovirgula;s__	N/A	N/A
MAGs.BN17_2.Bin29	d__Bacteria;p__Firmicutes;c__Bacilli;o__Lactobacillales;f__Lactobacillaceae;g__Limosilactobacillus;s__	N/A	N/A
MAGs.BN17_2.Bin34	d__Bacteria;p__Firmicutes;c__Bacilli;o__Bacillales_B;f__Bacillaceae_C;g__Heyndrickxia;s__Heyndrickxia sporothermodurans	GCF_003055085.1	98.14
MAGs.BN17_2.Bin36	d__Bacteria;p__Bacteroidota;c__Bacteroidia;o__Chitinophagales;f__Chitinophagaceae;g__Sediminibacterium;s__Sediminibacterium magnilacihabitans	GCA_002973575.1	98.13
MAGs.BN17_2.Bin37	d__Bacteria;p__Firmicutes;c__Bacilli;o__Lactobacillales;f__Lactobacillaceae;g__Liquorilactobacillus;s__Liquorilactobacillus vini	GCF_000255495.1	98.88
MAGs.BN17_2.Bin39	d__Bacteria;p__Firmicutes;c__Bacilli;o__Lactobacillales;f__Lactobacillaceae;g__Limosilactobacillus;s__Limosilactobacillus panis	GCF_001435935.1	98.96
MAGs.BN17_2.Bin4	d__Bacteria;p__Proteobacteria;c__Alphaproteobacteria;o__Rhizobiales;f__Xanthobacteraceae;g__Bradyrhizobium;s__	N/A	N/A
MAGs.BN17_2.Bin40	d__Bacteria;p__Actinobacteriota;c__Actinomycetia;o__Mycobacteriales;f__Mycobacteriaceae;g__Mycobacterium;s__Mycobacterium aubagnense	GCF_010730955.1	95.41
MAGs.BN17_2.Bin6	d__Bacteria;p__Firmicutes;c__Bacilli;o__Lactobacillales;f__Lactobacillaceae;g__Liquorilactobacillus;s__Liquorilactobacillus nagelii	GCF_001434225.1	98.75
MAGs.BN17_2.Bin9	d__Bacteria;p__Firmicutes;c__Bacilli;o__Lactobacillales;f__Lactobacillaceae;g__Limosilact	GCF_000159215.1	98.18

	obacillus;s__Limosilactobacillus fermentum		
MAGs.BN17_3.Bin10	d__Bacteria;p__Firmicutes;c__Bacilli;o__Bacillales_B;f__Caldibacillaceae;g__Bacillus_J;s__Bacillus_J thermoamylovorans	GCF_000751775.1	98.67
MAGs.BN17_3.Bin11	d__Bacteria;p__Proteobacteria;c__Alphaproteobacteria;o__Rhizobiales;f__Beijerinckiaceae;g__Methylovirgula;s__	N/A	N/A
MAGs.BN17_3.Bin12	d__Bacteria;p__Firmicutes;c__Bacilli;o__Lactobacillales;f__Lactobacillaceae;g__Lactobacillus;s__Lactobacillus acetotolerans	GCF_001436775.1	99.13
MAGs.BN17_3.Bin14	d__Bacteria;p__Firmicutes;c__Bacilli;o__Lactobacillales;f__Lactobacillaceae;g__Lactobacillus;s__	N/A	N/A
MAGs.BN17_3.Bin15	d__Bacteria;p__Firmicutes;c__Bacilli;o__Lactobacillales;f__Lactobacillaceae;g__Limosilactobacillus;s__	N/A	N/A
MAGs.BN17_3.Bin17	d__Bacteria;p__Firmicutes;c__Bacilli;o__Lactobacillales;f__Lactobacillaceae;g__Liquorilactobacillus;s__Liquorilactobacillus vini	GCF_000255495.1	98.8
MAGs.BN17_3.Bin18	d__Bacteria;p__Firmicutes;c__Bacilli;o__Lactobacillales;f__Lactobacillaceae;g__Limosilactobacillus;s__Limosilactobacillus panis	GCF_001435935.1	99.09
MAGs.BN17_3.Bin2	d__Bacteria;p__Firmicutes;c__Bacilli;o__Lactobacillales;f__Lactobacillaceae;g__Pediococcus;s__Pediococcus acidilactici	GCF_000146325.1	99.11
MAGs.BN17_3.Bin21	d__Bacteria;p__Actinobacteriota;c__Actinomycetia;o__Mycobacteriales;f__Mycobacteriaceae;g__Mycobacterium;s__Mycobacterium aubagnense	GCF_010730955.1	95.48
MAGs.BN17_3.Bin22	d__Bacteria;p__Proteobacteria;c__Alphaproteobacteria;o__Rhizobiales;f__Xanthobacteraceae;g__Bradyrhizobium;s__	N/A	N/A
MAGs.BN17_3.Bin23	d__Bacteria;p__Firmicutes;c__Bacilli;o__Bacillales_D;f__Amphibacillaceae;g__Oceanobacillus;s__Oceanobacillus caeni	GCF_001298135.1	98.51
MAGs.BN17_3.Bin26	d__Bacteria;p__Proteobacteria;c__Alphaproteobacteria;o__Rhizobiales;f__Xanthobacteraceae;g__Afipia;s__	N/A	N/A
MAGs.BN17_3.Bin27	d__Bacteria;p__Firmicutes;c__Bacilli;o__Lactobacillales;f__Lactobacillaceae;g__Liquorilact	GCF_001434225.1	98.79

	obacillus;s__Liquorilactobacillus nagelii		
MAGs.BN17_3.Bin28	d__Bacteria;p__Firmicutes;c__Bacilli;o__Bacillales_D;f__Amphibacillaceae;g__;s__	N/A	N/A
MAGs.BN17_3.Bin3	d__Bacteria;p__Firmicutes;c__Bacilli;o__Lactobacillales;f__Lactobacillaceae;g__Limosilactobacillus;s__Limosilactobacillus fermentum	GCF_000159215.1	98.22
MAGs.BN17_3.Bin4	d__Bacteria;p__Firmicutes;c__Bacilli;o__Lactobacillales;f__Lactobacillaceae;g__Lactobacillus;s__Lactobacillus amylovorus	GCF_002706375.1	98.63
MAGs.BN17_3.Bin5	d__Bacteria;p__Bacteroidota;c__Bacteroidia;o__Chitinophagales;f__Chitinophagaceae;g__Sediminibacterium;s__Sediminibacterium magnilacihabitans	GCA_002973575.1	98.09
MAGs.BN17_3.Bin6	d__Bacteria;p__Firmicutes;c__Bacilli;o__Lactobacillales;f__Lactobacillaceae;g__Acetilactobacillus;s__Acetilactobacillus jinshanensis	GCF_004359375.1	99.96
MAGs.BN17_3.Bin7	d__Bacteria;p__Firmicutes;c__Bacilli;o__Bacillales_B;f__Bacillaceae_C;g__Weizmannia;s__Weizmannia coagulans	GCF_000290615.1	98.59
MAGs.BN19_1.Bin1	d__Bacteria;p__Firmicutes;c__Bacilli;o__Lactobacillales;f__Lactobacillaceae;g__Lactobacillus;s__	N/A	N/A
MAGs.BN19_1.Bin10	d__Bacteria;p__Firmicutes;c__Bacilli;o__Lactobacillales;f__Lactobacillaceae;g__Lactobacillus;s__	N/A	N/A
MAGs.BN19_1.Bin11	d__Bacteria;p__Firmicutes_C;c__Negativicutes;o__Veillonellales;f__Megasphaeraceae;g__Megasphaera;s__Megasphaera elsdenii	GCF_003010495.1	98.1
MAGs.BN19_1.Bin12	d__Bacteria;p__Firmicutes;c__Bacilli;o__Lactobacillales;f__Lactobacillaceae;g__Liquorilactobacillus;s__Liquorilactobacillus vini	GCF_000255495.1	98.86
MAGs.BN19_1.Bin13	d__Bacteria;p__Firmicutes;c__Bacilli;o__Lactobacillales;f__Lactobacillaceae;g__Lentilactobacillus;s__Lentilactobacillus raoultii	GCF_900411375.1	96.6
MAGs.BN19_1.Bin14	d__Bacteria;p__Firmicutes;c__Bacilli;o__Lactobacillales;f__Lactobacillaceae;g__Limosilactobacillus;s__	N/A	N/A
MAGs.BN19_1.Bin18	d__Bacteria;p__Firmicutes;c__Bacilli;o__Lactobacillales;f__Lactobacillaceae;g__Limosilactobacillus;s__	N/A	N/A

MAGs.BN19_1.Bin20	d__Bacteria;p__Firmicutes;c__Bacilli;o__Bacillales_D;f__Amphibacillaceae;g__;s__	N/A	N/A
MAGs.BN19_1.Bin21	d__Bacteria;p__Firmicutes;c__Bacilli;o__Lactobacillales;f__Lactobacillaceae;g__Lactobacillus;s__Lactobacillus acetotolerans	GCF_001436775.1	99.24
MAGs.BN19_1.Bin23	d__Bacteria;p__Firmicutes;c__Bacilli;o__Bacillales_A;f__Planococcaceae;g__Rummeliibacillus;s__Rummeliibacillus sp003576525	GCF_003576525.1	98.69
MAGs.BN19_1.Bin24	d__Bacteria;p__Proteobacteria;c__Alphaproteobacteria;o__Acetobacterales;f__Acetobacteraceae;g__Acetobacter;s__Acetobacter pomorum	GCF_002738225.1	95.88
MAGs.BN19_1.Bin25	d__Bacteria;p__Proteobacteria;c__Alphaproteobacteria;o__Rhizobiales;f__Beijerinckiaceae;g__Methylovirgula;s__	N/A	N/A
MAGs.BN19_1.Bin27	d__Bacteria;p__Firmicutes;c__Bacilli;o__Bacillales_B;f__Caldibacillaceae;g__Bacillus_J;s__Bacillus_J thermoamylovorans	GCF_000751775.1	98.36
MAGs.BN19_1.Bin28	d__Bacteria;p__Actinobacteriota;c__Actinomycetia;o__Actinomycetales;f__Bifidobacteriaceae;g__Bifidobacterium;s__Bifidobacterium thermophilum	GCF_000771265.1	95.43
MAGs.BN19_1.Bin29	d__Bacteria;p__Firmicutes;c__Bacilli;o__Lactobacillales;f__Lactobacillaceae;g__Limosilactobacillus;s__	N/A	N/A
MAGs.BN19_1.Bin30	d__Bacteria;p__Firmicutes;c__Bacilli;o__Paenibacillales;f__Paenibacillaceae;g__Paenibacillus;s__Paenibacillus sp000787385	GCF_000787385.1	98.85
MAGs.BN19_1.Bin31	d__Bacteria;p__Firmicutes;c__Bacilli;o__Bacillales_B;f__Bacillaceae_C;g__Weizmannia;s__Weizmannia coagulans	GCF_000290615.1	98.71
MAGs.BN19_1.Bin33	d__Bacteria;p__Proteobacteria;c__Gammaproteobacteria;o__Pseudomonadales;f__Moraxellaceae;g__Acinetobacter;s__Acinetobacter baumannii	GCF_009759685.1	98.16
MAGs.BN19_1.Bin36	d__Bacteria;p__Firmicutes;c__Bacilli;o__Lactobacillales;f__Lactobacillaceae;g__Acetilactobacillus;s__Acetilactobacillus jinshanensis	GCF_004359375.1	99.97
MAGs.BN19_1.Bin38	d__Bacteria;p__Proteobacteria;c__Alphaproteobacteria;o__Rhizobiales;f__Xanthobacteraceae;g__Bradyrhizobium;s__	N/A	N/A
MAGs.BN19_1.Bin41	d__Bacteria;p__Firmicutes;c__Bacilli;o__Lactobacillales;f__Lactobacillaceae;g__Limosilact	N/A	N/A

	obacillus;s__		
MAGs.BN19_1.Bin5	d__Bacteria;p__Firmicutes;c__Bacilli;o__Lactobacillales;f__Lactobacillaceae;g__Limosilactobacillus;s__	N/A	N/A
MAGs.BN19_1.Bin6	d__Bacteria;p__Bacteroidota;c__Bacteroidia;o__Chitinophagales;f__Chitinophagaceae;g__Sediminibacterium;s__Sediminibacterium magnilacihabitans	GCA_002973575.1	98.1
MAGs.BN19_1.Bin7	d__Bacteria;p__Firmicutes;c__Bacilli;o__Lactobacillales;f__Lactobacillaceae;g__Lactobacillus;s__Lactobacillus amylovorus	GCF_002706375.1	98.97
MAGs.BN19_2.Bin1	d__Bacteria;p__Firmicutes;c__Bacilli;o__Lactobacillales;f__Lactobacillaceae;g__Lactobacillus;s__Lactobacillus acetotolerans	GCF_001436775.1	99.1
MAGs.BN19_2.Bin10	d__Bacteria;p__Firmicutes;c__Bacilli;o__Lactobacillales;f__Lactobacillaceae;g__Acetilactobacillus;s__Acetilactobacillus jinshanensis	GCF_004359375.1	99.96
MAGs.BN19_2.Bin11	d__Bacteria;p__Bacteroidota;c__Bacteroidia;o__Chitinophagales;f__Chitinophagaceae;g__Sediminibacterium;s__Sediminibacterium magnilacihabitans	GCA_002973575.1	98.1
MAGs.BN19_2.Bin12	d__Bacteria;p__Proteobacteria;c__Alphaproteobacteria;o__Rhizobiales;f__Xanthobacteraceae;g__Bradyrhizobium;s__	N/A	N/A
MAGs.BN19_2.Bin14	d__Bacteria;p__Firmicutes;c__Bacilli;o__Lactobacillales;f__Lactobacillaceae;g__Limosilactobacillus;s__	N/A	N/A
MAGs.BN19_2.Bin15	d__Bacteria;p__Actinobacteriota;c__Actinomycetia;o__Mycobacteriales;f__Mycobacteriaceae;g__Mycobacterium;s__Mycobacterium aubagnense	GCF_010730955.1	95.52
MAGs.BN19_2.Bin16	d__Bacteria;p__Proteobacteria;c__Alphaproteobacteria;o__Rhizobiales;f__Beijerinckiaceae;g__Methylobacterium;s__Methylobacterium sp002778835	GCA_002778835.1	99.85
MAGs.BN19_2.Bin20	d__Bacteria;p__Firmicutes;c__Bacilli;o__Lactobacillales;f__Lactobacillaceae;g__Lactobacillus;s__Lactobacillus amylovorus	GCF_002706375.1	98.04
MAGs.BN19_2.Bin21	d__Bacteria;p__Bacteroidota;c__Bacteroidia;o__Bacteroidales;f__Bacteroidaceae;g__Prevotella;s__Prevotella sp002300055	GCA_002300055.1	98.86
MAGs.BN19_2.Bin22	d__Bacteria;p__Firmicutes;c__Bacilli;o__Bacillales_B;f__Bacillaceae_C;g__Weizmannia;s_	GCF_000290615.1	98.62

	_Weizmannia coagulans		
MAGs.BN19_2.Bin25	d__Bacteria;p__Myxococcota;c__Polyangia;o__Polyangiales;f__Polyangiaceae;g__Labilithrix;s__	N/A	N/A
MAGs.BN19_2.Bin26	d__Bacteria;p__Firmicutes;c__Bacilli;o__Bacillales_D;f__Amphibacillaceae;g__;s__	N/A	N/A
MAGs.BN19_2.Bin30	d__Bacteria;p__Proteobacteria;c__Gammaproteobacteria;o__Burkholderiales;f__Burkholderiaceae;g__Variovorax;s__Variovorax paradoxus_A	GCF_000382045.1	98.48
MAGs.BN19_2.Bin31	d__Bacteria;p__Bacteroidota;c__Bacteroidia;o__Chitinophagales;f__Chitinophagaceae;g__Sediminibacterium;s__	N/A	N/A
MAGs.BN19_2.Bin32	d__Bacteria;p__Firmicutes_C;c__Negativicutes;o__Veillonellales;f__Megasphaeraceae;g__Megasphaera;s__Megasphaera elsdenii	GCF_003010495.1	98.13
MAGs.BN19_2.Bin34	d__Bacteria;p__Bacteroidota;c__Bacteroidia;o__Bacteroidales;f__Muribaculaceae;g__CAG-873;s__CAG-873 sp001701165	GCA_001701165.1	98.68
MAGs.BN19_2.Bin35	d__Bacteria;p__Proteobacteria;c__Alphaproteobacteria;o__Rhizobiales;f__Xanthobacteraceae;g__Afipia;s__	N/A	N/A
MAGs.BN19_2.Bin37	d__Bacteria;p__Proteobacteria;c__Alphaproteobacteria;o__Acetobacterales;f__Acetobacteraceae;g__Acetobacter;s__Acetobacter pomorum	GCF_002738225.1	95.78
MAGs.BN19_2.Bin38	d__Bacteria;p__Firmicutes;c__Bacilli;o__Lactobacillales;f__Lactobacillaceae;g__Liquorilactobacillus;s__Liquorilactobacillus vini	GCF_000255495.1	98.75
MAGs.BN19_2.Bin39	d__Bacteria;p__Proteobacteria;c__Gammaproteobacteria;o__Xanthomonadales;f__Rhodanobacteraceae;g__Rhodanobacter;s__Rhodanobacter sp001617365	GCF_001617365.1	97.14
MAGs.BN19_2.Bin40	d__Bacteria;p__Proteobacteria;c__Alphaproteobacteria;o__Rhizobiales;f__Beijerinckiaceae;g__Methylovirgula;s__	N/A	N/A
MAGs.BN19_2.Bin42	d__Bacteria;p__Actinobacteriota;c__Actinomycetia;o__Actinomycetales;f__Bifidobacteriaceae;g__Bifidobacterium;s__Bifidobacterium thermophilum	GCF_000771265.1	95.3
MAGs.BN19_2.Bin5	d__Bacteria;p__Bacteroidota;c__Bacteroidia;o__Bacteroidales;f__Bacteroidaceae;g__UBA4372;s__UBA4372 sp900766785	GCA_900766785.1	97.38

MAGs.BN19_2.Bin7	d__Bacteria;p__Firmicutes;c__Bacilli;o__Lactobacillales;f__Lactobacillaceae;g__Limosilactobacillus;s__Limosilactobacillus panis	GCF_001435935.1	99.06
MAGs.BN19_2.Bin8	d__Bacteria;p__Bacteroidota;c__Bacteroidia;o__Bacteroidales;f__Muribaculaceae;g__SFTJ01;s__SFTJ01 sp004557385	GCA_004557385.1	96.87
MAGs.BN19_3.Bin10	d__Bacteria;p__Firmicutes;c__Bacilli;o__Lactobacillales;f__Lactobacillaceae;g__Acetilactobacillus;s__Acetilactobacillus jinshanensis	GCF_004359375.1	99.97
MAGs.BN19_3.Bin11	d__Bacteria;p__Firmicutes;c__Bacilli;o__Lactobacillales;f__Lactobacillaceae;g__Limosilactobacillus;s__	N/A	N/A
MAGs.BN19_3.Bin13	d__Bacteria;p__Bacteroidota;c__Bacteroidia;o__Chitinophagales;f__Chitinophagaceae;g__Sediminibacterium;s__Sediminibacterium magnilacihabitans	GCA_002973575.1	98.1
MAGs.BN19_3.Bin14	d__Bacteria;p__Firmicutes;c__Bacilli;o__Lactobacillales;f__Lactobacillaceae;g__Lactobacillus;s__Lactobacillus amylovorus	GCF_002706375.1	98.5
MAGs.BN19_3.Bin15	d__Bacteria;p__Myxococcota;c__Polyangia;o__Polyangiales;f__Polyangiaceae;g__Labilithrix;s__	N/A	N/A
MAGs.BN19_3.Bin16	d__Bacteria;p__Firmicutes;c__Bacilli;o__Bacillales_B;f__Caldibacillaceae;g__Bacillus_J;s__Bacillus_J thermoamylovorans	GCF_000751775.1	98.31
MAGs.BN19_3.Bin2	d__Bacteria;p__Actinobacteriota;c__Actinomycetia;o__Actinomycetales;f__Bifidobacteriaceae;g__Bifidobacterium;s__Bifidobacterium thermophilum	GCF_000771265.1	95.38
MAGs.BN19_3.Bin20	d__Bacteria;p__Firmicutes;c__Bacilli;o__Bacillales_B;f__Bacillaceae_C;g__Weizmannia;s__Weizmannia coagulans	GCF_000290615.1	98.71
MAGs.BN19_3.Bin21	d__Bacteria;p__Firmicutes;c__Bacilli;o__Bacillales_D;f__Amphibacillaceae;g__;s__	N/A	N/A
MAGs.BN19_3.Bin23	d__Bacteria;p__Firmicutes;c__Bacilli;o__Lactobacillales;f__Lactobacillaceae;g__Lactobacillus;s__Lactobacillus acetotolerans	GCF_001436775.1	99.2
MAGs.BN19_3.Bin24	d__Bacteria;p__Proteobacteria;c__Alphaproteobacteria;o__Rhizobiales;f__Beijerinckiaceae;g__Methylovirgula;s__	N/A	N/A
MAGs.BN19_3.Bin26	d__Bacteria;p__Actinobacteriota;c__Actinomycetia;o__Mycobacteriales;f__Mycobacteriacea	GCF_010730955.1	95.61

	e;g__Mycobacterium;s__Mycobacterium aubagnense		
MAGs.BN19_3.Bin3	d__Bacteria;p__Proteobacteria;c__Alphaproteobacteria;o__Rhizobiales;f__Xanthobacteraceae;g__Bradyrhizobium;s__	N/A	N/A
MAGs.BN19_3.Bin7	d__Bacteria;p__Proteobacteria;c__Alphaproteobacteria;o__Rhizobiales;f__Xanthobacteraceae;g__Afipia;s__	N/A	N/A
MAGs.BN19_3.Bin8	d__Bacteria;p__Firmicutes_C;c__Negativicutes;o__Veillonellales;f__Megasphaeraceae;g__Megasphaera;s__Megasphaera elsdenii	GCF_003010495.1	98.18
MAGs.BN19_3.Bin9	d__Bacteria;p__Proteobacteria;c__Alphaproteobacteria;o__Acetobacterales;f__Acetobacteraceae;g__Acetobacter;s__Acetobacter pomorum	GCF_002738225.1	95.79
MAGs.BN21_1.Bin1	d__Bacteria;p__Firmicutes;c__Bacilli;o__Bacillales_D;f__Amphibacillaceae;g__;s__	N/A	N/A
MAGs.BN21_1.Bin11	d__Bacteria;p__Bacteroidota;c__Bacteroidia;o__Chitinophagales;f__Chitinophagaceae;g__Sediminibacterium;s__Sediminibacterium magnilacihabitans	GCA_002973575.1	98.12
MAGs.BN21_1.Bin12	d__Bacteria;p__Firmicutes;c__Bacilli;o__Bacillales_B;f__Caldibacillaceae;g__Bacillus_J;s__Bacillus_J thermoamylovorans	GCF_000751775.1	98.48
MAGs.BN21_1.Bin15	d__Bacteria;p__Firmicutes;c__Bacilli;o__Bacillales_B;f__Bacillaceae_C;g__Heyndrickxia;s__Heyndrickxia sporothermodurans	GCF_003055085.1	97.97
MAGs.BN21_1.Bin16	d__Bacteria;p__Firmicutes;c__Bacilli;o__Lactobacillales;f__Lactobacillaceae;g__Liquorilactobacillus;s__Liquorilactobacillus vini	GCF_000255495.1	98.81
MAGs.BN21_1.Bin17	d__Bacteria;p__Proteobacteria;c__Alphaproteobacteria;o__Rhizobiales;f__Xanthobacteraceae;g__Afipia;s__	N/A	N/A
MAGs.BN21_1.Bin18	d__Bacteria;p__Proteobacteria;c__Alphaproteobacteria;o__Rhizobiales;f__Beijerinckiaceae;g__Methylovirgula;s__	N/A	N/A
MAGs.BN21_1.Bin19	d__Bacteria;p__Actinobacteriota;c__Actinomycetia;o__Actinomycetales;f__Bifidobacteriaceae;g__Bifidobacterium;s__Bifidobacterium thermophilum	GCF_000771265.1	95.32
MAGs.BN21_1.Bin2	d__Bacteria;p__Firmicutes;c__Bacilli;o__Lactobacillales;f__Lactobacillaceae;g__Limosilactobacillus;s__Limosilactobacillus panis	GCF_001435935.1	99.02

MAGs.BN21_1.Bin21	d__Bacteria;p__Firmicutes;c__Bacilli;o__Bacillales_B;f__Bacillaceae_C;g__Weizmannia;s__Weizmannia coagulans	GCF_000290615.1	98.67
MAGs.BN21_1.Bin23	d__Bacteria;p__Proteobacteria;c__Alphaproteobacteria;o__Acetobacterales;f__Acetobacteraceae;g__Acetobacter;s__Acetobacter pomorum	GCF_002738225.1	95.8
MAGs.BN21_1.Bin25	d__Bacteria;p__Firmicutes;c__Bacilli;o__Lactobacillales;f__Lactobacillaceae;g__Limosilactobacillus;s__	N/A	N/A
MAGs.BN21_1.Bin26	d__Bacteria;p__Proteobacteria;c__Alphaproteobacteria;o__Rhizobiales;f__Xanthobacteraceae;g__Bradyrhizobium;s__	N/A	N/A
MAGs.BN21_1.Bin5	d__Bacteria;p__Firmicutes;c__Bacilli;o__Lactobacillales;f__Lactobacillaceae;g__Acetilactobacillus;s__Acetilactobacillus jinshanensis	GCF_004359375.1	99.96
MAGs.BN21_1.Bin6	d__Bacteria;p__Actinobacteriota;c__Actinomycetia;o__Mycobacteriales;f__Mycobacteriaceae;g__Mycobacterium;s__Mycobacterium aubagnense	GCF_010730955.1	95.45
MAGs.BN21_1.Bin7	d__Bacteria;p__Firmicutes;c__Bacilli;o__Lactobacillales;f__Lactobacillaceae;g__Lactobacillus;s__Lactobacillus amylovorus	GCF_002706375.1	98.57
MAGs.BN21_1.Bin8	d__Bacteria;p__Firmicutes;c__Bacilli;o__Paenibacillales;f__Paenibacillaceae;g__Paenibacillus;s__Paenibacillus sp000787385	GCF_000787385.1	98.8
MAGs.BN21_1.Bin9	d__Bacteria;p__Firmicutes;c__Bacilli;o__Lactobacillales;f__Lactobacillaceae;g__Lactobacillus;s__Lactobacillus acetotolerans	GCF_001436775.1	99.16
MAGs.BN21_2.Bin10	d__Bacteria;p__Proteobacteria;c__Alphaproteobacteria;o__Acetobacterales;f__Acetobacteraceae;g__Acetobacter;s__Acetobacter pomorum	GCF_002738225.1	95.94
MAGs.BN21_2.Bin14	d__Bacteria;p__Firmicutes;c__Bacilli;o__Lactobacillales;f__Lactobacillaceae;g__Lactobacillus;s__	N/A	N/A
MAGs.BN21_2.Bin17	d__Bacteria;p__Proteobacteria;c__Alphaproteobacteria;o__Rhizobiales;f__Xanthobacteraceae;g__Afipia;s__	N/A	N/A
MAGs.BN21_2.Bin18	d__Bacteria;p__Firmicutes;c__Bacilli;o__Lactobacillales;f__Lactobacillaceae;g__Limosilactobacillus;s__Limosilactobacillus panis	GCF_001435935.1	99.2

MAGs.BN21_2.Bin19	d__Bacteria;p__Firmicutes;c__Bacilli;o__Lactobacillales;f__Lactobacillaceae;g__Lactobacillus;s__Lactobacillus acetotolerans	GCF_001436775.1	99.06
MAGs.BN21_2.Bin2	d__Bacteria;p__Bacteroidota;c__Bacteroidia;o__Chitinophagales;f__Chitinophagaceae;g__Sediminibacterium;s__Sediminibacterium magnilacihabitans	GCA_002973575.1	98.11
MAGs.BN21_2.Bin21	d__Bacteria;p__Firmicutes;c__Bacilli;o__Bacillales_B;f__Bacillaceae_C;g__Weizmannia;s__Weizmannia coagulans	GCF_000290615.1	98.66
MAGs.BN21_2.Bin22	d__Bacteria;p__Proteobacteria;c__Alphaproteobacteria;o__Rhizobiales;f__Beijerinckiaceae;g__Methylovirgula;s__	N/A	N/A
MAGs.BN21_2.Bin24	d__Bacteria;p__Actinobacteriota;c__Actinomycetia;o__Actinomycetales;f__Bifidobacteriaceae;g__Bifidobacterium;s__Bifidobacterium thermophilum	GCF_000771265.1	95.36
MAGs.BN21_2.Bin26	d__Bacteria;p__Firmicutes;c__Bacilli;o__Lactobacillales;f__Lactobacillaceae;g__Lactobacillus;s__Lactobacillus amylovorus	GCF_002706375.1	98.6
MAGs.BN21_2.Bin27	d__Bacteria;p__Actinobacteriota;c__Actinomycetia;o__Mycobacteriales;f__Mycobacteriaceae;g__Mycobacterium;s__Mycobacterium aubagnense	GCF_010730955.1	95.47
MAGs.BN21_2.Bin28	d__Bacteria;p__Proteobacteria;c__Alphaproteobacteria;o__Rhizobiales;f__Xanthobacteraceae;g__Bradyrhizobium;s__	N/A	N/A
MAGs.BN21_2.Bin29	d__Bacteria;p__Myxococcota;c__Polyangia;o__Polyangiales;f__Polyangiaceae;g__Labilithrix;s__	N/A	N/A
MAGs.BN21_2.Bin4	d__Bacteria;p__Firmicutes;c__Bacilli;o__Lactobacillales;f__Lactobacillaceae;g__Acetilactobacillus;s__Acetilactobacillus jinshanensis	GCF_004359375.1	99.97
MAGs.BN21_2.Bin5	d__Bacteria;p__Proteobacteria;c__Alphaproteobacteria;o__Reyranellales;f__Reyranellaceae;g__Reyranella;s__	N/A	N/A
MAGs.BN21_2.Bin6	d__Bacteria;p__Firmicutes;c__Bacilli;o__Bacillales_D;f__Amphibacillaceae;g__;s__	N/A	N/A
MAGs.BN21_2.Bin8	d__Bacteria;p__Firmicutes;c__Bacilli;o__Lactobacillales;f__Lactobacillaceae;g__Limosilactobacillus;s__	N/A	N/A
MAGs.BN21_3.Bin10	d__Bacteria;p__Firmicutes;c__Bacilli;o__Lactobacillales;f__Lactobacillaceae;g__Acetilactob	GCF_004359375.1	99.97

	acillus;s__Acetilactobacillus jinshanensis		
MAGs.BN21_3.Bin11	d__Bacteria;p__Firmicutes;c__Bacilli;o__Lactobacillales;f__Lactobacillaceae;g__Limosilactobacillus;s__	N/A	N/A
MAGs.BN21_3.Bin13	d__Bacteria;p__Proteobacteria;c__Alphaproteobacteria;o__Reyranellales;f__Reyranellaceae;g__Reyranella;s__	N/A	N/A
MAGs.BN21_3.Bin15	d__Bacteria;p__Firmicutes;c__Bacilli;o__Bacillales_D;f__Amphibacillaceae;g__;s__	N/A	N/A
MAGs.BN21_3.Bin17	d__Bacteria;p__Firmicutes;c__Bacilli;o__Lactobacillales;f__Lactobacillaceae;g__Lactobacillus;s__Lactobacillus acetotolerans	GCF_001436775.1	99.03
MAGs.BN21_3.Bin18	d__Bacteria;p__Proteobacteria;c__Alphaproteobacteria;o__Rhizobiales;f__Xanthobacteraceae;g__Afipia;s__	N/A	N/A
MAGs.BN21_3.Bin19	d__Bacteria;p__Firmicutes;c__Bacilli;o__Lactobacillales;f__Lactobacillaceae;g__Lactobacillus;s__Lactobacillus amylovorus	GCF_002706375.1	98.73
MAGs.BN21_3.Bin2	d__Bacteria;p__Firmicutes;c__Bacilli;o__Lactobacillales;f__Lactobacillaceae;g__Limosilactobacillus;s__	N/A	N/A
MAGs.BN21_3.Bin20	d__Bacteria;p__Firmicutes;c__Bacilli;o__Paenibacillales;f__Paenibacillaceae;g__Paenibacillus;s__Paenibacillus sp000787385	GCF_000787385.1	98.86
MAGs.BN21_3.Bin22	d__Bacteria;p__Firmicutes;c__Bacilli;o__Bacillales_B;f__Caldibacillaceae;g__Bacillus_J;s__Bacillus_J thermoamylovorans	GCF_000751775.1	98.45
MAGs.BN21_3.Bin23	d__Bacteria;p__Firmicutes;c__Bacilli;o__Bacillales_B;f__Bacillaceae_C;g__Weizmannia;s__Weizmannia coagulans	GCF_000290615.1	98.62
MAGs.BN21_3.Bin25	d__Bacteria;p__Proteobacteria;c__Alphaproteobacteria;o__Rhizobiales;f__Beijerinckiaceae;g__Methylovirgula;s__	N/A	N/A
MAGs.BN21_3.Bin26	d__Bacteria;p__Firmicutes;c__Bacilli;o__Lactobacillales;f__Lactobacillaceae;g__Limosilactobacillus;s__	N/A	N/A
MAGs.BN21_3.Bin27	d__Bacteria;p__Actinobacteriota;c__Actinomycetia;o__Mycobacteriales;f__Mycobacteriaceae;g__Mycobacterium;s__Mycobacterium aubagnense	GCF_010730955.1	95.5

MAGs.BN21_3.Bin3	d__Bacteria;p__Myxococcota;c__Polyangia;o__Polyangiales;f__Polyangiaceae;g__Labilithrix;s__	N/A	N/A
MAGs.BN21_3.Bin4	d__Bacteria;p__Proteobacteria;c__Alphaproteobacteria;o__Rhizobiales;f__Xanthobacteraceae;g__Bradyrhizobium;s__	N/A	N/A
MAGs.BN21_3.Bin5	d__Bacteria;p__Firmicutes;c__Bacilli;o__Lactobacillales;f__Lactobacillaceae;g__Lactobacillus;s__	N/A	N/A
MAGs.BN21_3.Bin6	d__Bacteria;p__Firmicutes;c__Bacilli;o__Lactobacillales;f__Lactobacillaceae;g__Limosilactobacillus;s__Limosilactobacillus panis	GCF_001435935.1	99.14
MAGs.BN21_3.Bin7	d__Bacteria;p__Firmicutes;c__Bacilli;o__Bacillales_B;f__Bacillaceae_C;g__Heyndrickxia;s__Heyndrickxia sporothermodurans	GCF_003055085.1	98.01
MAGs.BN21_3.Bin9	d__Bacteria;p__Bacteroidota;c__Bacteroidia;o__Chitinophagales;f__Chitinophagaceae;g__Sediminibacterium;s__Sediminibacterium magnilacihabitans	GCA_002973575.1	98.12
MAGs.BN25_1.Bin1	d__Bacteria;p__Bacteroidota;c__Bacteroidia;o__Chitinophagales;f__Chitinophagaceae;g__Sediminibacterium;s__Sediminibacterium magnilacihabitans	GCA_002973575.1	98.11
MAGs.BN25_1.Bin10	d__Bacteria;p__Actinobacteriota;c__Actinomycetia;o__Mycobacteriales;f__Mycobacteriaceae;g__Mycobacterium;s__Mycobacterium aubagnense	GCF_010730955.1	95.49
MAGs.BN25_1.Bin12	d__Bacteria;p__Proteobacteria;c__Alphaproteobacteria;o__Rhizobiales;f__Rhizobiaceae;g__Mesorhizobium;s__Mesorhizobium terrae	GCF_008727715.1	99.86
MAGs.BN25_1.Bin14	d__Bacteria;p__Bacteroidota;c__Bacteroidia;o__Chitinophagales;f__Chitinophagaceae;g__Sediminibacterium;s__	N/A	N/A
MAGs.BN25_1.Bin16	d__Bacteria;p__Proteobacteria;c__Alphaproteobacteria;o__Reyranellales;f__Reyranellaceae;g__Reyranella;s__	N/A	N/A
MAGs.BN25_1.Bin18	d__Bacteria;p__Firmicutes;c__Bacilli;o__Bacillales_B;f__Caldibacillaceae;g__Bacillus_J;s__Bacillus_J thermoamylovorans	GCF_000751775.1	98.25
MAGs.BN25_1.Bin19	d__Bacteria;p__Deinococcota;c__Deinococci;o__Deinococcales;f__Thermaceae;g__Meiothermus;s__	N/A	N/A

MAGs.BN25_1.Bin20	d__Bacteria;p__Bacteroidota;c__Bacteroidia;o__Chitinophagales;f__Chitinophagaceae;g__Chitinophaga;s__	N/A	N/A
MAGs.BN25_1.Bin21	d__Bacteria;p__Firmicutes;c__Bacilli;o__Lactobacillales;f__Lactobacillaceae;g__Lactobacillus;s__Lactobacillus acetotolerans	GCF_001436775.1	99.1
MAGs.BN25_1.Bin23	d__Bacteria;p__Myxococcota;c__Polyangia;o__Polyangiales;f__Polyangiaceae;g__Labilithrix;s__	N/A	N/A
MAGs.BN25_1.Bin24	d__Bacteria;p__Firmicutes;c__Bacilli;o__Lactobacillales;f__Lactobacillaceae;g__Limosilactobacillus;s__	N/A	N/A
MAGs.BN25_1.Bin25	d__Bacteria;p__Firmicutes;c__Bacilli;o__Paenibacillales;f__Paenibacillaceae;g__Paenibacillus;s__Paenibacillus sp000787385	GCF_000787385.1	98.88
MAGs.BN25_1.Bin26	d__Bacteria;p__Firmicutes;c__Bacilli;o__Lactobacillales;f__Lactobacillaceae;g__Acetilactobacillus;s__Acetilactobacillus jinshanensis	GCF_004359375.1	99.97
MAGs.BN25_1.Bin27	d__Bacteria;p__Firmicutes;c__Bacilli;o__Bacillales_B;f__Bacillaceae_C;g__Heyndrickxia;s__Heyndrickxia sporothermodurans	GCF_003055085.1	98.12
MAGs.BN25_1.Bin29	d__Bacteria;p__Proteobacteria;c__Alphaproteobacteria;o__Acetobacterales;f__Acetobacteraceae;g__Acetobacter;s__Acetobacter pomorum	GCF_002738225.1	95.8
MAGs.BN25_1.Bin3	d__Bacteria;p__Firmicutes;c__Bacilli;o__Bacillales_B;f__Bacillaceae_C;g__Weizmannia;s__Weizmannia coagulans	GCF_000290615.1	98.61
MAGs.BN25_1.Bin32	d__Bacteria;p__Proteobacteria;c__Alphaproteobacteria;o__Rhizobiales;f__Xanthobacteraceae;g__Z2-YC6860;s__	N/A	N/A
MAGs.BN25_1.Bin33	d__Bacteria;p__Firmicutes;c__Bacilli;o__;f__;g__;s__	N/A	N/A
MAGs.BN25_1.Bin4	d__Bacteria;p__Firmicutes;c__Bacilli;o__Bacillales_D;f__Amphibacillaceae;g__;s__	N/A	N/A
MAGs.BN25_1.Bin5	d__Bacteria;p__Proteobacteria;c__Alphaproteobacteria;o__Rhizobiales;f__Xanthobacteraceae;g__Bradyrhizobium;s__	N/A	N/A
MAGs.BN25_1.Bin6	d__Bacteria;p__Proteobacteria;c__Alphaproteobacteria;o__Rhizobiales;f__Beijerinckiaceae;g__Methylovirgula;s__	N/A	N/A

MAGs.BN25_1.Bin8	d__Bacteria;p__Firmicutes;c__Bacilli;o__Lactobacillales;f__Lactobacillaceae;g__Lactobacillus;s__Lactobacillus amylovorus	GCF_002706375.1	98.62
MAGs.BN25_1.Bin9	d__Bacteria;p__Proteobacteria;c__Alphaproteobacteria;o__Rhizobiales;f__Xanthobacteraceae;g__Afipia;s__	N/A	N/A
MAGs.BN25_2.Bin11	d__Bacteria;p__Firmicutes;c__Bacilli;o__Paenibacillales;f__Paenibacillaceae;g__Paenibacillus;s__Paenibacillus sp000787385	GCF_000787385.1	98.9
MAGs.BN25_2.Bin12	d__Bacteria;p__Firmicutes;c__Bacilli;o__Bacillales_B;f__Bacillaceae_C;g__Weizmannia;s__Weizmannia coagulans	GCF_000290615.1	98.64
MAGs.BN25_2.Bin14	d__Bacteria;p__Proteobacteria;c__Alphaproteobacteria;o__Rhizobiales;f__Xanthobacteraceae;g__Z2-YC6860;s__	N/A	N/A
MAGs.BN25_2.Bin15	d__Bacteria;p__Firmicutes;c__Bacilli;o__Lactobacillales;f__Lactobacillaceae;g__Lactobacillus;s__Lactobacillus amylovorus	GCF_002706375.1	98.85
MAGs.BN25_2.Bin16	d__Bacteria;p__Firmicutes;c__Bacilli;o__Bacillales_B;f__Caldibacillaceae;g__Bacillus_J;s__Bacillus_J thermoamylovorans	GCF_000751775.1	98.41
MAGs.BN25_2.Bin18	d__Bacteria;p__Bacteroidota;c__Bacteroidia;o__Chitinophagales;f__Chitinophagaceae;g__Sediminibacterium;s__	N/A	N/A
MAGs.BN25_2.Bin19	d__Bacteria;p__Proteobacteria;c__Alphaproteobacteria;o__Acetobacterales;f__Acetobacteraceae;g__Acetobacter;s__Acetobacter pomorum	GCF_002738225.1	95.99
MAGs.BN25_2.Bin2	d__Bacteria;p__Proteobacteria;c__Alphaproteobacteria;o__Rhizobiales;f__Xanthobacteraceae;g__Bradyrhizobium;s__	N/A	N/A
MAGs.BN25_2.Bin21	d__Bacteria;p__Firmicutes;c__Bacilli;o__Lactobacillales;f__Lactobacillaceae;g__Lactobacillus;s__Lactobacillus acetotolerans	GCF_001436775.1	99.14
MAGs.BN25_2.Bin22	d__Bacteria;p__Firmicutes;c__Bacilli;o__Bacillales_D;f__Amphibacillaceae;g__;s__	N/A	N/A
MAGs.BN25_2.Bin23	d__Bacteria;p__Firmicutes;c__Bacilli;o__Bacillales_D;f__Amphibacillaceae;g__Oceanobacillus;s__	N/A	N/A
MAGs.BN25_2.Bin24	d__Bacteria;p__Bacteroidota;c__Bacteroidia;o__Chitinophagales;f__Chitinophagaceae;g__Se	GCA_002973575.1	98.1

	diminibacterium;s__Sediminibacterium magnilacihabitans		
MAGs.BN25_2.Bin25	d__Bacteria;p__Proteobacteria;c__Alphaproteobacteria;o__Rhizobiales;f__Rhizobiaceae;g__ Mesorhizobium;s__Mesorhizobium terrae	GCF_008727715.1	99.79
MAGs.BN25_2.Bin26	d__Bacteria;p__Proteobacteria;c__Alphaproteobacteria;o__Reyranellales;f__Reyranellaceae; g__Reyranella;s__	N/A	N/A
MAGs.BN25_2.Bin30	d__Bacteria;p__Proteobacteria;c__Alphaproteobacteria;o__Rhizobiales;f__Beijerinckiaceae;g __Methylovirgula;s__	N/A	N/A
MAGs.BN25_2.Bin31	d__Bacteria;p__Firmicutes;c__Bacilli;o__Lactobacillales;f__Lactobacillaceae;g__Limosilact obacillus;s__	N/A	N/A
MAGs.BN25_2.Bin32	d__Bacteria;p__Proteobacteria;c__Alphaproteobacteria;o__Rhizobiales;f__Xanthobacteracea e;g__Afipia;s__	N/A	N/A
MAGs.BN25_2.Bin34	d__Bacteria;p__Bacteroidota;c__Bacteroidia;o__Chitinophagales;f__Chitinophagaceae;g__C hitinophaga;s__	N/A	N/A
MAGs.BN25_2.Bin35	d__Bacteria;p__Deinococcota;c__Deinococci;o__Deinococcales;f__Thermaceae;g__Meiothe rmus;s__	N/A	N/A
MAGs.BN25_2.Bin36	d__Bacteria;p__Actinobacteriota;c__Actinomycetia;o__Mycobacteriales;f__Mycobacteriacea e;g__Mycobacterium;s__Mycobacterium aubagnense	GCF_010730955.1	95.47
MAGs.BN25_2.Bin4	d__Bacteria;p__Firmicutes;c__Bacilli;o__;f__;g__;s__	N/A	N/A
MAGs.BN25_2.Bin6	d__Bacteria;p__Proteobacteria;c__Gammaproteobacteria;o__Burkholderiales;f__Burkholderi aceae;g__Ralstonia;s__Ralstonia pickettii	GCF_000743455.1	97.54
MAGs.BN25_2.Bin7	d__Bacteria;p__Myxococcota;c__Polyangia;o__Polyangiales;f__Polyangiaceae;g__Labilithri x;s__	N/A	N/A
MAGs.BN25_2.Bin9	d__Bacteria;p__Firmicutes;c__Bacilli;o__Lactobacillales;f__Lactobacillaceae;g__Acetilactob acillus;s__Acetilactobacillus jinshanensis	GCF_004359375.1	99.96
MAGs.BN25_3.Bin1	d__Bacteria;p__Proteobacteria;c__Alphaproteobacteria;o__Rhizobiales;f__Beijerinckiaceae;g __Methylovirgula;s__	N/A	N/A

MAGs.BN25_3.Bin10	d__Bacteria;p__Actinobacteriota;c__Actinomycetia;o__Mycobacteriales;f__Mycobacteriaceae;g__Mycobacterium;s__Mycobacterium aubagnense	GCF_010730955.1	95.49
MAGs.BN25_3.Bin12	d__Bacteria;p__Myxococcota;c__Polyangia;o__Polyangiales;f__Polyangiaceae;g__Labilithrix;s__	N/A	N/A
MAGs.BN25_3.Bin13	d__Bacteria;p__Firmicutes;c__Bacilli;o__Thermicanales;f__Thermicanaceae;g__;s__	N/A	N/A
MAGs.BN25_3.Bin14	d__Bacteria;p__Proteobacteria;c__Alphaproteobacteria;o__Reyranellales;f__Reyranellaceae;g__Reyranella;s__	N/A	N/A
MAGs.BN25_3.Bin15	d__Bacteria;p__Bacteroidota;c__Bacteroidia;o__Chitinophagales;f__Chitinophagaceae;g__Sediminibacterium;s__	N/A	N/A
MAGs.BN25_3.Bin16	d__Bacteria;p__Deinococcota;c__Deinococci;o__Deinococcales;f__Thermaceae;g__Meiothermus;s__	N/A	N/A
MAGs.BN25_3.Bin17	d__Bacteria;p__Proteobacteria;c__Alphaproteobacteria;o__Rhizobiales;f__Xanthobacteraceae;g__Bradyrhizobium;s__	N/A	N/A
MAGs.BN25_3.Bin2	d__Bacteria;p__Proteobacteria;c__Alphaproteobacteria;o__Rhizobiales;f__Xanthobacteraceae;g__Z2-YC6860;s__	N/A	N/A
MAGs.BN25_3.Bin20	d__Bacteria;p__Proteobacteria;c__Alphaproteobacteria;o__Acetobacterales;f__Acetobacteraceae;g__Acetobacter;s__Acetobacter pomorum	GCF_002738225.1	95.81
MAGs.BN25_3.Bin21	d__Bacteria;p__Bacteroidota;c__Bacteroidia;o__Chitinophagales;f__Chitinophagaceae;g__Chitinophaga;s__	N/A	N/A
MAGs.BN25_3.Bin24	d__Bacteria;p__Proteobacteria;c__Gammaproteobacteria;o__Burkholderiales;f__Burkholderiaceae;g__Ralstonia;s__Ralstonia pickettii	GCF_000743455.1	97.52
MAGs.BN25_3.Bin25	d__Bacteria;p__Firmicutes;c__Bacilli;o__Bacillales_B;f__Bacillaceae_C;g__Heyndrickxia;s__Heyndrickxia sporothermodurans	GCF_003055085.1	98.12
MAGs.BN25_3.Bin28	d__Bacteria;p__Firmicutes;c__Bacilli;o__Bacillales_D;f__Amphibacillaceae;g__;s__	N/A	N/A
MAGs.BN25_3.Bin30	d__Bacteria;p__Firmicutes;c__Bacilli;o__Lactobacillales;f__Lactobacillaceae;g__Lactobacillus;s__Lactobacillus acetotolerans	GCF_001436775.1	99.18

MAGs.BN25_3.Bin31	d__Bacteria;p__Proteobacteria;c__Alphaproteobacteria;o__Rhizobiales;f__Rhizobiaceae;g__Mesorhizobium;s__Mesorhizobium terrae	GCF_008727715.1	99.77
MAGs.BN25_3.Bin33	d__Bacteria;p__Firmicutes;c__Bacilli;o__Lactobacillales;f__Lactobacillaceae;g__Lactobacillus;s__Lactobacillus amylovorus	GCF_002706375.1	98.5
MAGs.BN25_3.Bin4	d__Bacteria;p__Firmicutes;c__Bacilli;o__Bacillales_B;f__Caldibacillaceae;g__Bacillus_J;s__Bacillus_J thermoamylovorans	GCF_000751775.1	98.27
MAGs.BN25_3.Bin5	d__Bacteria;p__Firmicutes;c__Bacilli;o__Lactobacillales;f__Lactobacillaceae;g__Acetilactobacillus;s__Acetilactobacillus jinshanensis	GCF_004359375.1	99.97
MAGs.BN25_3.Bin6	d__Bacteria;p__Firmicutes;c__Bacilli;o__Lactobacillales;f__Lactobacillaceae;g__Limosilactobacillus;s__	N/A	N/A
MAGs.BN25_3.Bin7	d__Bacteria;p__Bacteroidota;c__Bacteroidia;o__Chitinophagales;f__Chitinophagaceae;g__Sediminibacterium;s__Sediminibacterium magnilacihabitans	GCA_002973575.1	98.11
MAGs.BN25_3.Bin8	d__Bacteria;p__Proteobacteria;c__Alphaproteobacteria;o__Rhizobiales;f__Xanthobacteraceae;g__Afipia;s__	N/A	N/A
MAGs.BN25_3.Bin9	d__Bacteria;p__Firmicutes;c__Bacilli;o__Bacillales_B;f__Bacillaceae_C;g__Weizmannia;s__Weizmannia coagulans	GCF_000290615.1	98.57
