# Supplementary material for: Metagenomics and Metagenome-Assembled Genomes: Analysis of Cupei from Sichuan Baoning Vinegar, One of the Four Traditional Renowned Vinegars in China
Source: Foods. 2025 Jan 26;14(3):398. doi: 10.3390/foods14030398 (PMC11816609; doi:10.3390/foods14030398)
Supplement: Supplementary file 1 [file foods-14-00398-s001.zip › Table S4.pdf]

Table S4 Prediction of secondary metabolite biosynthetic gene clusters from the MAGs using antiSMASH.

MAG	MAG	Contig	Region	Start (bp)	Stop (bp)	Type
BN01_1.10	*Serratia rubidaea*	BN01_1__8794	26.1	1	13849	NRPS-like
		BN01_1__14401	42.1	18916	31030	butyrolactone
		BN01_1__61795	189.1	1	5427	NI-siderophore
		BN01_1__86954	251.1	1	7055	NRPS
		BN01_1__90988	269.1	1	16470	arylpolyene
		BN01_1__105187	317.1	1	16122	betalactone
		BN01_1__107685	323.1	1	7729	redox-cofactor
		BN01_1__130603	384.1	1	3548	NI-siderophore
		BN01_1__141656	410.1	1	6726	NRPS
		BN01_1__151081	431.1	1	6274	RiPP-like
		BN01_1__156630	444.1	1	3843	RiPP-like
		BN01_1__163899	461.1	1	18554	NRPS
BN01_1.31	*Acetobacter lovaniensis*	BN01_1__1305	3.1	1	3998	arylpolyene
		BN01_1__14220	18.1	1968	19211	terpene
		BN01_1__15801	22.1	1	3346	arylpolyene
		BN01_1__36140	50.1	1	6255	arylpolyene
		BN01_1__62504	83.1	10787	20899	RiPP-like
		BN01_1__73348	101.1	1	6524	terpene
		BN01_1__73348	108.1	1	10298	hydrogen-cyanide
		BN01_1__165678	191.1	16938	31026	redox-cofactor

BN01_1.33	*Pediococcus pentosaceus*	BN01_1__140471	161.1	1	23048	T3PKS
		BN01_1__58930	46.1	1	13064	redox-cofactor
BN01_1.41	*Acetobacter syzygii*	BN01_1__89956	65.1	1	14734	terpene
		BN01_1__97400	71.1	1	18028	terpene
		BN01_1__32416	36.1	3365	24183	terpene
		BN01_1__72233	69.1	13197	33906	cyclic-lactone-autoinducer
		BN01_1__85733	78.1	1	18643	NRPS
		BN01_1__102182	94.1	1	13249	terpene
BN01_1.43	*Staphylococcus gallinarum*	BN01_1__115900	106.1	3724	44893	T3PKS
		BN01_1__132522	120.1	19052	39924	terpene
		BN01_1__133476	123.1	35543	56415	terpene
		BN01_1__134905	125.1	1	14901	cyclic-lactone-autoinducer
BN01_1.59	*Weissella paramesenteroides*	BN01_1__77493	98.1	1	32027	T3PKS
		BN01_2__6798	1.1	4017	15957	redox-cofactor
BN01_2.10	*Acetobacter peroxydans*	BN01_2__10264	2.1	1	6279	terpene
		BN01_2__36615	11.1	1	13664	terpene
BN01_2.16	*Limosilactobacillus* sp.	BN01_2__70879	8.1	1	14372	T3PKS
		BN01_3__14217	36.1	1	2803	terpene
BN01_3.4	*Acetobacter indonesiensis*	BN01_3__32256	82.1	1	12235	arylpolyene
		BN01_3__48921	119.1	1	3357	terpene
BN01_3.10	*Paucilactobacillus suebicus*	BN01_3__26592	2.1	425131	437077	RiPP-like
		BN01_3__71829	11.1	104174	145343	T3PKS
BN01_3.13	*Streptomyces cacaoi*	BN01_3__6386	11.1	1	24978	T1PKS

BN01_3__8022	16.1	1	13171	T1PKS
BN01_3__16814	31.1	1	15267	nucleoside
BN01_3__23825	42.1	1	2972	T1PKS
BN01_3__24366	43.1	4479	48019	NRPS, NRPS-like
BN01_3__32230	55.1	1	2599	T1PKS
BN01_3__32949	57.1	1	12163	terpene
BN01_3__36626	63.1	1	4846	T1PKS
BN01_3__37697	64.1	7428	64010	NRPS
BN01_3__38516	65.1	1	59644	transAT-PKS-like,NRPS-like
BN01_3__40479	68.1	1	46336	T1PKS
BN01_3__50789	87.1	32450	50362	NI-siderophore
BN01_3__56017	98.1	29010	63095	NRP-metallophore,NRPS
BN01_3__63812	113.1	1	42125	NRPS
BN01_3__68719	121.1	24287	33846	ectoine
BN01_3__81775	133.1	1	7349	NRPS
BN01_3__83124	134.1	12664	23874	terpene
BN01_3__86600	144.1	33808	45649	RRE-containing
BN01_3__89958	152.1	15876	26250	ectoine
BN01_3__97726	157.1	8065	40798	NRPS
BN01_3__110722	180.1	964	31733	CDPS,T1PKS
BN01_3__130920	205.1	1	7146	T1PKS
BN01_3__134335	211.1	1	24190	NRPS

BN01_3__138164	218.1	1	3800	NRPS
BN01_3__138701	219.1	1	5786	CDPS
BN01_3__147605	229.1	46801	73434	terpene
BN01_3__163857	253.1	1	27936	NRPS
BN01_3__165895	256.1	1	37270	NRPS
BN01_3__171522	263.1	1	8865	T2PKS
BN01_3__172642	266.1	1	7752	RiPP-like
BN01_3__175019	273.1	1	24225	NRP-metallophore
BN01_3__187166	288.1	1	15830	terpene
BN01_3__190327	291.1	367739	54573	terpene
BN01_3__192446	294.1	1	9483	NRPS
BN01_3__197743	302.1	1	13932	NRPS-like,T1PKS
BN01_3__200467	305.1	1	12463	CDPS
BN01_3__204966	311.1	1	6300	T1PKS
BN01_3__216414	322.1	1	14923	lanthipeptide class III
BN01_3__217080	323.1	1	18741	NI-siderophore
BN01_3__220068	330.1	1	15490	lassopeptide
BN01_3__227555	338.1	1	18225	NRPS,NRP-metallophore,NRPS-like
BN01_3__228590	340.1	1	4233	NRPS
BN01_3__231486	347.1	1	15052	NRPS
BN01_3__231889	348.1	1	31555	NRPS
BN01_3__242619	360.1	1	34746	T1PKS,NRPS

		BN01_3__8571	7.1	1	3972	NRPS
		BN01_3__37499	25.1	1	5941	NRPS
		BN01_3__43182	27.1	1	10049	hydrogen-cyanide
		BN01_3__85772	58.1	1	9784	NRPS
		BN01_3__104333	67.1	1	24588	arylpolyene
		BN01_3__129338	95.1	83332	112544	phosphonate
		BN01_3__145485	107.1	1	12178	hydrogen-cyanide
BN01_3.33	*Burkholderia vietnamiensis*	BN01_3__157534	115.1	1	12183	NRPS
		BN01_3__157534	124.1	1	7167	redox-cofactor
		BN01_3__196766	135.1	1	8268	NRPS
		BN01_3__224196	160.1	1	7223	arylpolyene
		BN01_3__226040	161.1	1	5740	T1PKS,NRPS
		BN01_3__235051	170.1	101902	122807	terpene
		BN01_3__236110	171.1	1	5767	NRPS
		BN01_3__244326	180.1	1	6249	RiPP-like
		BN01_3__86379	27.1	2562	27738	terpene
BN01_3.34	*Acetobacter orientalis*	BN01_3__87672	28.1	1	5235	RiPP-like
		BN01_3__87672	28.2	39364	59948	hserlactone
		BN01_3__155182	47.1	62329	84462	redox-cofactor
		BN01_3__966	2.1	1	11975	redox-cofactor
BN01_3.40	*Acetobacter* sp.	BN01_3__43008	43.1	1	5763	terpene
		BN01_3__78586	83.1	1	2726	terpene
		BN01_3__145679	148.1	1	16762	arylpolyene

		BN01_3__148794	151.1	1	6278	terpene
		BN01_3__30822	5.1	1	34948	terpene
		BN01_3__149351	29.1	40249	96377	indole,T3PKS
BN01_3.55	*Saccharopolyspora rectivirgula*	BN01_3__174683	35.1	18010	38804	terpene
		BN01_3__174683	35.2	107540	128805	terpene
		BN01_3__212084	37.1	119705	145429	redox-cofactor
		BN01_3__214946	38.1	4160	55184	NRP-metallophore,NRPS
		BN01_3__31925	23.1	1	10142	terpene
BN01_3.64	*Lactiplantibacillus plantarum*	BN01_3__39275	29.1	89737	100937	cyclic-lactone-autoinducer
		BN01_3__62406	36.1	83451	124620	T3PKS
		BN05_1__5890	12.1	2198	12581	ectoine
		BN05_1__39514	109.1	1	7644	redox-cofactor
BN05_1.14	*Pseudomonas jilinensis*	BN05_1__41624	115.1	1	9257	betalactone
		BN05_1__46155	129.1	1	5610	betalactone
		BN05_1__110705	28.1	10595	45343	T3PKS
BN05_2.12	*Limosilactobacillus* sp.	BN05_2__15248	25.1	1	17415	T3PKS
BN05_3.9	*Limosilactobacillus* sp.	BN05_3__122830	82.1	1	17973	T3PKS
BN05_3.52	*Limosilactobacillus* sp.	BN05_3__35645	23.1	71873	106919	T3PKS
BN07_1.5	*Weizmannia coagulans*	BN07_1__21049	39.1	1	13629	T3PKS
BN07_1.23	*Limosilactobacillus pontis*	BN07_1__62821	115.1	1	18281	T3PKS
		BN07_2__17522	26.1	1	11315	terpene
BN07_2.30	f__Amphibacillaceae	BN07_2__28299	43.1	106016	147101	T3PKS
		BN07_2__98010	182.1	1	6570	terpene

BN07_2.31	*Limosilactobacillus* sp.	BN07_2__67836	110.1	1	26842	T3PKS
BN07_2.37	*Bacillus thermoamylovorans*	BN07_2__69739	134.1	1	4555	terpene
		BN07_2__101470	197.1	1	5318	betalactone
BN07_3.8	*Furfurilactobacillus rossiae*	BN07_3__48826	33.1	36255	57704	arylpolyene
BN09_1.32	*Prevotella* sp.	BN09_1__89472	227.1	1	21542	NAPAA
BN09_2.16	*Acetobacter ghanensis*	BN09_2__17095	40.1	1	13720	terpene
		BN09_2__18265	44.1	1	8209	terpene
		BN09_2__60890	123.1	1	3363	ranthipeptide
BN09_3.5	*Limosilactobacillus* sp.	BN09_3__61029	3.1	470771	506784	T3PKS
BN09_3.9	*Companilactobacillus* sp.	BN09_3__102026	54.1	46295	56672	RiPP-like
BN09_3.27	*Lactobacillus* sp.	BN09_3__73948	11.1	12848	54014	T3PKS
BN09_3.43	*Limosilactobacillus* sp.	BN09_3__12803	16.1	1	8315	T3PKS
		BN09_3__44870	60.1	2944	12498	RiPP-like
BN09_3.44	*Limosilactobacillus* sp.	BN09_3__36754	89.1	1	15720	T3PKS
BN09_3.45		BN09_3__46551	64.1	1	7808	RiPP-like
BN11_2.32	*Komagataeibacter melaceti*	BN11_2__32051	102.1	1	7166	redox-cofactor
		BN11_2__57666	185.1	1662	23027	terpene
BN13_3.7	*Limosilactobacillus secaliphilus*	BN13_3_62342	88.1	1	7171	T3PKS
BN13_3.16	*Limosilactobacillus* sp.	BN13_3_8117	6.1	10056	51216	T3PKS
BN15_2.32	*Lactobacillus* sp.	BN15_2_87486	9.1	609453	631966	lanthipeptide class III
BN15_2.38	*Methylobacterium* sp.	BN15_2__28096	173.1	1	4744	terpene
		BN15_2__33485	202.1	1	7021	redox-cofactor

		BN15_2__33492	203.1	1	13091	hserlactone
		BN15_2__38540	234.1	1	3843	NRPS-like
		BN15_2__42151	256.1	1	5204	RiPP-like
		BN15_2__53716	339.1	1	3023	terpene
		BN15_2__58657	380.1	2239	14679	terpene
		BN15_2__60565	399.1	1	2637	terpene
BN17_1.5	*Rummeliibacillus* sp.	BN17_1_52352	81.1	1	6798	RiPP-like
		BN17_1_57253	89.1	1	2999	RiPP-like
		BN17_1_81502	126.1	1	5891	RiPP-like
BN17_1.8	*Xanthomonas massiliensis*	BN17_1_15087	40.1	2349	13173	RiPP-like
		BN17_1_75240	174.1	1	14376	arylpolyene
BN17_2.34	*Heyndrickxia sporothermodurans*	BN17_2__62246	103.1	125580	147448	terpene
		BN17_2__84320	148.1	59526	96090	T3PKS
		BN17_2__84388	149.1	8087	30314	redox-cofactor
BN17_3.23	*Oceanobacillus caeni*	BN17_3__22187	68.1	22195	43022	terpene
		BN17_3__59960	168.1	5735	12281	ectoine
		BN17_3__79922	212.1	1	25327	T3PKS
		BN17_3__92977	241.1	1	34287	T3PKS
BN19_1.13	*Lentilactobacillus raoultii*	BN19_1_3002	4.1	1	17209	lanthipeptide class IV
		BN19_1_25649	18.1	5914	35818	T3PKS
		BN19_1_127164	82.1	1	4587	T3PKS
BN19_1.18	*Limosilactobacillus* sp.	BN19_1_107271	33.1	267	41436	T3PKS
BN19_1.33	*Acinetobacter baumannii*	BN19_1__18958	53.1	1	11345	NRP-metallophore

		BN19_1__21049	59.1	1	15607	arylpolyene
		BN19_1__33519	104.1	1	15774	NI-siderophore
		BN19_1__57371	158.1	2439	19827	NI-siderophore
		BN19_1__103006	292.1	1	15314	arylpolyene
		BN19_1__107087	301.1	1	7519	RRE-containing
		BN19_1__121392	352.1	1	12549	NAPAA
		BN19_1__128626	369.1	1	7527	hserlactone
BN19_2.5	f__Bacteroidaceae	BN19_2_94013	150.1	1	12409	RRE-containing
		BN19_2__16283	34.1	1	15091	redox-cofactor
		BN19_2__38971	76.1	1	2621	terpene
		BN19_2__89554	187.1	1	11972	terpene
BN19_2.16	*Methylobacterium* sp	BN19_2__128485	267.1	4435	16045	RiPP-like
		BN19_2__136724	279.1	3892	20398	terpene
		BN19_2__138517	284.1	1	6640	terpene
		BN19_2__159005	330.1	1	35843	T1PKS
BN19_2.21	*Prevotella* sp	BN19_2_87379	159.1	5486	16074	phosphonate
		BN19_2__1301	1.1	1	8145	T1PKS/NRPS-like/NRPS
		BN19_2__4236	7.1	71856	93642	terpene
		BN19_2__11958	19.1	50574	92490	resorcinol
BN19_2.30	*Variovorax paradoxus*	BN19_2__21504	27.1	73266	86140	hydrogen-cyanide
		BN19_2__30236	36.1	56034	108461	T1PKS/NRPS
		BN19_2__38542	43.1	13636	35795	redox-cofactor
		BN19_2__47801	49.1	46304	60088	RiPP-like

		BN19_2__58802	64.1	72288	119811	NRPS
		BN19_2__65331	68.1	1	11099	NRP-metallophore/NRPS
		BN19_2__70372	73.1	7889	40383	arylpolyene/resorcinol
		BN19_2__90163	90.1	66725	104933	arylpolyene
		BN19_2__111254	102.1	88059	108661	hserlactone
		BN19_2__139263	119.1	1	23896	NRPS
		BN19_2__157106	135.1	106460	119408	hydrogen-cyanide
		BN19_2__159102	136.1	1	6069	NRPS
		BN19_2_33690	33.1	52822	63652	RiPP-like
BN19_2.31	*Sediminibacterium* sp.	BN19_2_60247	57.1	1	9845	terpene
		BN19_2_116372	107.1	8754	36154	betalactone
BN19_2.39	*Rhodanobacter* sp	BN19_2_17885	15.1	16997	27863	RiPP-like
		BN19_2_143984	89.1	1	22365	arylpolyene
BN19_2.42	*Bifidobacterium thermophilum*	BN19_2_63527	5.1	37083	57511	phenazine
BN19_3.8	*Megasphaera elsdenii*	BN19_3_87569	67.1	23912	45555	ranthipeptide
		BN19_3__9646	2.1	83092	104806	terpene
		BN19_3__13970	4.1	52016	62879	RiPP-like
		BN19_3__18173	7.1	59763	81811	terpene
		BN19_3__27108	12.1	49469	60308	RiPP-like
BN19_3.24	*Methylovirgula* sp.	BN19_3__76935	26.1	19951	74716	T3PKS/NAPAA
		BN19_3__97075	29.1	105444	127619	redox-cofactor
		BN19_3__98729	30.1	102426	123628	2dos
		BN19_3__99674	32.1	65614	86435	terpene

BN21_2.18	*Limosilactobacillus panis*	BN21_2_27385	37.1	5869	45435	T3PKS
BN21_3.9	*Sediminibacterium magnilacihabitans*	BN21_3__7676	1.1	52272	80564	betalactone
		BN21_3__7676	1.2	94611	104246	RiPP-like
		BN21_3__70642	13.1	32081	42911	RiPP-like
		BN21_3__99551	19.1	14311	35147	terpene
BN21_3.20	*Paenibacillus* sp.	BN21_3__8685	20.1	44358	77982	T3PKS
		BN21_3__35618	92.1	1	12987	RRE-containing
		BN21_3__61481	164.1	1	9990	NRPS
		BN21_3__84349	211.1	1	15791	lanthipeptide class I
		BN21_3__94384	240.1	1	11083	resorcinol
		BN21_3__104573	266.1	8470	31930	LAP/thiopeptide
BN25_1.12	*Mesorhizobium terrae*	BN25_1__15154	111.1	1	38370	phosphonate-like
		BN25_1__21316	160.1	1433	12356	RiPP-like
		BN25_1__63213	467.1	1	21358	NAPAA
		BN25_1__67808	501.1	1	16759	NI-siderophore
		BN25_1__71883	533.1	1	2823	hydrogen-cyanide
		BN25_1__78846	594.1	1	24254	arylpolyene
		BN25_1__92644	698.1	1	7733	hserlactone
		BN25_1__94116	710.1	1	3819	RiPP-like
		BN25_1__101266	759.1	1	9063	NI-siderophore
		BN25_1__102446	767.1	1	4742	hserlactone
		BN25_1__103056	775.1	1	5069	RiPP-like
		BN25_1__111910	850.1	1	14250	arylpolyene

		BN25_1__5012	4.1	171496	209987	betalactone
		BN25_1__24537	18.1	23061	45621	lassopeptide/RRE-containing
		BN25_1__27671	20.1	4015	16791	hydrogen-cyanide
		BN25_1__42348	28.1	97991	108809	RiPP-like
		BN25_1__43214	29.1	20642	31493	RiPP-like
		BN25_1__45771	30.1	117768	165291	T1PKS
		BN25_1__51590	36.1	93281	106136	hydrogen-cyanide
BN25_1.16	*Reyranella* sp.	BN25_1__53584	38.1	53609	67552	hydrogen-cyanide
		BN25_1__57183	43.1	98537	109436	RiPP-like
		BN25_1__57183	43.2	193910	218890	terpene
		BN25_1__70664	53.1	39647	80519	phosphonate-like
		BN25_1__77936	63.1	127963	128796	RiPP-like
		BN25_1__92043	81.1	50480	71667	RRE-containing
		BN25_1__104220	88.1	42111	64282	redox-cofactor
		BN25_1__104220	88.2	161593	182018	phenazine
BN25_1.19	*Meiothermus* sp.	BN25_1_25681	38.1	24895	48716	terpene
		BN25_1_57204	90.1	1	20706	T3PKS
		BN25_1__2446	9.1	1	19141	transAT-PKS
		BN25_1__3078	14.1	1	2550	RiPP-like
BN25_1.20	*Chitinophaga* sp.	BN25_1__14885	78.1	1	3453	NRPS
		BN25_1__21571	113.1	1	15003	NRPS
		BN25_1__22164	114.1	1	3308	NRPS
		BN25_1__25211	125.1	1	3529	NRPS-like

		BN25_1__26943	130.1	11501	26395	terpene
		BN25_1__30663	148.1	1	8708	T1PKS/NRPS
		BN25_1__32573	159.1	1	11670	terpene
		BN25_1__43133	214.1	6224	27162	terpene
		BN25_1__47506	236.1	1	4783	terpene
		BN25_1__47958	239.1	26855	45772	terpene
		BN25_1__49892	249.1	1	4402	T1PKS/NRPS-like
		BN25_1__53797	269.1	1	9556	T3PKS
		BN25_1__54100	274.1	1	17827	arylpolyene
		BN25_1__56736	287.1	1	6452	arylpolyene
		BN25_1__57941	296.1	1	5323	RiPP-like
		BN25_1__68336	359.1	1	9874	resorcinol
		BN25_1__73902	386.1	1	14515	NRPS
		BN25_1__74837	394.1	3788	14915	RiPP-like
		BN25_1__75766	398.1	1	5177	NRPS
		BN25_1__78770	413.1	1	7101	terpene
		BN25_1__87738	462.1	12370	37770	lanthipeptide class I
		BN25_1__90506	480.1	1	14632	T1PKS/NRPS
		BN25_1__98044	508.1	1447	22235	terpene
		BN25_1__102071	538.1	1	5206	NRPS-like/T1PKS
		BN25_1__109233	575.1	1	7461	RiPP-like
BN25_1.23	*Labilithrix* sp.	BN25_1__6446	6.1	1	7017	RiPP-like
		BN25_1__17859	16.1	37892	49352	RiPP-like

		BN25_1__26191	26.1	83301	124476	T3PKS
		BN25_1__32052	32.1	245320	287326	arylpolyene
		BN25_1__35313	37.1	56768	89556	arylpolyene
		BN25_1__41839	43.1	1	15347	redox-cofactor
		BN25_1__55292	58.1	21494	64065	HR-T2PKS
		BN25_1__57659	63.1	21497	64065	RRE-containing
		BN25_1__63773	68.1	3421	23705	terpene
		BN25_1__63773	68.2	41861	62793	NRPS-like
		BN25_1__97763	93.1	71219	116958	T3PKS
		BN25_1__104157	99.1	20289	61587	RiPP-like
		BN25_1__114710	107.1	22573	33478	terpene
		BN25_1__47621	93.1	1	16239	redox-cofactor
		BN25_1__66183	132.1	2871	8609	RiPP-like
BN25_1.33	c__Bacilli	BN25_1__67791	134.1	1	17005	terpene
		BN25_1__97671	189.1	1	19047	T3PKS
		BN25_1__101859	199.1	1	9856	terpene
		BN25_2__13793	74.1	14674	26605	terpene
		BN25_2__16205	90.1	1	14957	arylpolyene
		BN25_2__46924	228.1	1	6086	RiPP-like
BN25_2.6	*Ralstonia pickettii*	BN25_2__54584	268.1	1	5412	redox-cofactor
		BN25_2__60037	191.1	1	3903	NI-siderophore
		BN25_2__102866	457.1	1	11269	betalactone
BN25_2.14	f__Xanthobacteraceae	BN25_2__6507	6.1	25536	45988	RRE-containing

		BN25_2__27808	21.1	62271	83122	terpene
		BN25_2__46286	30.1	80875	104120	lanthipeptide class II
		BN25_2__64751	50.1	117882	128652	RiPP-like
		BN25_2__74182	58.1	1	43198	NRPS-like/terpene
		BN25_2__75458	59.1	1	44749	NRPS
		BN25_2__75458	59.2	55638	83235	betalactone
		BN25_2__84700	65.1	1	22616	NRPS
		BN25_2__84700	65.2	129778	140587	RiPP-like
		BN25_2__106798	84.1	31541	42416	RiPP-like
		BN25_2__106798	84.2	67102	109801	NRPS-like
		BN25_2__109722	86.1	18639	42070	redox-cofactor
BN25_3.6	*Limosilactobacillus* sp.	BN25_3__51630	37.1	1	8060	T3PKS
		BN25_3__72854	4.1	364336	385193	terpene
BN25_3.8	*Afipia* sp.	BN25_3__104477	6.1	71174	91788	hserlactone
		BN25_3__104477	6.2	262834	287793	terpene
		BN25_3_7576	5.1	1	15691	T3PKS
		BN25_3_14843	16.1	43445	15141	RiPP-like
		BN25_3_25464	22.1	1	28173	NAPAA
		BN25_3_25464	22.2	81471	179404	NRPS-like/T1PKS/NRPS
BN25_3.10	*Mycobacterium aubagnense*	BN25_3_44007	36.1	261418	283791	NRPS-like
		BN25_3__44735	37.1	1	3827	NRPS-like
		BN25_3__45434	39.1	23681	74813	T1PKS
		BN25_3__51530	45.1	26980	91554	NRP-metallophore/NRPS

		BN25_3__68536	58.1	23029	37873	NAGGN
		BN25_3__88481	68.1	35835	81363	T1PKS
		BN25_3__96050	74.1	11047	33751	redox-cofactor
		BN25_3__97353	77.1	149180	218498	T1PKS/NRPS-like
		BN25_3__99076	80.1	7194	43281	NRPS-like
		BN25_3__108033	88.1	1	28751	T1PKS
		BN25_3_9443	3.1	101469	123925	cyanobactin
		BN25_3_19266	5.1	279159	304148	terpene
		BN_25_3_23688	7.1	74403	116379	NRPS-like
		BN25_3_48990	10.1	338253	362999	betalactone
		BN25_3_48990	10.2	529574	540473	RiPP-like
		BN25_3_48990	10.3	715442	737756	redox-cofactor
BN25_3.17	*Bradyrhizobium* sp.	BN_25_3_49891	11.1	144155	164811	hserlactone
		BN25_3_52015	12.1	89099	130988	NRPS-like
		BN25_3_67227	15.1	296416	337477	T3PKS
		BN25_3_80694	17.1	4309	28040	LAP
		BN25_3_80694	17.2	52914	73777	terpene
		BN25_3_91870	18.1	84470	95285	RiPP-like
		BN25_3_108991	22.1	246404	267036	hserlactone

Denote: the contig number and region classification was generated by antiSMASH.
