# Supplementary figures and images for: Metagenomics and Metagenome-Assembled Genomes: Analysis of Cupei from Sichuan Baoning Vinegar, One of the Four Traditional Renowned Vinegars in China
Source: Foods. 2025 Jan 26;14(3):398. doi: 10.3390/foods14030398 (PMC11816609; doi:10.3390/foods14030398)

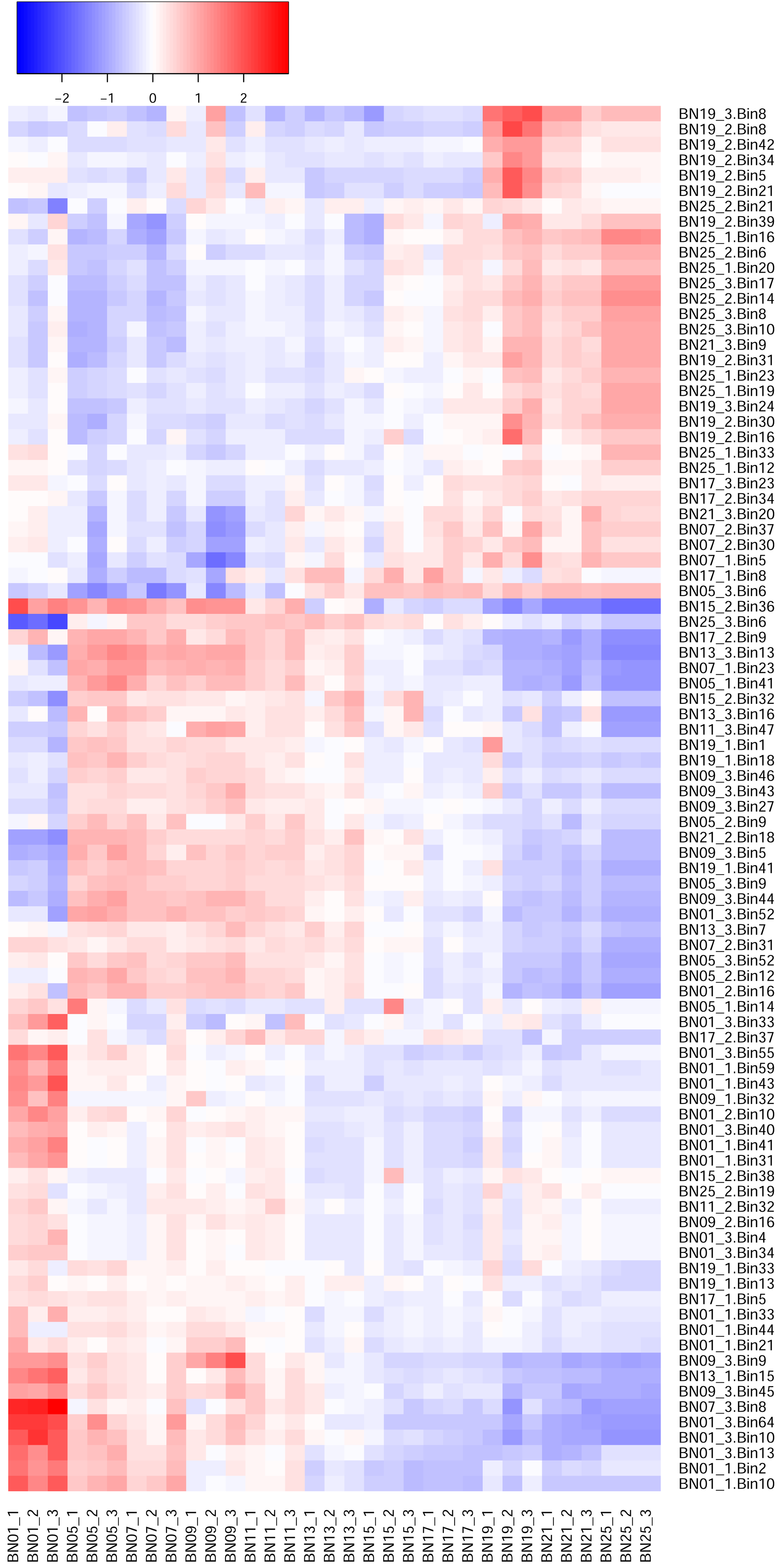

Supplement: Supplementary file 1 [file foods-14-00398-s001.zip › Figure S1.tif]
